# Elucidating the Reciprocal Interplay between Supramolecular Polymer Cytoskeletons and Fuel-Dependent Synthetic Cells

**DOI:** 10.1021/jacs.6c09705

**Published:** 2026-08-12

**Authors:** Nils Bäumer, Leonie Kauling, Benedikt Kirmayer, Simone M. Poprawa, Arthur Neuberger, Job Boekhoven

**Affiliations:** † Department of Bioscience, School of Natural Sciences, 9184 Technical University of Munich, Lichtenbergstrasse 4, 85748 Garching, Germany; ‡ Walther-Straub-Institute of Pharmacology and Toxicology, LMU Medizin, Ludwig-Maximilians-Universität München, Goethestrasse 33, 80336 Munich, Germany

## Abstract

Synthetic cells are compartments designed to mimic the functions and characteristics of living cells. By constructing synthetic cells from abiotic, basic components (bottom-up), it is possible to investigate the minimal requirements for life and gain insights into fundamental principles of biology. Among the available platforms, complex coacervates are particularly attractive, due to their potential to encapsulate a wide range of biomolecules and other cargo, enabling genotype-phenotype mapping. By coupling coacervate formation to a fueled chemical reaction cycle, the synthetic cells become fuel-dependent, growing in the presence of fuel and decaying in its absence, resembling biological cells. However, constructing cellular substructures for these fuel-dependent synthetic cells, such as cytoskeletons, has remained an unresolved challenge. Here, we show that supramolecular (co)­polymers can act as cytoskeletons for the synthetic cells, depending on their condenophilicity (their affinity for the droplet phase). By using two distinct supramolecular building blocks, the condenophilicity can be modulated. Supramolecular copolymers where only some of the monomers bind to the complex coacervate result in the formation of a protruding cytoskeleton in and around the droplet. However, as condenophilicity increases, the polymers partition strongly into the coacervates, leading to the formation of a fully encapsulated cytoskeleton. We found that these internal structures influence the synthetic cell properties, such as morphology and lifespan. Moreover, the synthetic cells can dynamically reconstitute the supramolecular fibers, creating distinct populations within the cells and the surrounding dilute phase. Our results demonstrate that orthogonally assembled structures can serve as cellular substructures for active complex coacervate-based synthetic cells, broadening the existing arsenal of tools to bestow these rudimentary synthetic cells with more life-like properties.

## Graphical abstract

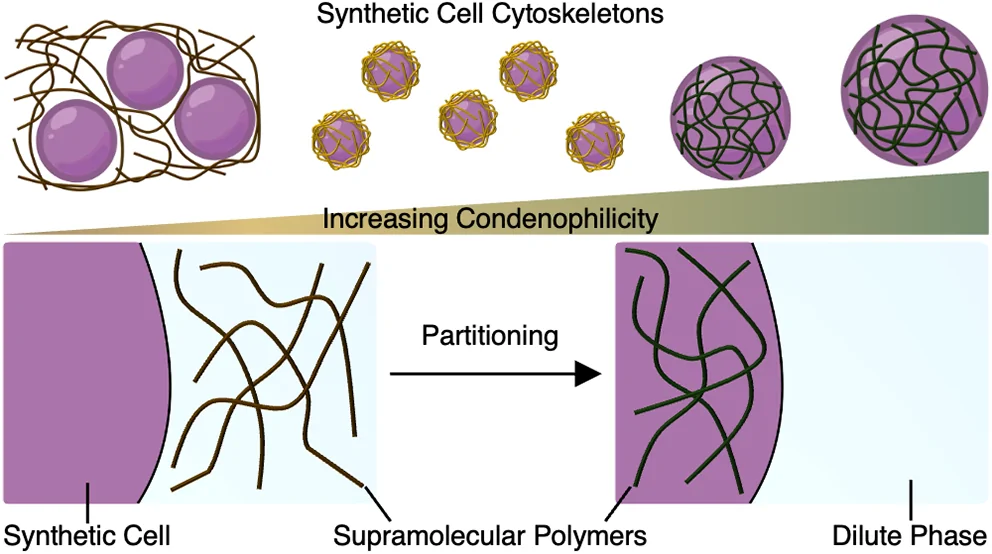

## Introduction

The *de novo* synthesis of life has traditionally been approached from two starting points—the top-down approach seeks to modify existing cells and reduce their components to the minimum,^1–4^ while the bottom-up approach involves assembling cellular compartments and functions from scratch.^5–10^ For both approaches, lipid membranes have been the traditional choice for compartmentalization, as they are also biology’s preferred method.^11–13^ Since lipid-based membranes are robust, they do not readily allow molecules to enter or exit without complex channels and pumps.^14–17^ For this reason, synthetic cells based on liquid–liquid phase separation have recently gained in popularity. Among the available platforms, complex coacervates offer particularly attractive properties.^18–20^ These microdroplets comprise oppositely charged molecules, forming a dense, water-rich phase that readily takes up macromolecules, including biomolecules and other cargo.^21–23^ Moreover, their emergence can be triggered using a wide range of inputs.^24–28^

Most of the described synthetic cells based on complex coacervates are thermodynamically stable.^29–41^ By contrast, life requires a constant transduction of energy to prevent relaxation to the thermodynamic minimum.^42^ To bridge this divide, we have developed fuel-dependent complex coacervate-based synthetic cells. In these fuel-dependent synthetic cells, the consumption of a chemical fuel, such as 1-ethyl-3-(3-(dimethylamino)­propyl)­carbodiimide (EDC), activates a peptide (Scheme 1A).^43–48^ The active peptide can bind a polyanion, forming complex coacervate droplets with other activated peptides. However, the peptide rapidly deactivates, with a half-life of about a minute, after which it leaves the droplet again. This creates a dynamic ensemble in which synthetic cells emerge and grow when fuel is present. But as they deplete the fuel, deactivation outcompetes uptake of freshly activated droplet material, causing the droplets to shrink. Consequently, during periods of starvation, these synthetic cells slowly decay until they ultimately dissolve, completing their lifecycle. By tuning the individual droplet components or using different cargos, such as small-molecule autocatalysts or DNA, we have shown that various hallmarks of life can be realized, including division-induced offspring production and primitive genotype-phenotype coupling.^21,49,50^

**Scheme 1:**
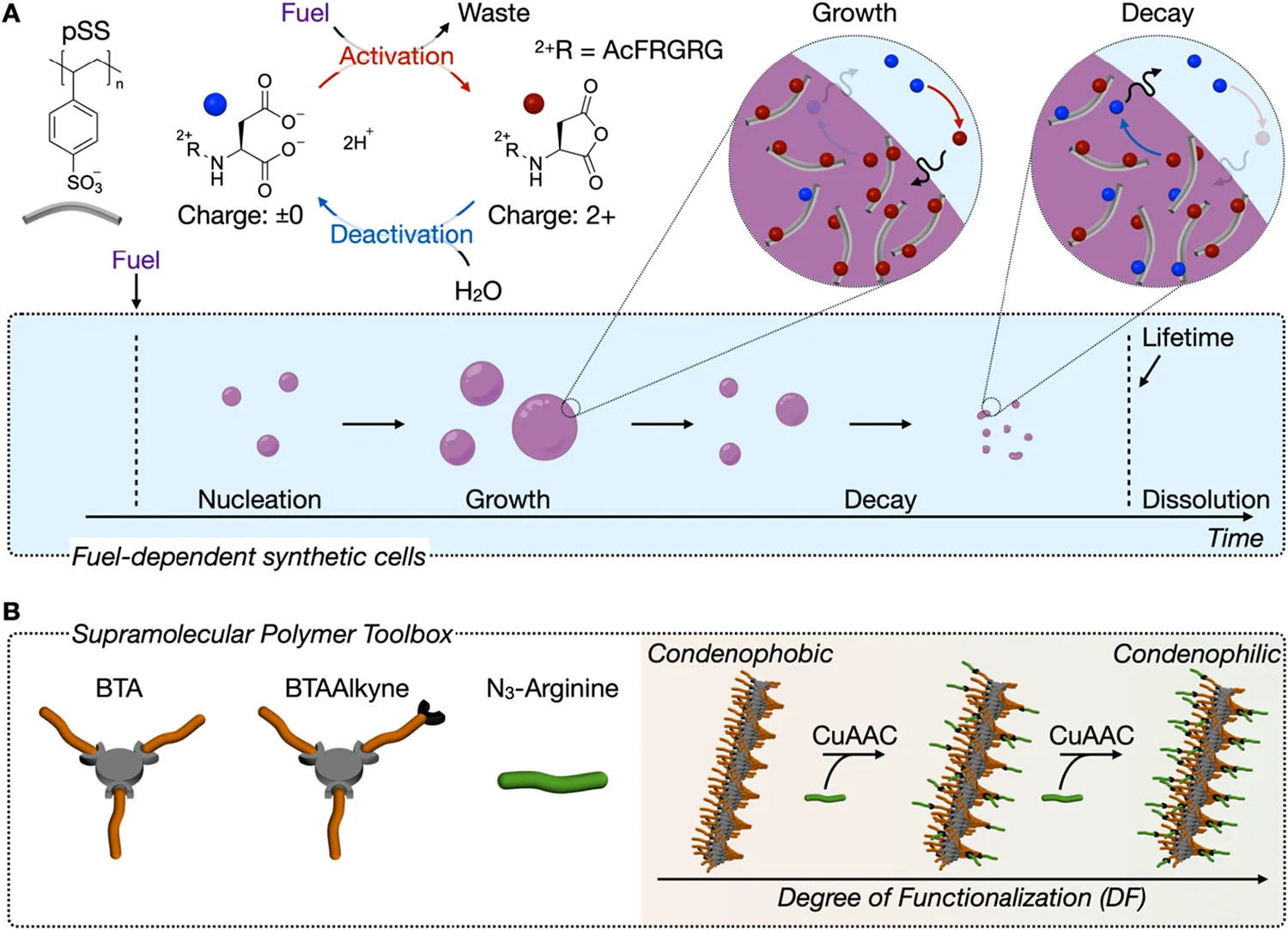
Design of the Fuel-Dependent Synthetic Cell (A) and Supramolecular Cytoskeleton (B) Platform Used in this Study^a^

a (A) Schematic depiction of the chemical reaction cycle and phase-separation process of the synthetic cell platform. (B) Benzene tricarboxamide-based supramolecular polymer toolbox to synthesize supramolecular (co-)­polymers of varying condenophilicity.

In biological cells, different functions are regulated by different organelles.^51,52^ For instance, the nucleus stores genetic material and directs cellular activities,^53^ while ribosomes are responsible for protein synthesis, and the endoplasmic reticulum for lipid synthesis.^54–56^ By dividing cellular functions among different organelles, cells can facilitate processes that are chemically or biochemically incompatible, such as protein assembly and degradation.^57–60^ The realization of distinct cellular substructures within active complex coacervates has remained a hitherto unresolved challenge and presents a bottleneck to the further diversification of this class of phase-separated compartments as a synthetic cell platform.

In this work, we therefore demonstrate that self-assembled supramolecular polymers can fulfill the role of a rudimentary cellular substructure in coacervate-based synthetic cells. As a platform for the supramolecular polymer, we used the one-dimensional assemblies formed by benzene tricarboxamides (Scheme 1B).^61–64^ We designed these to tune their affinity for the active coacervate-based synthetic cells. Specifically, our supramolecular polymer is a coassembly of two building blocks—one has only condenophobic ethylene glycol chains (BTA, Figure 1A),^65^ while the second is almost identical except for a terminal alkyne on the periphery of one of its condenophobic ethylene glycol chains (BTAAlkyne, Figure 1A). The terminal alkyne enables us to functionalize the supramolecular copolymer, in-situ, using Cu-catalyzed azide–alkyne cycloaddition (CuAAC).^66,67^ As a reaction partner, we use an azide-functionalized arginine derivative, which preferentially partitions into the droplet compartments.^21^ By varying the ratio between the different building blocks and, in turn, the degree of functionalization (DF), the condenophilicity of the ensemble can be modulated.^68–70^ Crucially, this variation affects only the ratio of the two building blocks in the ensemble, not the degree of functionalization per molecule. Our results indicate that although functionalized polymers generally interact with the synthetic cells, the mechanism of interaction varies with the DF. At low functionalization, the supramolecular polymers interact with the droplet material but do not fully partition, resulting in a protruding BTA fiber structure within and around the droplets. This structure affects the synthetic cells by inhibiting their fusion, leading to smaller droplets that are more stable against dissolution. As the degree of functionalization increases, the extent of supramolecular polymer partitioning increases, altering the fuel-dependent synthetic cell behavior. Under these conditions, the droplet lifetime increases due to the stabilizing effect of the supramolecular polymer within the droplet, akin to a cytoskeleton. Taken together, we demonstrate that supramolecular polymers can serve as distinct types of cytoskeletons that modulate the behavior of their host.

**Figure 1:**
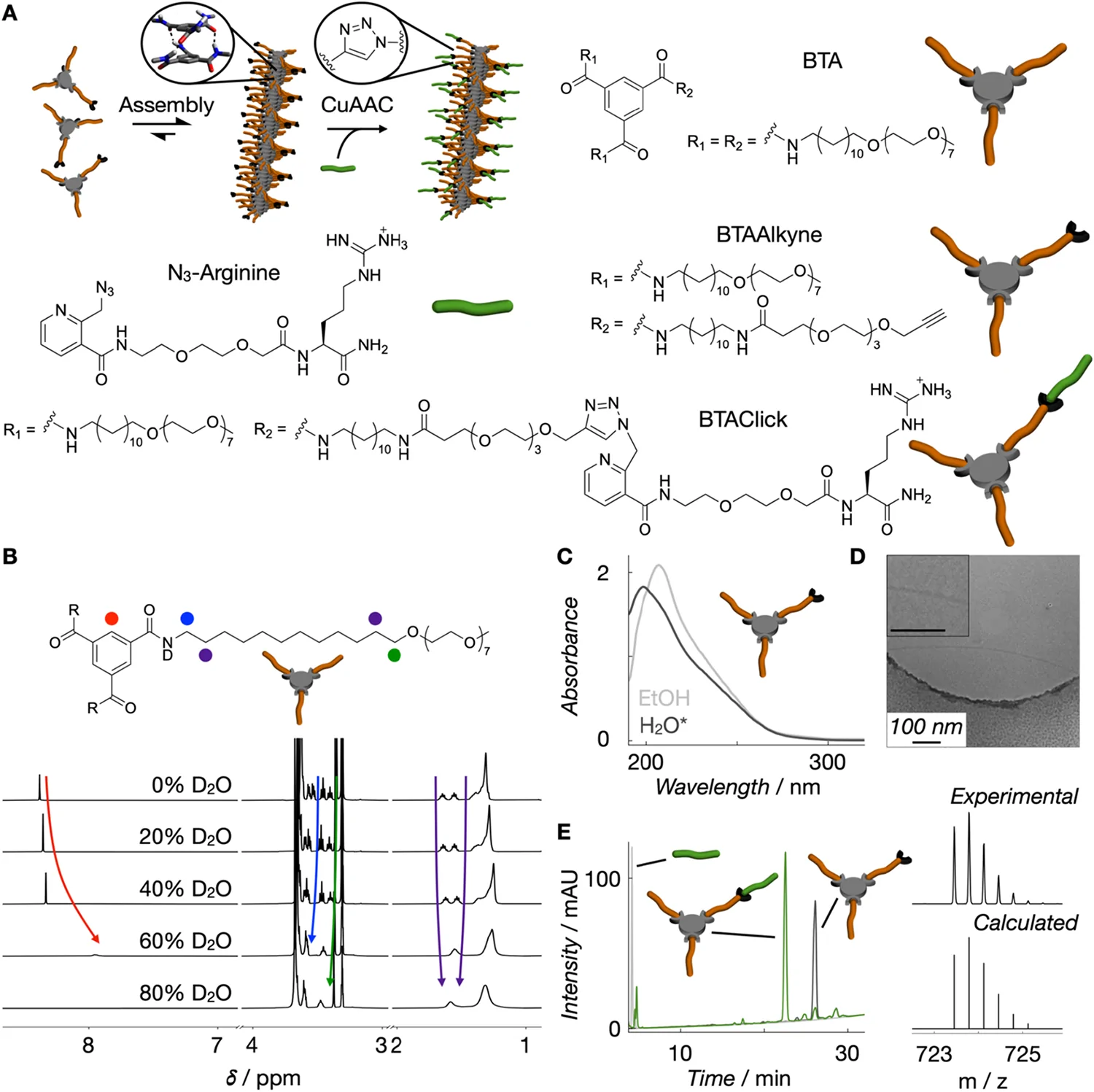
Postassembly modification of BTA-based supramolecular polymers. (A) Molecular structure of BTA, BTAAlkyne, N_3_–Arginine, and BTAClick and a schematic representation of their sequential self-assembly and functionalization. (B) Partial solvent-dependent NMR studies of BTA in binary mixtures of D_2_O and MeOH-*d*_4_ at *c* = 1 mM and *T* = 25 °C. (C) Solvent-dependent UV–vis absorption spectra of BTAAlkyne at *c* = 0.5 mM and *T* = 25 °C in EtOH (light gray) and in a 5-vol % EtOH aqueous solution. (D) Cryo-TEM micrograph of the supramolecular assemblies of BTAAlkyne formed at *c* = 0.2 mM in a 5-vol % EtOH aqueous solution. (E) HPLC traces of N_3_–Arginine (light gray), BTAAlkyne (dark gray), and the CuAAC reaction mixture (green) with the HR-MS spectrum of the isolated BTAClick shown; the *m*/*z* ratio was calculated for the ionized species (M+H+Na+K)^3+^.

## Results and Discussion

Both BTA and BTAAlkyne were synthesized following reported procedures^71,72^ and characterized by ^1^H and ^13^C nuclear magnetic resonance spectroscopy (NMR) and high-resolution mass spectrometry (HR-MS; see Supporting Information for synthetic details and characterization data). We expected that BTA and BTAAlkyne form one-dimensional supramolecular polymers in aqueous media, stabilized by aromatic stacking and hydrogen bonding,^73–76^ which we probed by solvent-dependent ^1^H NMR of BTA in binary mixtures of D_2_O and MeOH-*d*_4_ (Figure 1B). The aromatic proton signal shifted upfield alongside peak broadening with increasing solvent polarity, indicating aromatic interactions (Figure 1B, red arrow).^77,78^ Moreover, the signals corresponding to the aliphatic spacer between the benzene core and the solubilizing ethylene glycol groups lost their fine structure when the water content exceeded 40%, coinciding with the spectral shift of the aromatic proton signal. These combined data are consistent with the formation of a hydrophobic alkyl shell around the central benzene stack within the supramolecular polymer, as previously observed for related supramolecular assemblies.^79,80^ By solvent-dependent ultraviolet–visible (UV–vis) spectroscopy, we found identical changes in absorption induced by the self-assembly of BTA and BTAAlkyne, namely a hypsochromic shift of the absorption maximum, matching the expected face-to-face packing arrangement of the chromophores (Figures 1C and S2).^81,82^ Additionally, we observed a minor hypochromic shift in the absorption maximum, typical of shielding of the aromatic chromophore from the solvent medium.^83,84^ Importantly, we found that these spectral changes upon self-assembly remained present in MES-buffered water (Figure S2), which was later required for the fuel-dependent synthetic cells. Moreover, we found that the cooperative supramolecular polymerization mechanism of both assemblies is identical, with a highly similar thermodynamic driving force (Figure S3 and Table S1). By cryogenic transmission electron microscopy (cryo-TEM), we found that BTA and BTAAlkyne had assembled into flexible fibers measuring around 7.0 nm in diameter and up to multiple microns in length, matching the dimensionality of single columnar stacks previously reported for structurally related compounds (Figures 1D and S2).^72^ Importantly, no distinct fiber populations were observed in mixed samples of BTA and BTAAlkyne (Figure S2E). Taken together, both BTA and BTAAlkyne assembled into fibers with identical packing within the polymers, thereby facilitating their supramolecular copolymerization.^69,85–87^

Next, we probed the postassembly functionalizability of the BTAAlkyne fibers by high-performance liquid chromatography (HPLC) and HR-MS (Figure 1D). Upon addition of Cu^2+^ to a mixture of preassembled BTAAlkyne, N_3_–Arginine, and ascorbic acid, we found a near-quantitative depletion of the BTAAlkyne peak within the first minute, after Cu^2+^ addition (Table S2), highlighting that this reaction is chemically compatible with our EDC reaction cycle and also operates on similar time scales.^88,89^ A new peak emerged at lower retention times than that of BTAAlkyne, suggesting that this species is more polar than the starting material (Figure 1E). HR-MS further confirmed that this species corresponds to the reaction product of the CuAAC (hereafter referred to as BTAClick).

Next, we used the BTA supramolecular polymers as cytoskeletons in our fuel-dependent synthetic cells. These synthetic cells are based on complex coacervates that comprise two building blocks—a polyanion, polystyrenesulfonate (pSS, 4.3 kDa), and a short peptide (Ac-FRGRGD–OH, RG_2_D, Figure 2A). From our previous work, we know that short pSS derivatives tend to form more liquid-like coacervate phases, making them more suitable as a synthetic cell platform.^43,48^ The peptide has an overall neutral charge in its inactive state under our conditions (200 mM MES buffer, pH = 5.3). Upon activation by EDC as fuel, the peptide’s C-terminal aspartic acid is converted to its corresponding anhydride, increasing the net charge from zwitterion, but charge neutral (±0) to +2. The cationization enables the activated peptide to bind the polyanion and form a complex coacervate with other activated peptides (Figure 2A). Yet, the activated peptide is thermodynamically unstable and hydrolyzes with a half-life of about a minute to yield the original peptide with low polyanion affinity. Thus, these fuel-dependent droplets constantly take up newly activated peptide and efflux deactivated droplet material, leading to their decay when fuel is not continuously supplied. Indeed, when we add fuel to the peptide-polyanion mixture, we observe turbidity that decays as the droplets deplete the fuel (control in Figure 2C). We probed the influence of the supramolecular polymers on the droplet lifespan. In the absence of supramolecular polymers, the turbidity reaches its maximum within 1 min (Figure 2C). Afterward, the optical density gradually decreases over approximately 8 min, followed by a more rapid decrease toward the end of the life cycle. In the presence of homopolymers of BTA, i.e., a DF of 0%, this behavior remained unchanged, and we found no statistically significant differences in the droplet lifespan (defined as ΔOD (600 nm) < 0.25). This finding is already significant, as it shows that the polymers are, on the one hand, chemically compatible with the droplets but do not significantly alter their behavior as crowding agents.^90–92^ On the other hand, it also indicates that our molecular design was correct, as the ethylene glycol solubilizing chains are considered condenophobic and do not “interfere” with the droplets. We further verified this claim by spinning down the droplets and measuring the distribution of BTA polymers between the pellet and the supernatant. At DF = 0% (i.e., pure BTA fibers), the BTA molecules preferentially accumulate in the dilute phase (Table S3) with a partitioning coefficient *K*_p_ = 0.47 ± 0.02 (*K*_p_ = 1 means equal distribution).

**Figure 2:**
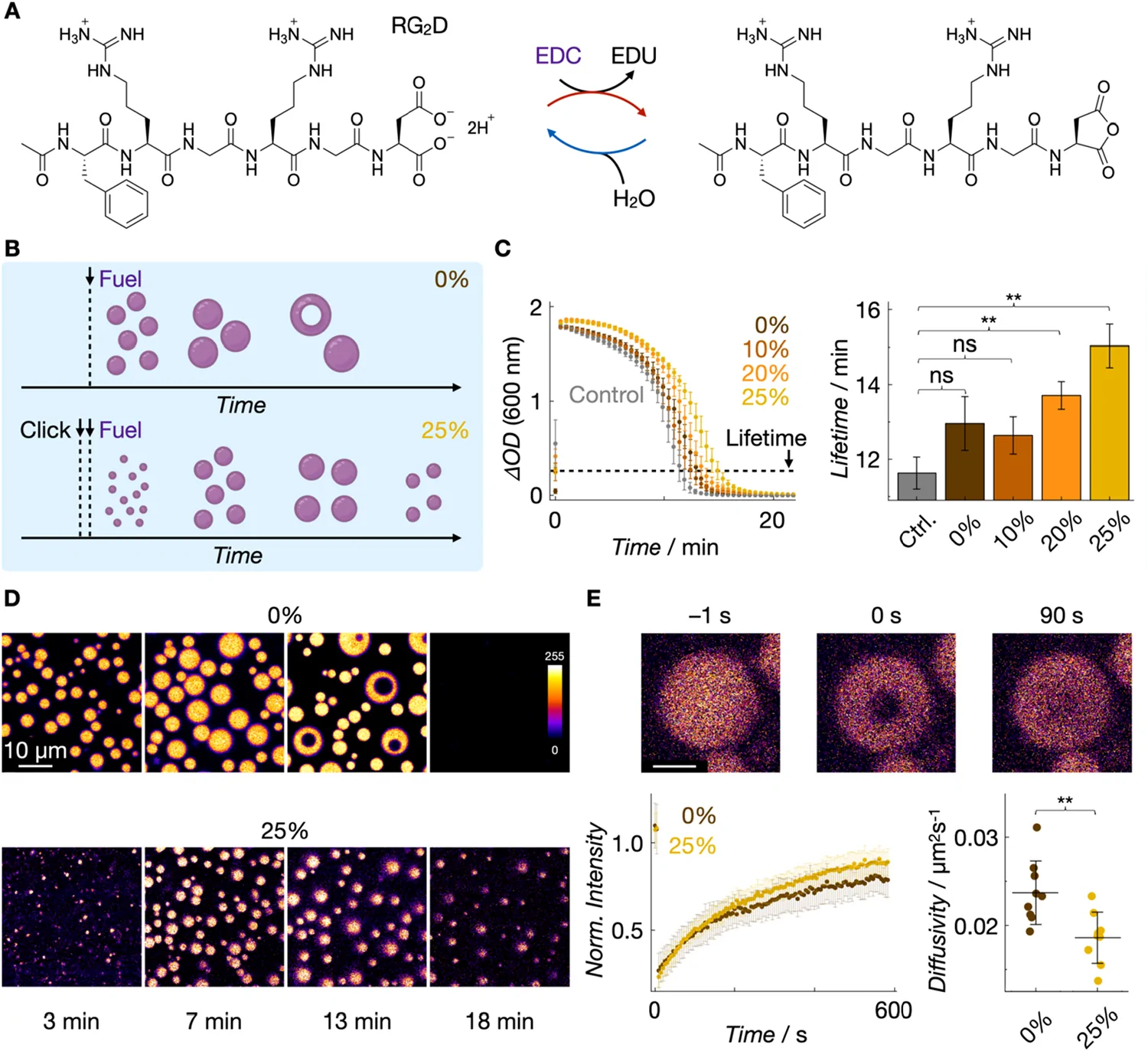
The degree of BTA functionalization affects their condenophilicity and the droplet properties. (A) Chemical structure of RG_2_D and its corresponding anhydride. (B) Schematic depiction of the experimental design and the observed droplet morphologies at DF = 0 and 25%. (C) DF-dependent turbidity traces at λ = 600 nm plotted against time after EDC addition to a mixture of RG_2_D (12.3 mM), pSS (20.0 mM), N_3_–Arginine (0.5 mM), CuSO_4_ (0.5 mM), ascorbic acid (2.0 mM), and the BTA copolymers (total c = 1.0 mM, DF based on mol %) in 5-vol % EtOH/200 mM MES buffer (pH = 5.3) with the extracted lifetime based on an optical density threshold of ΔOD < 0.25, with the control experiment in the absence of any supramolecular polymer or CuAAC component. Standard deviations are based on triplicate experiments. (D) Time-dependent snapshots of confocal microscopy studies conducted under the same experimental conditions as the turbidity traces shown in (C). Droplets are stained using NBD-functionalized RG_2_N (c = 0.5 μM), all images share the same scale bar. (E) Snapshots of FRAP experiments of Cy3-labeled pSS (c = 0.5 μM, 10.8 kDa) at DF = 25% (all images share the same scale bar corresponding to 10 μm) with average FRAP intensity traces for DF = 0 and 25% and the extracted diffusivity shown below (3 technical replicates of 3 chemical replicates, *n* = 9), using identical experimental conditions as in (C). Statistical analysis was performed using a two-tailed *t* test assuming unequal variance; ns, not significant (*p* > 0.05), *p < 0.05, **p < 0.01, and ***p < 0.001.

Next, we increased the degree of functionalization by simultaneously adding Cu^2+^ and the fuel. To disentangle the influence of the total positive charge in the system from that of the polymer, the concentration of all CuAAC reagents, including N_3_–Arginine, is kept constant in these experiments. We found that, for one, the lifetime steadily increased from 11.6 ± 0.4 min in the control experiments to a maximum of 15.0 ± 0.6 min, at DF = 25% (Figure 2C, Table S4). Additionally, the initial decrease in optical density was slower than in both the control experiments and those with BTA homopolymers, suggesting changes in the droplets’ settling and fusion behavior. Both observations indicated that, as the condenophilicity of the supramolecular polymer increases, the supramolecular polymers began interacting with the fuel-dependent synthetic cells. Moreover, we found that these trends scale with the total concentration of the supramolecular copolymer, when DF is kept constant (Figure S4, Table S5). To corroborate the generalizability of condenophilicity-driven interactions, we further tested whether negatively charged BTA fibers can achieve a similar effect. To our satisfaction, a near-identical increase in lifetime was observed when the reaction was carried out with a negatively charged azide precursor (Figure S5).

To ensure the BTA supramolecular polymers did not interfere with the kinetics of activation and deactivation, we quantified the anhydride concentration after EDC addition. Satisfactorily, no change in activation or hydrolysis rates was observed in the presence of the functionalized BTA fibers (Figure S6), irrespective of their DF.

We imaged the synthetic cell’s behavior using confocal fluorescence microscopy. After the addition of fuel and Cu^2+^, we observed the emergence of droplets that sank to the surface of the microscopy chamber within a few minutes (Figure 2D). Over the following minutes, the droplets grew due to the influx of activated material and fusion.^93–96^ This process corresponded to the modest change in absorbance observed during the first 8 min of the optical density measurements. After about 8 to 10 min, the droplet decay became more pronounced, accompanied by the formation of vacuoles^97^ and, ultimately, complete dissolution. When we conducted the same experiment with supramolecular polymers of DF = 25%, the behavior was drastically different. Conventionally, increases in the lifetime of fuel-dependent synthetic cells are correlated with larger droplets—the larger, the longer it takes to dissolve. Surprisingly, in our fuel-dependent synthetic cells with a supramolecular polymer, the droplets were smaller at DF = 25%. Moreover, the droplets took considerably longer to settle after nucleation, likely because of their smaller size. Furthermore, they tended to fuse less often, which could explain the smaller droplets. Finally, the increase in lifetime was evident: the small droplets remained present even after all the droplet material had dissolved in control experiments. The change in the settling and fusion behavior suggests that the constitution of the droplets’ surface may be changed due to the interaction with the supramolecular polymer.

To understand the interactions between the coacervates and the BTA polymers, we performed fluorescence recovery after photobleaching (FRAP) experiments using a fluorescently labeled pSS derivative (Figures 2E andS7) and a proxy for the activated peptide (Figure S8) to identify which coacervate component is involved in the supramolecular polymer interaction. This analysis revealed that the pSS diffusivity decreased by a moderate 19% (*p* = 0.0041, Tables S6 and S7) in the presence of supramolecular copolymers (DF = 25%), while the peptide diffusivity showed no statistically significant change (Tables S8 and S9). This observation is consistent with expectations, as the supramolecular polymer becomes positively charged upon functionalization, thereby favoring interactions with the negatively charged polyanion. In contrast, the diffusivity of the peptide remained unaffected, as the total concentration of pSS exceeded the combined concentration of the peptide and the functionalized supramolecular polymer (Figure S8). Accordingly, enough pSS is available to bind all existing activated peptides, irrespective of interactions between the pSS and the BTA copolymer surface. We conclude that coassembly with BTA prevents stronger interactions and binding between the positively charged BTAClick and pSS, resulting in only moderate changes in diffusivity.

From the experiments above, it is clear that the 1.0 mM supramolecular fibers with 25% functionalization affected the morphology of the synthetic cells. We wondered whether this observation resulted from the 1.0 mM fibers with DF = 25% or whether it was simply the cationic BTAClick that did all the work. To test this, we added 0.25 mM pure BTAClick fibers at DF = 100%. To our surprise, we found that the fuel-dependent synthetic cells in this environment had an insignificantly different lifespan compared to the control (Figure 3A). We deduce from this observation that although the condenophobic BTA does not directly interact with the droplet material, its presence in the copolymer is critically important to induce the observed increase in the droplet lifetime. We corroborated this further by spin-down experiments, which revealed an increase in the partitioning coefficient of BTA from *K*_p_ = 0.47 ± 0.02 (DF = 0%) to 3.90 ± < 0.01 (Table S10). Put differently, the functionalization of the condenophobic BTA fibers with cationic azides drastically increases their condenophilicity to a degree that they now partition in the fuel-dependent synthetic cells. Additionally, this finding directly suggests that the different BTAs can act as comonomers and engage in social self-sorting, even in the presence of our synthetic cells.

**Figure 3:**
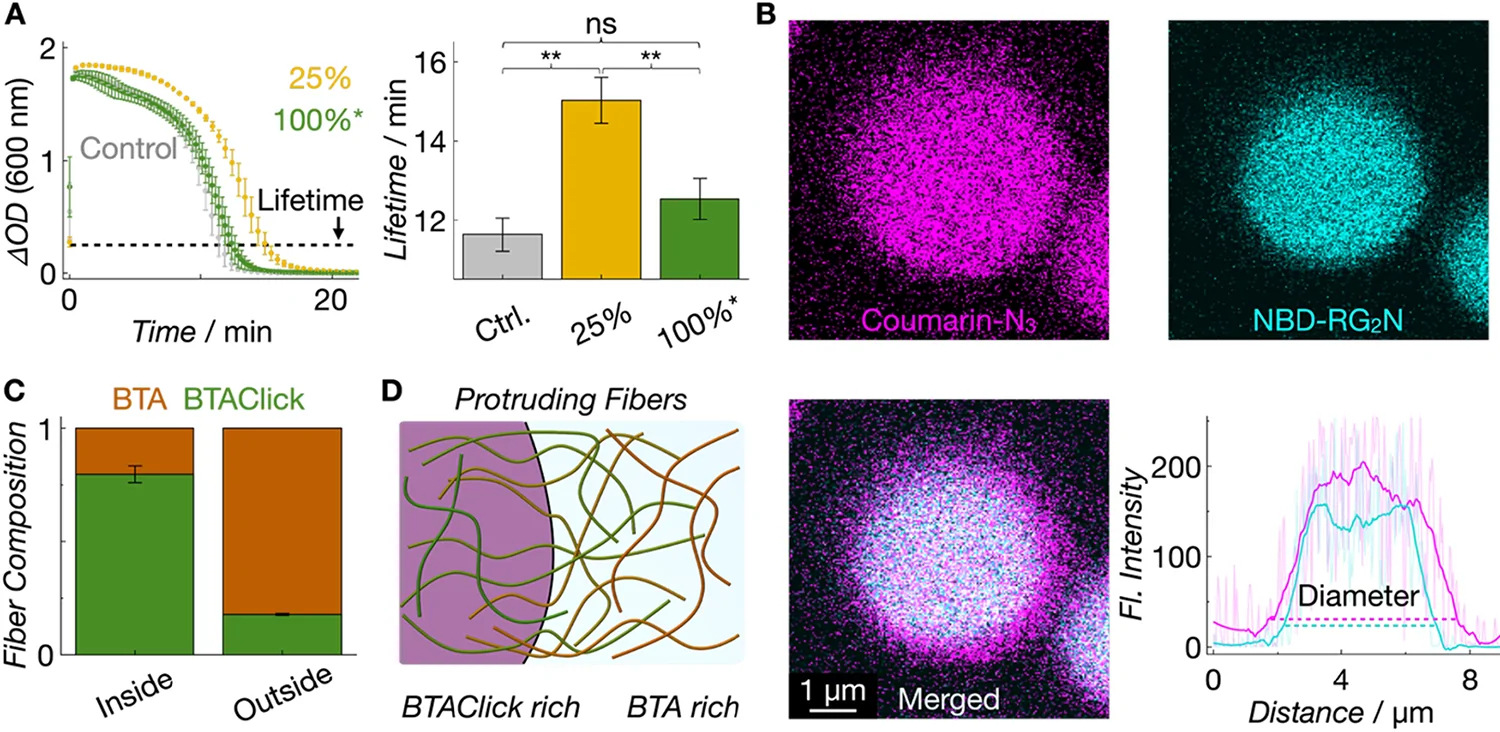
Reciprocal interactions between droplets and supramolecular copolymers. (A) DF-dependent turbidity traces at λ = 600 nm plotted against time after EDC addition to a mixture of RG_2_D (12.3 mM), pSS (20.0 mM) in isolation (control), in the presence of the copolymer (DF = 25%, c = 1 mM) and in the presence of the homopolymer of BTAClick (DF = 100%, c = 0.25 mM) in 5-vol % EtOH/200 mM MES buffer (pH = 5.3) with the extracted lifetime based on an optical density threshold of ΔOD < 0.25. Standard deviations are based on triplicate experiments. (B) Fluorescence microscopy images of droplets formed in the presence of supramolecular copolymers at DF = 25%, with the different droplet diameters based on fluorescence intensity being shown in the bottom right. The supramolecular polymers are stained using coumarin azide (magenta), while the droplet material is stained using NBD-functionalized RG_2_N (cyan). (C) Relative compositions of supramolecular polymers inside and outside of the droplets at DF = 25%, calculated based on HPLC; error bars are based on triplicates. (D) Schematic depiction of the proposed protruding fiber formation mechanism driven by the binding of BTAClick-rich material to the droplets and the extension of BTA-rich segments into the dilute phase.

Intrigued by the ability to tune the condenophilicity of the fibers by clicking on cationic building blocks, we employed a costaining approach, in which the droplet material is stained with NBD-functionalized peptide,^44^ and the BTA copolymer is stained with coumarin azide (Figure 3B). We found the dyes for the supramolecular polymers and droplets colocalized, and thus that the supramolecular polymers partitioned within the droplets. Interestingly, we found that when we analyzed droplet diameters in the peptide and supramolecular fiber-channel (see Supporting Information for the analytical workflow, Table S11), the apparent droplet size based on the stained BTA was around 20% larger than that of the peptide. This observation indicates that, while the functionalized copolymer can interact directly with the droplet and partition inside the phase-separated compartment, it can also extend into the surrounding dilute phase. This observation could explain the slower fusion and growth, as the copolymer extending into the solvent alters the droplets’ surface chemistry, which has been shown to have a considerable influence on the dynamic behavior within phase-separated condensed phases.^98^ Moreover, this observation reaffirms the supramolecular polymerization of the two building blocks, as the extension into the surrounding medium can only be explained by the condenophobicity of the BTA building block.

Next, we analyzed the dynamic interplay between the functionalized supramolecular copolymers and the droplets. Specifically, we wondered whether the supramolecular copolymers act as a classical static copolymer of condenophobic and condenophilic building blocks or as a dynamic supramolecular copolymer in which condenophobic and condenophilic building blocks can reshuffle to adapt to their environment, i.e., being inside or outside the droplet. To test this idea, we analyzed the composition of the supramolecular copolymers within the droplets and in the surrounding media by spinning down the droplets. We assume that when we spin down the droplet, we separate fibers within and around the droplets from those in the dilute phase. Interestingly, we found that the fibers in the droplets mostly comprise the condenophilic BTAClick, whereas the copolymers in the surrounding media are mostly composed of BTA (Figure 3C, Table S10). Assuming the fibers form homogeneously, this finding suggests that the synthetic cells can directly remodel the supramolecular polymer fibers, creating two distinct supramolecular polymer populations within the synthetic cells and outside.

We conclude that there is a reciprocal influence between the droplets and the BTA copolymers. Upon fuel addition, the droplets nucleate, and the existing fibers (DF = 25%) in solution partition into the droplet material, inhibiting their unhindered growth and fusion observed in the presence of only the condenophobic BTA (DF = 0%). In turn, the droplets start to remodel the fibers. The HPLC data showed that the click reaction was much faster than droplet formation. We thus conclude that the BTA and BTAClick initially form a random copolymer. During the lifecycle, condenophilic BTAClick accumulates in the droplets, whereas condenophobic BTA is expelled, leading to differences in fiber composition between the droplets and the surrounding medium. Whether fragments of fibers or individual monomers are exchanged between the phases remains unclear at this moment. Finally, from the confocal microscopy data, we conclude that the fibers extend well beyond the droplets, altering the droplet’s surface composition (Figure 3D). This protruding fiber structure ultimately allows the droplets to live longer, even though they are smaller compared to the control experiments, due to surface-induced changes in the exchange behavior between the compartment and the dilute phase. This change in exchange behavior can be considered reminiscent of the functional properties of a cell membrane.^99–101^

Surprisingly, when the degree of functionalization exceeded 25% (*c* = 1.0 mM), the lifetime decreased again, nearly reverting to the original levels observed in the absence of a supramolecular polymer (25–70%, Figures 4B, S9, and S10, Tables S12 and S13). Under these conditions, the influence of the supramolecular polymer cannot be differentiated from the influence of the individual components of the click reaction (Figure S10). We attribute this to the transition between two distinct mechanisms observed at low DF (<25%) and high DF (>70%). As the DF exceeds 70%, a second rise in lifetime is eventually observed that continues until a DF of 100% is reached, i.e., all supramolecular monomers are BTAClick (Figure 4A,B). Again, the concentration of all CuAAC reagents is kept constant in these experiments. Surprisingly, here, the observed lifetimes even exceeded those observed at DF = 25%, indicating that the mechanism of interaction between the coacervates and the supramolecular polymers is distinct from that under moderate functionalization (lifetime = 15.0 ± 0.6 min for 25% and 19.8 ± 0.3 min for 100%). Similarly, a concentration-dependent trend was observed. With higher concentrations eventually preventing the lifetime from rising further, likely due to the accumulating ascorbic acid (Figure S10). Moreover, the pronounced stability of the turbidity measurement during the first minutes of the reaction cycle at DF = 25% is not maintained at DF = 100%, indicating changes in droplet settling and fusion dynamics.

**Figure 4:**
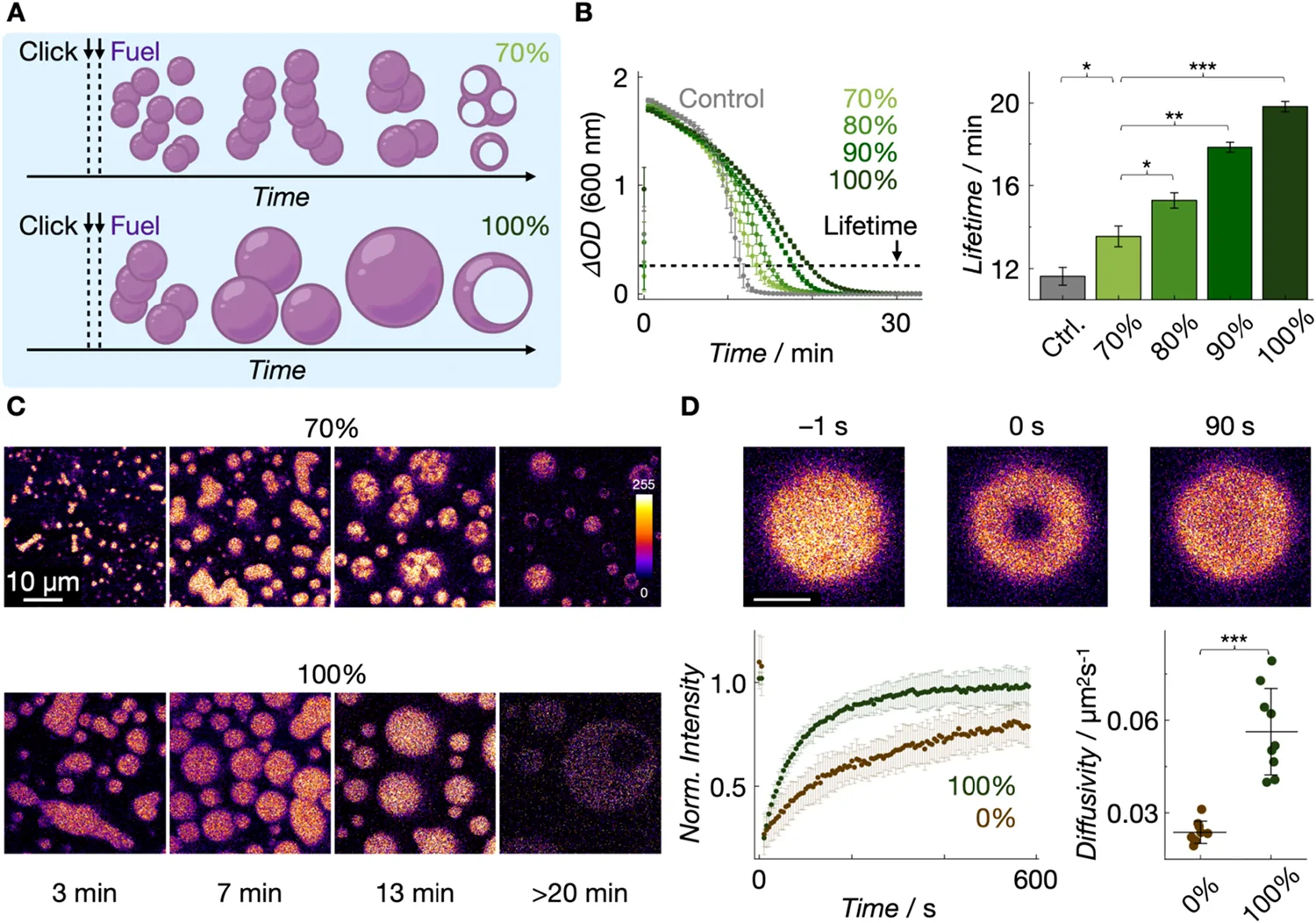
Evolution of fuel-dependent synthetic cells at high degree of functionalization (A) Schematic depiction of the experimental design and the observed droplet morphologies at DF = 70 and 100%. (B) DF-dependent turbidity traces at λ = 600 nm plotted against time after EDC addition to a mixture of RG_2_D (12.3 mM), pSS (20.0 mM), N_3_–Arginine (1.0 mM), CuSO_4_ (1.0 mM), ascorbic acid (4.0 mM), and the BTA copolymers (total *c* = 1.0 mM, DF based on mol %) in 5-vol % EtOH/200 mM MES buffer (pH = 5.3) with the extracted lifetime based on an optical density threshold of ΔOD < 0.25, with the control experiment in the absence of any supramolecular polymer or CuAAC component. Standard deviations are based on triplicate experiments. (C) Time-dependent snapshots of confocal microscopy studies conducted under the same experimental conditions as the turbidity traces shown in (B). Droplets are stained using NBD-functionalized RG_2_N (*c* = 0.5 μM), all images share the same scale bar. (D) Snapshots of FRAP experiments of Cy3-labeled pSS (*c* = 0.5 μM, 10.8 kDa) at DF = 100% (all images share the same scale bar corresponding to 10 μm) with average FRAP intensity traces for DF = 0 and 100% and the extracted diffusivity shown below (3 technical replicates of 3 chemical replicates, *n* = 9), using identical experimental conditions as in (B). Statistical analysis was performed using a two-tailed *t* test assuming unequal variance; ns, not significant (*p* > 0.05), *p < 0.05, **p < 0.01, and ***p < 0.001.

To corroborate the generalizability of these findings, we conducted control experiments at varying salt concentrations and pH (Figures S11 and S12). As our results indicate, the stabilizing effect of the supramolecular polymer fibers at DF = 100% remains intact across a wide range of experimental conditions. However, as our kinetic analysis has shown, the fibers do not interfere with the chemical reaction cycle (Figure S6). Consequently, when the reaction cycle operates suboptimally (above pH 7.5), our polymer fibers are unable to drive phase separation. Moreover, the addition of a good solvent for the BTA derivatives (tert-butanol) can negate the stabilizing effects due to depolymerization (Figure S13), further highlighting that supramolecular polymerization is a key requisite for the observed effects.

We used microscopy to assess the behavior of the fuel-dependent synthetic cells with an intermediate degree of functionalization (DF = 70%, beyond which the lifetime started to increase again), and for the one with the highest lifetime (DF = 100%, Figures 4C and S14). In both cases, the initial droplet morphology was not spherical and thus completely different from that of the droplets without supramolecular polymers or those with polymers at DF = 25% (Figure 2D). They remained deformed for the initial minutes before they eventually formed spherical structures (*t* < 13 min). This apparent mechanical reinforcement is exemplified by the droplet-shaped recovery profiles upon fusion, which show slower recovery at DF = 100% compared to the control in the absence of supramolecular polymers (Figure S15). Unlike the droplets at DF = 25%, these droplets were large early in the cycle. Moreover, even later in the lifecycle (*t* > 13 min) fusion events still occurred, which we did not observe at DF = 0 or 25%. Given that at DF = 100% the supramolecular polymer becomes highly positively charged, it follows that the interaction with the polyanion in the droplet became stronger, stabilizing the droplets even at lower concentrations of the activated peptide. We therefore hypothesize that as the DF is increased, the supramolecular polymers gradually partition strongly into the coacervate droplets. To probe this hypothesis, we employed FRAP and investigated the changes in the diffusivity of the droplet components (Figures 4D, S7, and S8). In accordance with the measurements at DF = 25%, no significant changes in the peptide’s diffusivity were observed. In contrast, we found that the diffusivity of pSS more than doubled compared to the control experiments at DF = 0% and nearly tripled compared to DF = 25% (Tables S6–S9). We attribute this drastic increase in diffusivity to the disruption of the interaction network between the peptide and the pSS by the incorporation of the supramolecular polymer. The multivalent effect, enabled by the supramolecular polymerization, is likely the driving force for why the higher charge density of the activated peptide can be outcompeted by the supramolecular polymer (see supporting discussion). This result also explains why we observed fusion events by fluorescence microscopy even late in the lifecycle, as the more liquid-like viscosity can facilitate continued fusion.

Analysis of costaining experiments further supported the proposed strong partitioning of the supramolecular polymers at DF = 100% (Figures 5A and S16). Both fluorescence channels unveiled a perfect overlap under these conditions. When we quantified the droplet diameter, we found that the diameters of the BTA and the peptide channel were identical (Ø (BTA)/Ø (Peptide) = 1.001 ± 0.043), and the observed protruding fiber structure at DF = 25% was not preserved, indicating that the supramolecular polymer becomes a truly incorporated component of the droplets under these conditions. Moreover, when we determined the partitioning coefficient of BTAClick at DF = 100%, we found that nearly all BTAClick is present in the pellet with a partitioning coefficient of *K*_p_ = 7.67 × 10^5^ (Figure 5B, Table S17). This corresponds to an increase in the local concentration of BTAClick in the synthetic cells by a factor of 200, suggesting that the supramolecular polymers will remain intact in the phase-separated compartment. Notably, this partitioning coefficient was drastically higher than those of previously reported small molecules, even those with more positive charges per molecule.^21^ We attribute this to the enhanced multivalency effect induced by the supramolecular polymerization process, which promotes increasing partitioning of BTAClick with rising DF.

**Figure 5:**
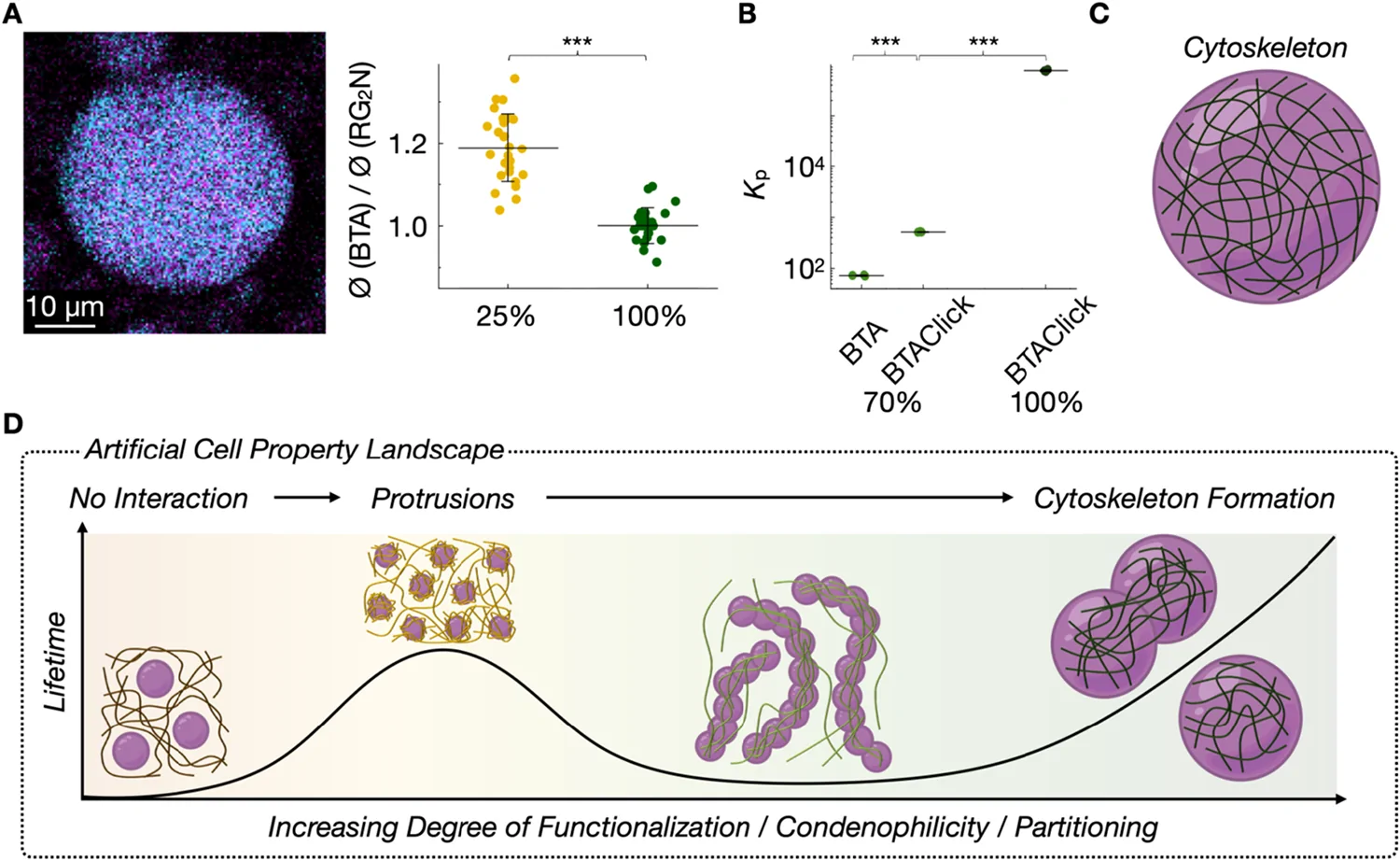
Elucidation of distinct mechanistic pathways at low and high degrees of functionalization. (A) Merged fluorescence microscopy images of droplets formed in the presence of supramolecular copolymers at DF = 100%, with the different ratios of the diameters compared to DF = 25% being shown on the right. The supramolecular polymers are stained using coumarin azide (magenta), while the droplet material is stained using NBD-functionalized RG_2_N (cyan). (B) Partitioning coefficients of BTA and BTAClick at DF = 70% and 100% based on HPLC analysis, showing the increase in partitioning with increasing DF. (C) Schematic depiction of the proposed cytoskeleton formation driven by the strong binding of BTAClick homopolymers to the pSS component of the complex coacervates. (D) Schematic depiction of the artificial cell property landscape facilitated by supramolecular polymers of varying condenophilicity.

From these studies, we conclude that the supramolecular polymers fully partitioned into the droplets, leading to an increased diffusivity of the pSS binding partner and enhanced droplet stability during starvation periods. This strong partitioning, along with the observed stabilization, is reminiscent of a rudimentary cytoskeleton, as previously reported for other synthetic cell platforms (Figure 5C).^102–106^

To finally corroborate the distinct mechanistic pathways of action at DF = 25% and 100%, we probed the sequence-specificity of the two systems (Figures S17 and S18). As previously discussed, the droplets are smaller and take a significantly longer time to settle in the presence of BTA copolymers at DF = 25%. This suggests that the interaction between the supramolecular polymer and the coacervate droplet during the nucleation and initial growth phase is crucial for the observed behavior. As expected, the characteristic increase in lifetime at DF = 25% was observed whenever Cu was added before the fuel, irrespective of the time between the addition steps. Contrastingly, when the phase separation is initiated first, i.e., EDC is added prior to Cu, this behavior collapses and the lifetime is significantly shorter (Figure S17 and Table S18). At DF = 100%, the opposite behavior was observed. Because the 100% BTAClick fibers exhibit much higher condenophilicity, their influence on the droplet lifetime persists even when CuAAC is induced after phase separation (Figure S18 and Table S19). However, when the system equilibrates for an extended period after Cu addition, the supramolecular polymers interact with pSS prior to phase separation. Consequently, the heterogeneity previously observed at DF = 100% (Figure 4C) decreases, resulting in a shorter lifetime.

## Conclusions and Outlook

In conclusion, we reported a supramolecular polymer coassembly platform that enables the construction of distinct types of cytoskeletons for active coacervate-based synthetic cells (Figure 5D). When the DF of the supramolecular polymers is 0%, they are condenophobic and consequently do not interact significantly with the synthetic cell droplets, partitioning almost quantitatively into the dilute phase. However, when functionalized, the supramolecular polymers begin to interact with synthetic cells via distinct mechanisms. When the DF increases (25%), the positively charged component of the supramolecular polymers interacts with the complex coacervates. Owing to coacervation-induced sorting within the fibers, fibers form protrusions within the droplets that extend into the surrounding medium, thereby modifying the droplets’ surface chemistry and affecting their settling and growth behavior, increasing their longevity. Moreover, by expelling condenophobic BTA building blocks and accumulating condenophilic building blocks, the synthetic cells create distinct supramolecular polymer populations within the droplets and the surrounding dilute phase. When the DF exceeds 25%, the fibers eventually partition exclusively inside the droplets (DF = 100%), forming a more traditional cytoskeleton structure. This cytoskeleton dramatically alters the droplets’ morphology and extends their lifetime beyond the level observed at 25%. Our results demonstrate that this increase in lifetime does not originate from interference with the chemical reaction cycle. Instead, the functionalized supramolecular polymer fibers can stabilize the cell’s interior even at a lower concentration of the activated peptide. We envision that this orthogonal assembly approach can be further developed to create a more diverse set of functional profiles for fuel-dependent synthetic cells. For instance, by changing the condenophobic/-philic properties of the copolymer components, an even stronger accumulation on the droplet interface may be feasible, stabilizing the cell surfaces and completely preventing fusion events (that do not resemble conventional cells found in nature or synthetic cells with stronger membrane structures), while maintaining the passive transport of small molecules. Moreover, by adjusting the exchange kinetics of the supramolecular polymer, sequence-controlled assemblies^107–109^ that can fulfill specific cellular functions, such as catalytic transformations,^110,111^ within complex coacervate-based synthetic cells may be achievable. Work along those lines is currently underway in our laboratory.

## Supplementary Material



## Notes

Materials and Methods description and additional data; synthetic protocols and compound characterization; sample preparation; UV/vis analysis, TEM microscopy; molecular control experiments; FRAP results with supplementary discussion; salt-, pH-, and solvent-dependent plate reader data; and quantification of confocal microscopy (PDF)
