# Supplementary material for: Elucidating the Reciprocal Interplay between Supramolecular Polymer Cytoskeletons and Fuel-Dependent Synthetic Cells
Source: J Am Chem Soc. 2026 Aug 12;148(33):35949–60. doi: 10.1021/jacs.6c09705 (PMC13523708; doi:10.1021/jacs.6c09705)
Supplement: Supplementary file 1 [file ja6c09705_si_001.pdf]

Supporting Information for:

Elucidating the Reciprocal Interplay Between Supramolecular Polymer Cytoskeletons and Fuel-Dependent Synthetic Cells

Authors: Nils Bäumer[1], Leonie Kauling[1], Benedikt Kirmayer[2], Simone M. Poprawa[1], Arthur Neuberger[2] and Job Boekhoven[1]

Affiliations:

[1] Department of Bioscience, School of Natural Sciences, Technical University of Munich, Lichtenbergstrasse 4, 85748 Garching, Germany.

[2] Walther-Straub-Institute of Pharmacology and Toxicology, LMU Medizin, Ludwig-Maximilians-Universität München, Goethestrasse 33, 80336 Munich, Germany.

Materials and methods

Materials. Trimesoylchloride, 1,12–dibromododecane, phthalimide potassium salt, hydrazine monohydrate, propargyl–PEG4–NHS ester, 1,12–diaminododecane and O–methylheptaethylene glycol were purchased from BLDpharm. Sodiumhydride (60 wt.% in mineral oil), trimethyl–1,3,5–benzoltricarboxylate, MES sodium salt, polystyrene sulfonate (4.3 kDa), EDC·HCl, HOBt, LiOH·H_20 silica gel, DMF, EtOH and triethylamine were purchased from Sigma Aldrich. Oxalyl chloride and 3–azido–7–hydroxycoumarin were purchased from TCI. Chloroform (anhydrous) and NaOH were purchased from Carl Roth. All chemicals and solvents were used as purchased without further purification. AcF$(RG)_2$D-OH (RG_2D) and AcF$(RG)_2$N-NH_2 (RG_2N) were purchased from CASLO ApS, as TFA salts. NBD-labelled RG_2N and RG_2D,[1] Cy3-labelled pSS (10.8 kDa)[2] and 5–(methoxycarbonyl)isophthalic acid[3] were prepared according to previously described procedures.

Methods.

Column Chromatography. Purification by column chromatography was performed in self-packed glass columns using technical-grade solvents without further purification.

Nuclear Magnetic Resonance (NMR) Spectroscopy. NMR spectra were recorded on a Bruker AV-400HD NMR spectrometer at 400 MHz (^{1}H NMR) and 101 MHz (^{13}C NMR), respectively. Chemical shifts are reported in parts per million relative to the solvent's residual signal. Spectra were analyzed and plotted using MestReNova software Version 15.1.0.

High Resolution Mass Spectroscopy (HR-MS). HR-MS was performed on a ThermoFisher Exactive Plus HCD Orbitrap Mass Spectrometer with HESI Ion source. For data acquisition and evaluation Exactive Series Tune and FreeStyle™ 1.3 software from Thermo Scientific were used.

UV-vis Spectroscopy. UV-vis measurements were performed in 96-well plates (tissue culture plates, non-treated) on a SpectraMax® ABS Plus plate reader from Molecular Devices. The absorbance at 600 nm was used as a measure for turbidity. All turbidity measurements were conducted on samples with a volume of 50 μL. The temperature for all experiments was set to 25 ºC. Full UV-vis spectra were recorded in quartz glass cuvettes (l = 1 mm) manufactured by Hellma Analytics.

Cryogenic Transmission Electron Microscopy (cryo-TEM).

BTA and **BTAAlkyne** were both dissolved in 5% EtOH and 95% Milli-Q water, diluted to 0.2–0.5 mM, and incubated at 22 °C for 18 hours. Additionally, the R 1.2/1.3 C-Flat 200-mesh copper grid was treated by glow discharge (PELCO easiGlow) at 0.19 mbar for 90 s, followed

by 70 s at 20 mA negative. The grid was mounted onto the tweezers and connected to the pole of the Vitrobot Mark IV (Thermo Fisher Scientific). The blot time was 3.5 s, the wait time 30 s, at 100% humidity and 22 °C. The grid was immediately plunge-frozen in liquid ethane and mounted onto the Gatan 626 single-tilt liquid nitrogen cryo transfer holder (Gatan). The high tension was set to 120 kV on the FEI Tecnai G2 Spirit BioTWIN microscope. Acquisition was performed with the Gatan Orius SC1000A 1024x1024 camera, and the defocus was set to -2.54 µm.

High Performance Liquid Chromatography (HPLC). HPLC measurements were performed on *ThermoFisher* Vanquish HPLC systems configured from a variable wavelength detector module (F, VF-D40-A, 190-900 nm, deuterium and tungsten lamps), quaternary and dual quaternary pump modules (C, VC-P20-A), a split sampler module (CT, VC-A12-A) and a column compartment (C, VC-C10-A). Preparative HPLC was performed on a *ThermoFisher* Hypersil GOLD 4.6 × 250 mm, 5 µm column. All HPLC analysis was performed using a gradient of H_2O: Acetonitrile (20:80 to 90:10), each with 0.1% TFA unless specifically noted otherwise.

Quantification Anhydride Hydrolysis. The hydrolysis of the RG2D anhydride was quantified using previously established methods.[4c] Samples of RG2D and RG2D in the presence of BTA polymers with a DF of 25 and 100% were prepared according to the general methods (see below, total volume: 45 µL). For the first time point (t = 0 m,in) 4.5 µL were removed right before the addition of EDC. Subsequently, EDC was added (4.5 µL) and aliquots of the mixture (5 µL) were removed at time points t = 2, 5, 10 and 15 min. To quench the EDC hydrolysis the aliquots were added to a solution of pentylamine at 250 mM in acetonitrile/MQ water (5:4, 0.1% TFA) and subsequently analyzed using HPLC (gradient of H_2O: Acetonitrile from 2:98 to 98:2). Experiments were done in triplicate.

Fluorescence Microscopy Surface Coating. To minimize wetting of the coacervate droplets on the glass surface, a coating procedure was employed. Micro-well plates (IBIDI, µ-Slide Angiogenesis Glass Bottom) were plasma cleaned for 3 minutes, followed by the addition of 50 µL of a 5 wt.% aqueous poly(vinyl)alcohol (PVA) solution. After 20 minutes, the PVA solution was removed, and the well plates were washed with MQ water. After initial drying with compressed air, the wells were dried to completion in a drying oven at 70 ºC for 30 minutes.

Confocal Fluorescence Microscopy. We used a Leica SP5 confocal microscope with a 63x oil-immersion objective to image the droplets. For the measurement, 20 µL of the sample was deposited in a PVA-coated micro-well plate. Excitation wavelengths: Coumarin azide 405 nm, NBD 488 nm, and Cy3 552 nm. For experiments with a single fluorophore, the images have been processed using the Fire LUT in ImageJ.

Partitioning Experiments. Reactions were performed according to the general sample preparation protocol described below. After EDC addition the samples were incubated for 1 minute. Following that the phase separated material was removed from the solution by centrifugation (5 minutes, 10000 rpm). 5 μL of the supernatant were added to 25 μL of 200 mM MES buffer at pH 5.3. After removal of the supernatant the pellet was dissolved in 50 μL of 5 vol.% EtOH / 200 mM MES buffer by sonication for 10 minutes. 5 μL of the solution were then added to 25 μL of 200 mM MES buffer. The droplet volume was quantified in all cases as 0.5 vol.%. The partitioning coefficient was calculated using the following equation (1), based on HPLC quantification:

$$Kp = \frac{(Area\ pellet)}{(Area\ supernatant)}\alpha \quad (1)$$

Where α corresponds to the dilution factor. Considering the droplet volume (0.5 vol.%) in the system $\alpha = 200$.

Fluorescence Recovery after Photobleaching. We used a Leica SP8 confocal microscope with a 63× water-immersion objective to analyze fluorescence recovery after photobleaching. The data analysis was carried out using Fiji following previously described procedures.[2,4]

Recovery data was background and photofading corrected using equation (2).

$$I_{corrected}(\mathrm{t}) = \frac{I_{raw}(\mathrm{t}) - I_{background}(t)}{I_{fading}(\mathrm{t}) - I_{background}(\mathrm{t})} \quad (2)$$

With $I_{corrected}$ (t): background and photofading corrected fluorescence intensity at time point t; I_{raw} (t): raw fluorescence intensity at time point t; $I_{background}$ (t): fluorescence intensity of background at time t; and I_{fading} (t): fluorescence intensity of a neighboring not bleached droplet at time point t.

The corrected intensities were normalized by the prebleach intensity using equation (3).

$$I_{normalized}(\mathrm{t}) = \frac{I_{corrected}(\mathrm{t})}{I_{prebleach}} \quad (3)$$

With $I_{normalized}$ (t): normalized fluorescence intensity at time point t; and $I_{prebleach}$: fluorescence intensity before photobleaching.

For the fit of the FRAP recovery trace equation (4) was used.[5]

$$y = a(1 - e^{t50*x}) + b \quad (4)$$

With y: $I_{normalized}$ (t); t_{50}: recovery half-time; x: time; and a and b: fitting parameters.

The diffusivity constant was calculated using equation (5).[4]

$$D = 0.224 * \frac{r^2}{t_{50}} \quad (5)$$

With D: diffusivity constant; and r: radius of the bleached spot.

Droplet Diameter Analysis. The BTA (co-polymers) were stained using coumarin azide, whereas droplet material was stained using NBD-labeled RG_2N.[1] The signal intensity of the different channels was exported using Fiji. The raw data was obtained for 9 different droplets from each condition and 3 different z layers per droplet, resulting in 27 data sets for each imaging channel and experimental condition (104 data sets in total). The raw data was smoothed with the Savitzky-Golay method across 50 and 30 data points for 25% and 100% functionalization, respectively. The diameter was determined using an intensity threshold of 15% relative to the maximum of the smoothed data. Data points were excluded when either channel's smoothed data did not cross this threshold, for instance, because another droplet was nearby. This analysis workflow and exclusion criteria yielded 27 data points for the samples at 25% functionalization and 24 data points for 100% functionalization. See Table S10 for the exact values.

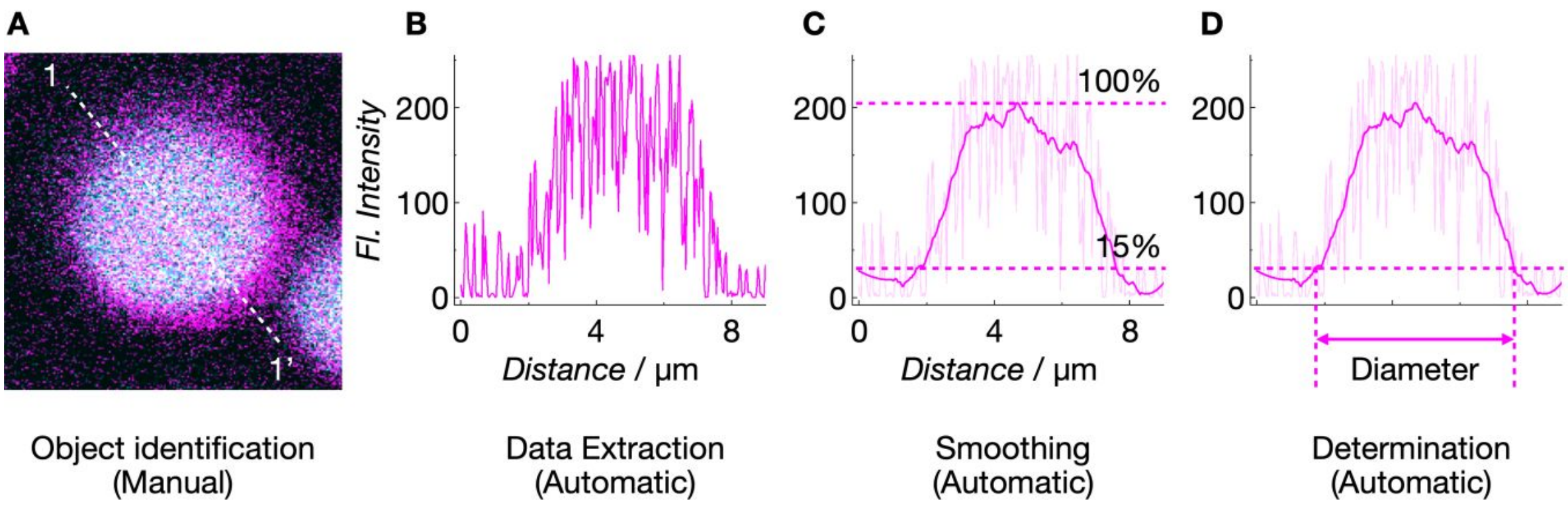

Fig. S1: a) Representative fluorescence microscopy image of co-staining experiments of droplets formed in the presence of supramolecular copolymers (DF = 25%) and the manual identification of the droplet diameter along 1-1'. b) Experimental raw data of the coumarin azide channel (magenta) along 1-1'. c) Smoothed experimental data using the Savitzky-Golay method (50 points). Dashed lines indicate the maximum of the smoothed intensity and the 15% intensity threshold. d) Diameter of the droplet determined by the intersection of the smoothed data and the 15% threshold.

Sample Preparation and General Experimental Conditions. All system components were prepared in separate stock solutions. RG_2D stock solutions were prepared in 200 mM MES buffer (pH = 5.3) at c = 100 mM; pSS (4.3 kDa) stock solutions were prepared in 200 mM MES buffer (pH = 5.3) at c = 200 mM; RG_2N stock solutions were prepared in 200 mM MES buffer (pH = 5.3) at c = 30 mM; Ascorbic Acid stock solutions were prepared in Milli-Q water at c =

100 mM and *c* = 200 mM; N_3-Arginine stock solutions were prepared in 200 mM MES buffer (pH = 5.3) at *c* = 20 mM and *c* = 40 mM; $CuSO_4$ stock solutions were prepared in Milli-Q water at *c* = 25 mM and *c* = 50 mM. Stock solutions of **BTA** and **BTAAlkyne** were prepared at *c* = 50 mM in EtOH. To ensure that the samples were molecularly dissolved the stock solutions were heated using a heat gun before use. After cooling back to room temperature, the stock solutions were diluted with 200 mM MES buffer (pH = 5.3) in a ratio of 1:10 and equilibrated at room temperature for 30 minutes prior to use. EDC stock solutions were prepared in 200 mM MES buffer (pH = 5.3) at *c* = 750 mM immediately prior to use, using a new stock solution for every experiment. All dye-labelled molecule stock solutions were prepared in Milli-Q water at *c* = 25 μM. All stock solutions other than those of **BTA** and **BTAAlkyne** were stored at *T* = 4 °C in between experiments.

All experiments on chemically fueled complex coacervation were done using the following experimental conditions: c (RG_2D) = 12.3 mM; c (pSS, 4.3 kDa) = 20.0 mM; c (EDC) = 75 mM at T = 25 °C in 5% EtOH / 200 mM MES buffer (pH = 5.3).

Experiments on passive complex coacervates (FRAP) were done using the following experimental conditions: c (RG_2D) = 4.3 mM; c (RG_2N) = 8.0 mM; c (pSS, 4.3 kDa) = 20.0 mM at T = 25 °C in 5% EtOH / 200 mM MES buffer (pH = 5.3).

All samples were prepared using the following pipetting sequence, unless specifically noted otherwise. For experiments using a dye-labelled species the specific compound was added prior to the addition of $CuSO_4$ (Coumarin-N_3) or EDC / RG_2N (NBD-RG_2D; NBD RG_2N; Cy3-pSS), respectively:

1. MES buffer
2. EtOH
3. **BTA-(co)-polymer**
4. RG_2D
5. pSS
6. Ascorbic Acid
7. N_3-Arginine
8. $CuSO_4$
9. EDC / RG_2N

CuAAC Functionalization of BTAAlkyne to BTAClick. Across all experiments on the functionalization of **BTAAlkyne** in isolation, or in the presence of the synthetic cells, the supramolecular polymers have been prepared according to the sample preparation protocol described above.

Unless explicitly stated otherwise, all experiments at DF ≤ 25% have been done using the following final concentrations in the system:

c (**BTA**) = 1 mM – (DF × 1 mM)

for instance, for DF = 25%: c (**BTA**) = 1 mM – (25% × 1 mM) = 0.75 mM

c (**BTAAlkyne**) = DF × 1 mM

for instance, for DF = 25%: c (**BTAAlkyne**) = 25% × 1 mM = 0.25 mM

c (Asc. Ac.) = 2 mM

c (N_3-Arginine) = 0.5 mM

c ($CuSO_4$) = 0.5 mM

Unless explicitly stated otherwise, all experiments at DF > 25% have been done using the following final concentrations in the system:

c (**BTA**) = 1 mM – (DF × 1 mM)

for instance, for DF = 70%: c (**BTA**) = 1 mM – (70% × 1 mM) = 0.3 mM

c (**BTAAlkyne**) = DF × 1 mM

for instance, for DF = 70%: c (**BTAAlkyne**) = 70% × 1 mM = 0.7 mM

c (Asc. Ac.) = 4 mM

c (N_3-Arginine) = 1 mM

c ($CuSO_4$) = 1 mM

Supporting Figures and Data

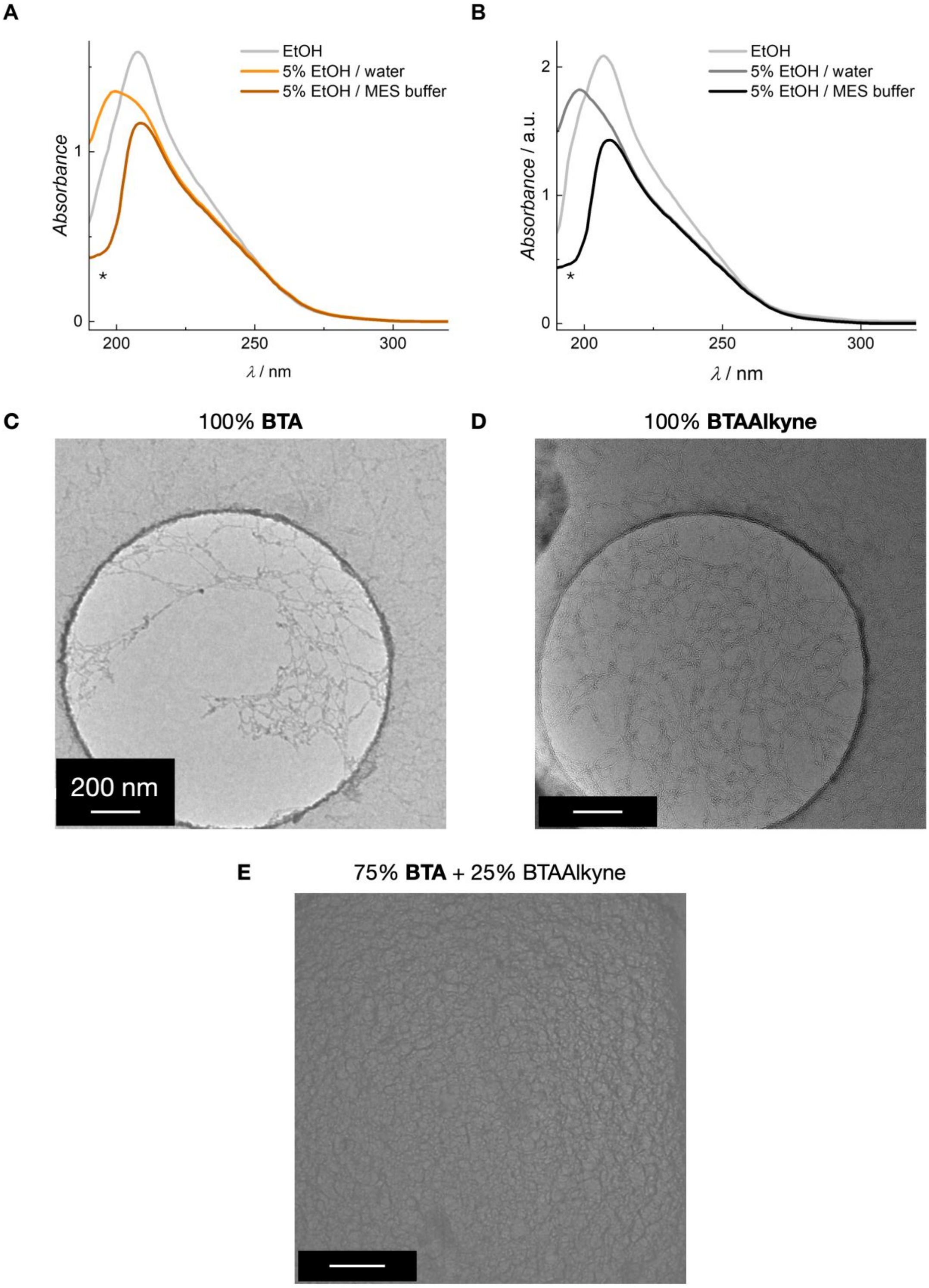

Fig. S2: a,b) Solvent-dependent UV-vis absorption spectra of **BTA** (a) and **BTAAlkyne** (b) at *c* = 0.5 mM and *T* = 25 °C. An asterisk indicates the onset of the absorption of the MES buffer. c-e) cryo-TEM micrographs of **BTA** (c), **BTAAlkyne** (d), and a copolymer containing 75% **BTA** and 25% **BTAAlkyne** after equilibration at *c* = 0.5 mM and *T* = 25 °C in a 5-vol.% EtOH aqueous solution.

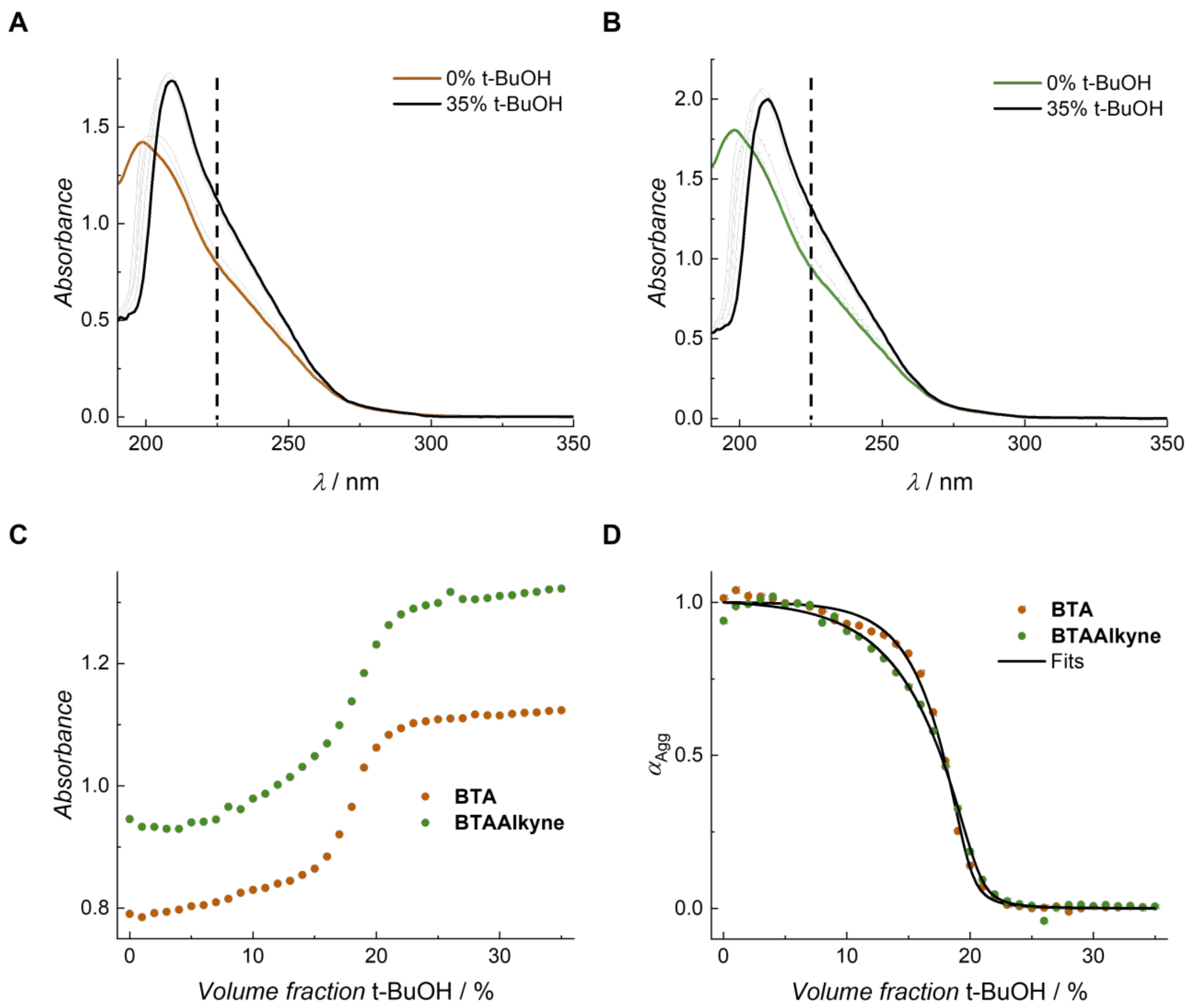

Fig. S3: a,b) Solvent-dependent UV-vis unfolding studies of **BTA** (a) and **BTAAlkyne** (b) in mixtures of water and *tert*-butanol at c = 0.05 mM and T = 25 °C. c) Changes in absorbance at λ = 225 nm (dashed lines in a and b) plotted against the volume fraction of *tert*-butanol. d) Extracted degree of polymerization (α_{Agg}) plotted against the volume fraction of *tert*-butanol. Fits were obtained using the solvent-dependent model, described by *Korevaar et al.*[6]

Table S1: Thermodynamic assembly parameter extracted from solvent-dependent studies in Fig. S3 using the solvent-dependent model. SD = standard deviation, ΔG^0 = Gibb's free energy, m = solvent dependency parameter, σ = cooperativity factor

Compound	ΔG^0 / kJ·mol^{-1} (SD)	m / kJ·mol^{-1} (SD)	σ / 10^{-3} (SD)
**BTA**	–42.1 (1.1)	90.9 (6.8)	16.9 (10.0)
**BTAAlkyne**	–36.3 (0.6)	58.5 (3.8)	6.7 (4.0)

Table S2: HPLC reaction quantification data of the CuAAC functionalization of **BTAAlkyne** to **BTAClick**, with the reaction times given in seconds. The area under the peak is given in arbitrary units. SD = standard deviation

Time	0	30	60	120	300	600
Replicate 1	61.929	1.608	1.038	1.096	1.061	1.053
Replicate 2	51.377	5.107	5.026	5.411	6.010	4.731
Replicate 3	61.426	7.325	7.313	8.178	6.651	7.579
Conversion / % (SD)	0 (10)	92.0 (4.9)	92.3 (5.5)	91.6 (6.1)	92.1 (5.3)	92.4 (5.6)

Table S3: Summary of the spin-down quantification data at DF = 0%. The area under the peak is given in arbitrary units. The partitioning coefficient was calculated using equation (1). P = Pellet; S = Supernatant; Rep. = Replicate; SD = standard deviation

**BTA:BTAClick** (Rep.)	Area **BTA** (P)	Area **BTA** (S)	K_p (SD)
100:0 (1)	0.0697	29.2579	0.4765
100:0 (2)	0.0708	28.7333	0.4928
100:0 (3)	0.0680	30.1012	0.4518
Average			0.4737 (0.0206)

Table S4: Droplet lifetime data (given in minutes) for functionalization-dependent experiments shown in Figure 2. The statistical analysis was performed using a two-tailed t-test assuming unequal variance. Rep. = Replicate

Experimental condition	Rep. 1	Rep. 2	Rep. 3	Average	Standard deviation	*p*-value
Control	11.38	11.38	12.13	11.63	0.43	–
0% **BTAClick**	13.38	12.13	13.38	12.96	0.72	0.06 (to Ctrl)
10% **BTAClick**	12.13	12.66	13.13	12.64	0.50	0.06 (to Ctrl)
20% **BTAClick**	13.38	13.63	14.11	13.71	0.37	0.003 (to Ctrl)
25% **BTAClick**	14.36	15.36	15.36	15.03	0.58	0.002 (to Ctrl)

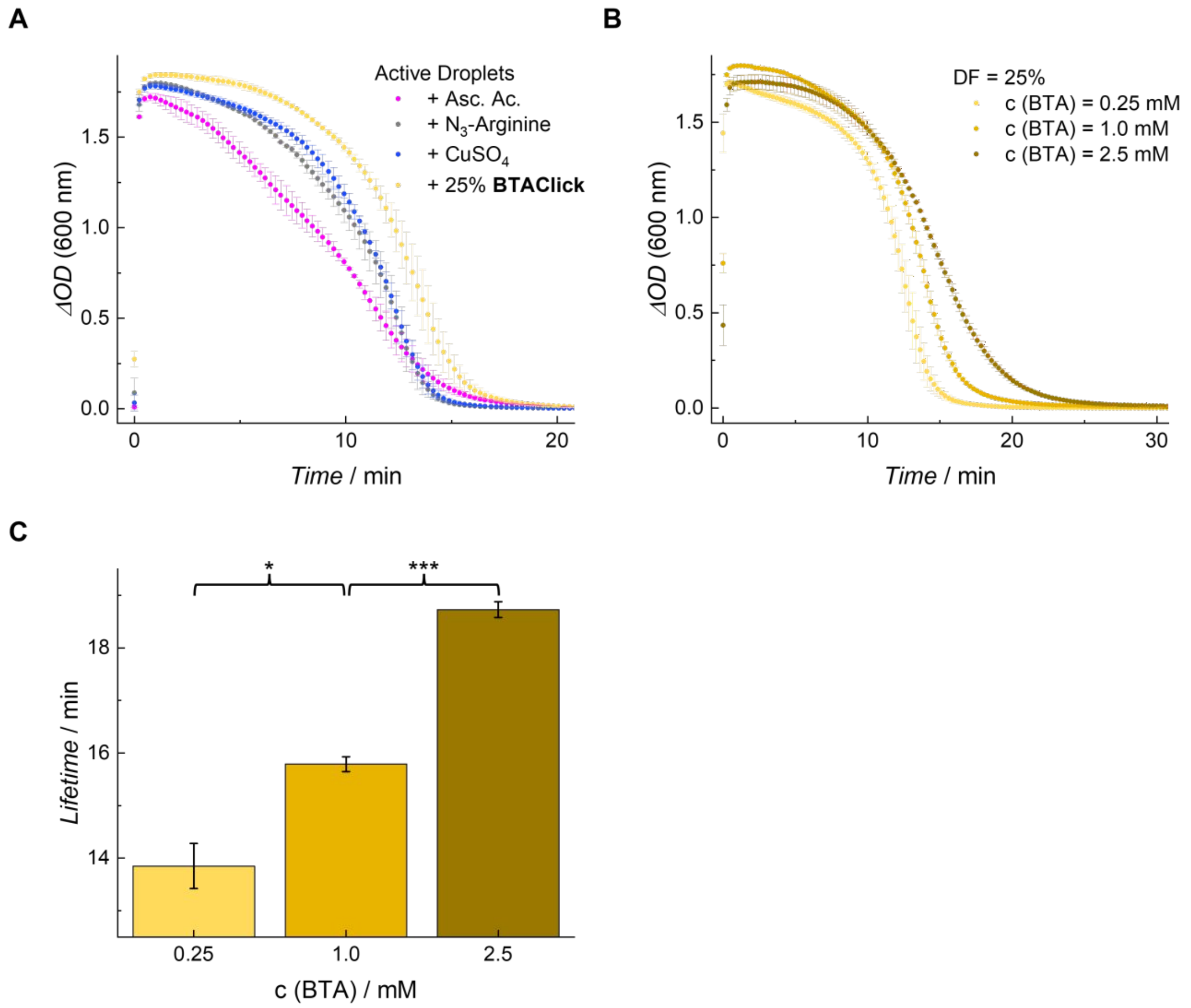

Fig. S4: a) Molecular control experiments: Differences in optical density at λ = 600 nm plotted against *t* after addition of EDC. *c* (**BTA**) = 0.75 mM; *c* (**BTAAlkyne**) = 0.25 mM with *c* (Ascorbic Acid) = 2.0 mM (pink); *c* (N_3-Arginine-N_3) = 0.5 mM (grey); *c* ($CuSO_4$) = 0.5 mM (blue); or *c* (Ascorbic Acid) = 2.0 mM, *c* (N_3-Arginine) = 0.5 mM and *c* ($CuSO_4$) = 0.5 mM (yellow) at *T* = 25 °C in 5% EtOH / 200 mM MES buffer (pH = 5.3). b) Differences in optical density at λ = 600 nm plotted against *t* after addition of EDC. *c* (**BTA**) = 0.1875 mM (light yellow), 0.75 mM (yellow) and 1.875 mM (dark yellow); *c* (**BTAAlkyne**) = 0.0625 mM (light yellow), 0.25 mM (yellow) and 0.625 mM (dark yellow) at *T* = 25 °C in 5% EtOH / 200 mM MES buffer (pH = 5.3). c) The lifetime of the droplets (defined as ΔOD < 0.25) for the different experimental conditions shown in (b). The statistical analysis was performed using a two-tailed t-test assuming unequal variance (*n* = 3 for each condition) with ns, not significant ($p > 0.05$), *$p < 0.05$, **$p < 0.01$ and ***$p < 0.001$.

The molecular control experiments in Fig. S4A show that the increase in lifetime is only observed when all components of the CuAAC are present, and no single component is responsible for the observed changes.

Table S5: Droplet lifetime data (given in minutes) for concentration-dependent experiments shown in Figure S4. The statistical analysis was performed using a two-tailed t-test assuming unequal variance. Rep. = Replicate

Experimental condition	Rep. 1	Rep. 2	Rep. 3	Average	Standard deviation	*p*-value
25% **BTAClick** (0.25 mM)	13.36	14.1	14.1	13.85	0.43	–
25% **BTAClick** (1.0 mM)	15.62	15.87	15.87	15.79	0.14	0.01 (to 0.25 mM)
25% **BTAClick** (2.5 mM)	18.56	18.82	18.82	18.73	0.15	1.7×10^{-5} (to 1.0 mM)

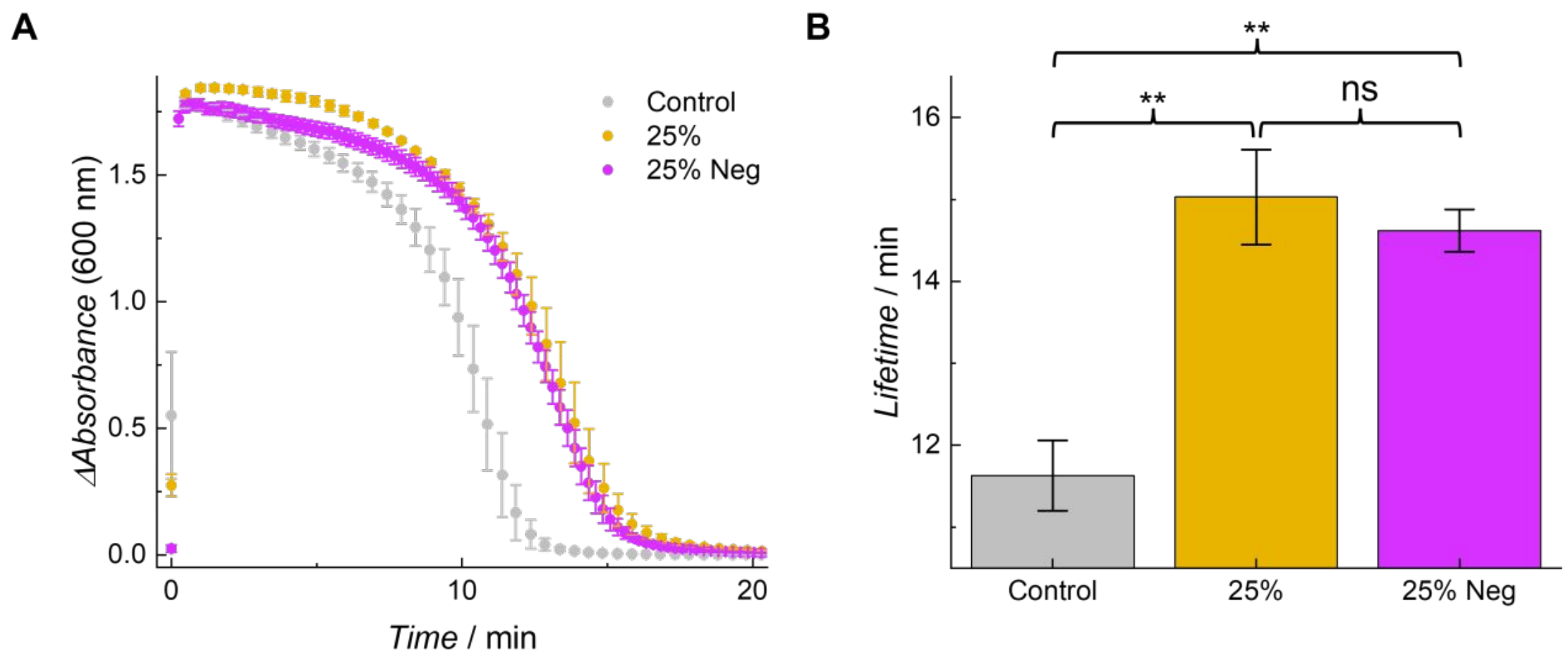

Fig. S5: a) Differences in optical density at λ = 600 nm plotted against *t* after addition of EDC. *c* (**BTA**) = 0.75 mM; *c* (**BTAAlkyne**) = 0.25 mM; *c* (Ascorbic Acid) = 2.0 mM; *c* ($CuSO_4$) = 0.5 mM with *c* (N_3-Arginine) = 0.5 mM (yellow) or *c* (Cysteic Acid-N_3) = 0.5 mM (magenta) at *T* = 25 °C in 5% EtOH / 200 mM MES buffer (pH = 5.3), with the control experiment in the absence of any supramolecular polymer or CuAAC component (grey) for comparison. b) The lifetime of the droplets (defined as ΔOD < 0.25) for the different experimental conditions shown in (a). The statistical analysis was performed using a two-tailed t-test assuming unequal variance (n = 3 for each condition) with ns, not significant (p > 0.05), *p < 0.05, **p < 0.01 and ***p < 0.001.

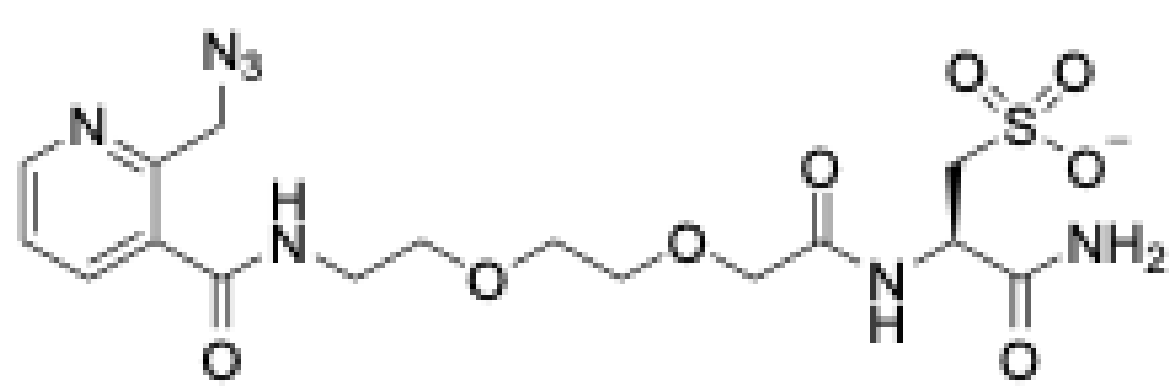

Scheme S1: Chemical structure of Cysteic Acid-N_3.

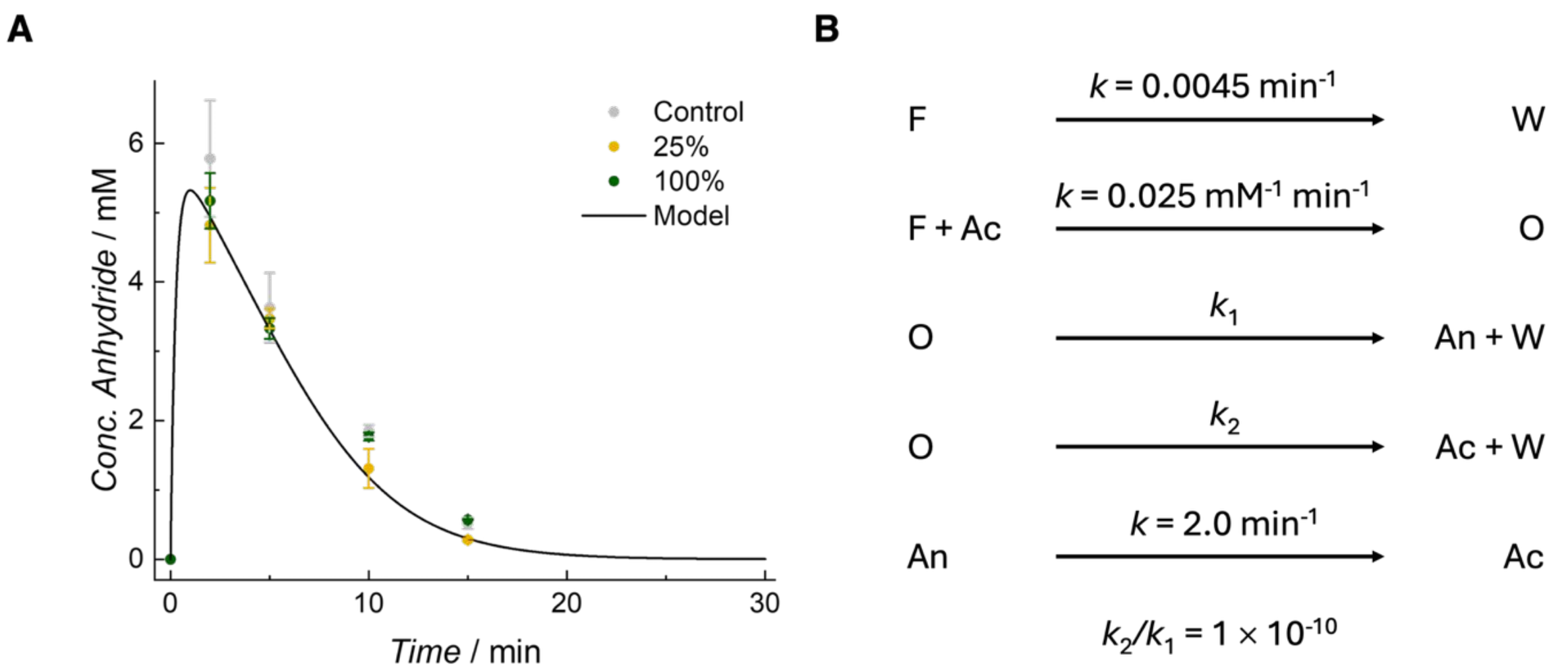

Fig. S6: a,b) Concentration of the anhydride of RG2D after EDC addition at T = 25 °C in 5% EtOH / 200 mM MES buffer (pH = 5.3) in isolation (Control, grey), in the presence of BTA fibers at DF = 25% (yellow), and in the presence of BTA fibers at DF = 100% (green), with a fitted line based on the kinetic model shown in (b), which has been reported in detail elsewhere before.[7] F = Fuel (EDC); W = Waste (EDU); Ac = unactivated peptide (free acid); O = O-acylisourea; An = activated peptide (anhydride).

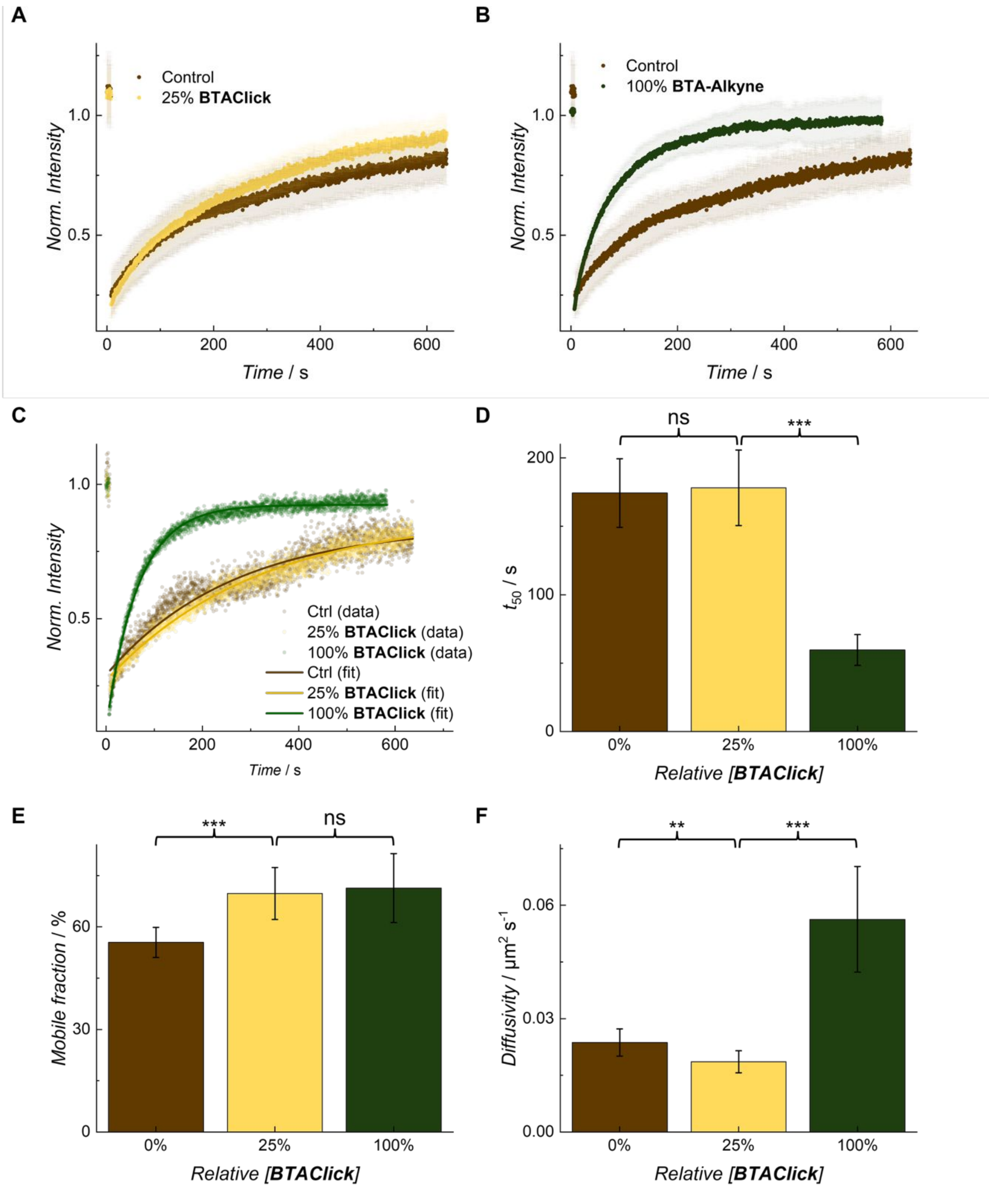

Fig. S7: a,b) FRAP intensity traces of Cy3-pSS (10.8 kDa) in the presence of homopolymers of **BTA** (Control, brown), and heteropolymers with increasing DF (DF = 25%, yellow; DF = 100%, green). c) representative examples of FRAP fits from the different experimental conditions. d-f) t_{50} (d), mobile fraction (e) and diffusivity (f) of Cy3-pSS (10.8 kDa) extracted from the intensity traces in (a) and (b). The statistical analysis was performed using a two-tailed t-test assuming unequal variance (*n* = 9 (3 technical replicates of 3 different chemical replicates) for each condition) with ns, not significant ($p > 0.05$), *$p < 0.05$, **$p < 0.01$ and ***$p < 0.001$. Experimental conditions: *c* (Cy3-pSS) = 0.5 μM; *c* (**BTA**) = 1.0 mM (Control, brown); *c* (**BTA**) = 0.75 mM; *c* (**BTAAlkyne**) = 0.25 mM; *c* (Ascorbic Acid) = 2.0 mM; *c* (N_3-Arginine) = 0.5 mM; *c* ($CuSO_4$) = 0.5 mM (25% **BTAClick**, yellow) and *c* (**BTAAlkyne**) = 1.0 mM with *c* (Ascorbic Acid) = 4.0 mM; *c* (N_3-Arginine) = 1.0 mM; *c* ($CuSO_4$) = 1.0 mM (100% **BTAAlkyne**, green) at *T* = 25 °C in 5% EtOH / 200 mM MES buffer (pH = 5.3).

Table S6: FRAP data of Cy3-pSS in the presence of homopolymers of **BTA** (top), heteropolymers of **BTA** and **BTAClick** (middle) and homopolymers of **BTAClick** (bottom) shown in Figure S7. t_{50} given in s; Mobile fraction given in % and Diffusivity given in $\mu m^2 s^{-1}$ Exp. = Experiment. X.Y (X = chemical replicate, Y = technical replicate)

Exp.	1.1	1.2	1.3	2.1	2.2	2.3	3.1	3.2	3.3
t_{50}	155.72	133.71	205.11	193.95	161.83	148.56	190.53	198.89	180.90
Mobile	58.8	59.7	46.1	57.8	55.7	52.2	54.8	60.0	54.0
Diffusivity	0.0212	0.0265	0.0209	0.0233	0.0221	0.0311	0.0256	0.0193	0.0236
Exp.	1.1	1.2	1.3	2.1	2.2	2.3	3.1	3.2	3.3
t_{50}	190.64	158.60	203.43	162.25	215.23	154.96	134.37	206.86	177.59
Mobile	65.0	74.7	63.4	80.2	74.2	60.9	76.8	73.4	59.4
Diffusivity	0.0194	0.0233	0.0172	0.0187	0.0155	0.0214	0.0188	0.0137	0.0191
Exp.	1.1	1.2	1.3	2.1	2.2	2.3	3.1	3.2	3.3
t_{50}	69.42	57.49	47.65	63.27	72.81	69.00	45.58	67.05	44.05
Mobile	94.7	63.7	59.4	65.1	73.1	69.1	75.3	69.5	72.3
Diffusivity	0.0407	0.0642	0.0728	0.0465	0.0517	0.0399	0.0621	0.0502	0.0792

Table S7: Statistical analysis of FRAP data of Cy3-pSS in Table S6. The statistical analysis was performed using a two-tailed t-test assuming unequal variance. *p*-values given in brackets with respect to the control (for 25%) and with respect to the control and 25% (for 100%).

Condition	Average t_{50}	Standard Deviation t_{50}	Average Mobile	Standard Deviation Mobile	Average Diffusivity	Standard Deviation Diffusivity
Control	174.36	25.14	55.5	4.4	0.0237	0.0036
25%	178.21<br>(0.76)	27.58	69.8<br>(3.1×10^{-4})	7.6	0.0186<br>(0.004)	0.0029
100%	59.59<br>(7.1×10^{-8}<br>1.8×10^{-7})	11.26	71.36<br>(0.001<br>0.71)	10.1	0.0564<br>(7.8×10^{-5}<br>2.9×10^{-5})	0.0140

Supplementary Discussion

By combining HPLC and FRAP analyses, we can infer a tentative mechanism underlying the interactions between the functionalized BTA (co-)polymers and the phase-separated complex coacervates. At moderate functionalization (DF = 25%), the supramolecular polymers preferentially partition into the droplet, relative to the surrounding medium. However, as the HPLC analysis (Table S10) shows, most of the BTA material remains in the surrounding medium, given the considerably larger volume of the surrounding medium relative to the droplet (a factor of 200). In isolation, the pSS is densely bound by the activated peptide, given the relatively high charge density and high interaction strength driven by combined electrostatic interactions between the oppositely charged species and additional cation-π-interactions between the arginine residues and the pSS, resulting in a relatively low mobile

fraction and diffusivity. As the BTA copolymers partition, they can partially break apart this network, increasing the mobile fraction of pSS. However, as the BTA in the copolymer is being dragged in by its co-assembled BTAClick, a species is introduced into the droplet that does not interact favorably with pSS, lowering its diffusivity. Since the amount of partitioned material is modest, so is this influence (only 19% reduction).

When the DF is increased to 100%, the homopolymers of BTAClick partition almost quantitatively into the droplet (Table S17). Moreover, at this DF, all building blocks carry a positively charged residue, thereby causing the polymers to interact strongly with the pSS. Driven by this interaction, combined with the ruptured interaction pattern between pSS and the activated peptide (high charge density of the peptide, low charge density of the supramolecular polymer) the diffusivity increases drastically. However, as the system overall is overcharged by pSS, the increase in diffusivity of the pSS does not affect the diffusivity of the peptide, which is always greater than (control and DF = 25%) or equal to (DF = 100%) that of pSS.

Table S8: FRAP data of NBD-RG$_2$N in the presence of homopolymers of **BTA** (top), heteropolymers of **BTA** and **BTAClick** (middle) and homopolymers of **BTAClick** (bottom) shown in Figure S8. t_{50} given in s; Mobile fraction given in % and Diffusivity given in $\mu m^2 s^{-1}$ Exp. = Experiment. X.Y (X = chemical replicate, Y = technical replicate)

Exp.	1.1	1.2	1.3	2.1	2.2	2.3	3.1	3.2	3.3
t_{50}	40.28	35.42	38.92	30.62	33.52	29.74	31.58	31.51	34.12
Mobile	42.2	40.9	43.4	44.6	52.7	44.8	49.2	50.8	48.4
Diffusivity	0.0874	0.0629	0.0564	0.0645	0.060	0.0687	0.0441	0.0707	0.0533
Exp.	1.1	1.2	1.3	2.1	2.2	2.3	3.1	3.2	3.3
t_{50}	32.30	29.97	29.80	29.84	35.39	31.04	29.01	32.91	31.85
Mobile	44.5	47.4	46.9	48.9	47.1	44.4	46.6	47.8	48.9
Diffusivity	0.0420	0.0618	0.0734	0.0929	0.0558	0.0703	0.0635	0.0654	0.0562
Exp.	1.1	1.2	1.3	2.1	2.2	2.3	3.1	3.2	3.3
t_{50}	34.98	20.85	42.83	17.90	16.91	48.39	28.19	30.40	26.04
Mobile	57.5	30.8	55.8	33.6	43.3	44.3	63.2	64.6	60.5
Diffusivity	0.0488	0.1251	0.0674	0.1560	0.1365	0.0412	0.0532	0.0483	0.0639

Table S9: Statistical analysis of FRAP data of NBD-RG_2N in Table S8. The statistical analysis was performed using a two-tailed t-test assuming unequal variance. *p*-values given in brackets with respect to the control (for 25%) and with respect to 25% (for 100%).

Condition	Average t_{50}	Standard Deviation t_{50}	Average Mobile	Standard Deviation Mobile	Average Diffusivity	Standard Deviation Diffusivity
Control	33.97	3.67	46.3	4.1	0.0631	0.0122
25%	31.35<br>(0.08)	2.00	46.9<br>(0.69)	1.6	0.0646<br>(0.81)	0.0140
100%	29.61<br>(0.28)	10.88	50.4<br>(0.38)	12.8	0.0823<br>(0.24)	0.0441

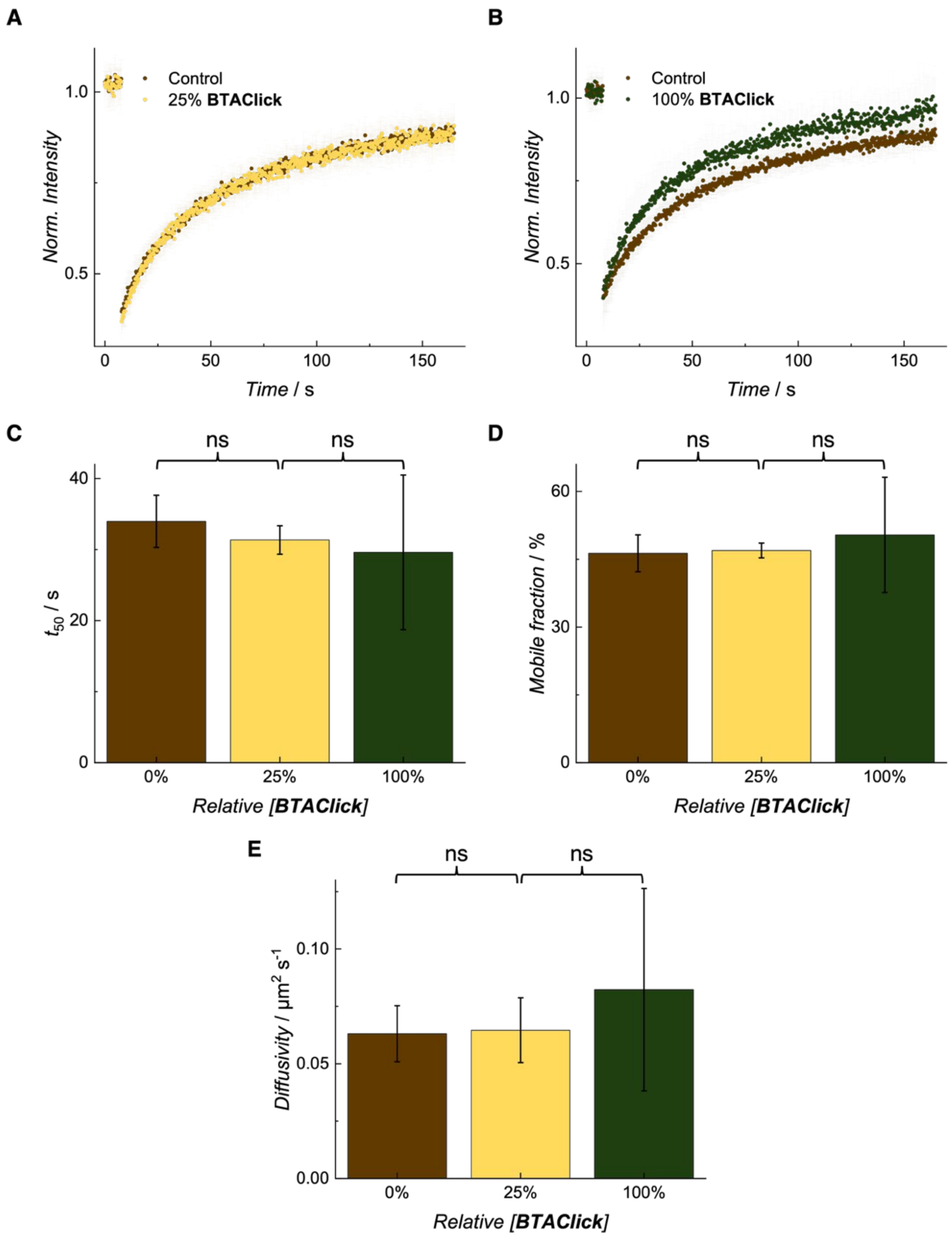

Fig. S8: a,b) FRAP intensity traces of NBD-RG$_2$N in the presence of homopolymers of **BTA** (Control, brown), and heteropolymers with increasing DF (DF = 25%, yellow; DF = 100%, green). c-e) t_{50} (c), mobile fraction (d) and diffusivity (e) of NBD-RG$_2$N extracted from the intensity traces in (a) and (b). The statistical analysis was performed using a two-tailed t-test assuming unequal variance (*n* = 9 (3 technical replicates of 3 different chemical replicates) for each condition) with ns, not significant ($p > 0.05$), *$p < 0.05$, **$p < 0.01$ and ***$p < 0.001$. Experimental conditions: *c* (NBD-RG$_2$N) = 0.5 µM; *c* (**BTA**) = 1.0 mM (Control, brown); *c* (**BTA**) = 0.75 mM; *c* (**BTAAlkyne**) = 0.25 mM; *c* (Ascorbic Acid) = 2.0 mM; *c* (N_3-Arginine) = 0.5 mM; *c* ($CuSO_4$) = 0.5 mM (25% **BTAClick**, yellow) and *c* (**BTAAlkyne**) = 1.0 mM with *c* (Ascorbic Acid) = 4.0 mM; *c* (N_3-Arginine) = 1.0 mM; *c* ($CuSO_4$) = 1.0 mM (100% **BTAAlkyne**, green) at *T* = 25 °C in 5% EtOH / 200 mM MES buffer (pH = 5.3).

Table S10: Summary of the spin-down quantification data at DF = 0% and DF = 25%. The area under the peak is given in arbitrary units. The partitioning coefficient was calculated using equation (1). The statistical analysis was performed using a two-tailed t-test assuming unequal variance. P = Pellet; S = Supernatant; Rep. = Replicate; SD = standard deviation

**BTA:BTAClick** (Rep.)	Area **BTA** (P)	Area **BTA** (S)	K_p (SD)	Area **BTA-Click** (P)	Area **BTA-Click** (S)	K_p (SD)
100:0 (1)	0.0697	29.2579	0.4765	–	–	
100:0 (2)	0.0708	28.7333	0.4928	–	–	
100:0 (3)	0.0680	30.1012	0.4518	–	–	
Average			0.4737 (0.0206)			
75:25 (1)	0.3733	17.9664	4.1556	1.3755	4.0124	68.5625
75:25 (2)	0.3565	19.0801	3.7369	1.4507	4.0805	71.1040
75:25 (3)	0.3760	19.6973	3.8178	1.5092	4.2226	71.4820
Average			3.9034 (0.2221)			70.3828 (1.5878)
p-value			0.001 (to 100:0)			1.5×10^{-4} (to **BTA** 75:25)

Table S11: Summary of the diameter analysis shown in Figure 3 and 5. Distances are given in μm. The statistical analysis was performed using a two-tailed t-test assuming unequal variance. ID = identifier; X = data quality criteria not met; SD = standard deviation

	**BTA:BTAClick** (75:25)			**BTA:BTAClick** (0:100)		
Data ID	Distance coumarin channel	Distance NBD channel	Ratio	Distance coumarin channel	Distance NBD channel	Ratio
Droplet 1 Z1	5.254	4.026	1.305	43.333	45.139	0.960
Droplet 1 Z2	5.362	4.369	1.227	X	X	
Droplet 1 Z3	5.309	4.478	1.186	X	X	
Droplet 2 Z1	5.670	4.839	1.172	34.305	36.471	0.941
Droplet 2 Z2	5.905	5.110	1.156	33.583	35.027	0.959
Droplet 2 Z3	6.320	5.489	1.151	35.388	35.388	1
Droplet 3 Z1	7.005	5.452	1.285	37.916	41.527	0.913
Droplet 3 Z2	7.005	5.561	1.260	40.443	41.888	0.966
Droplet 3 Z3	6.427	5.813	1.106	42.250	41.527	1.017
Droplet 4 Z1	5.958	5.525	1.078	38.277	38.638	0.991
Droplet 4 Z2	5.958	4.802	1.241	38.999	38.638	1.009
Droplet 4 Z3	5.813	4.622	1.258	37.555	38.638	0.972
Droplet 5 Z1	4.370	3.990	1.095	35.749	35.027	1.021
Droplet 5 Z2	4.460	4.297	1.038	35.799	32.861	1.089
Droplet 5 Z3	5.362	4.586	1.169	37.555	34.305	1.095
Droplet 6 Z1	5.308	4.658	1.140	36.111	35.027	1.031
Droplet 6 Z2	5.507	4.622	1.191	X	X	
Droplet 6 Z3	5.327	3.918	1.360	36.833	35.749	1.030
Droplet 7 Z1	5.598	4.442	1.260	40.805	42.249	0.966
Droplet 7 Z2	5.886	4.839	1.216	40.805	41.527	0.983
Droplet 7 Z3	5.958	5.272	1.130	41.888	41.527	1.009
Droplet 8 Z1	5.380	5.055	1.064	46.944	45.499	1.032
Droplet 8 Z2	5.597	4.983	1.123	43.694	43.694	1
Droplet 8 Z3	5.669	4.658	1.217	42.610	43.333	0.983
Droplet 9 Z1	5.092	3.900	1.306	38.277	38.277	1
Droplet 9 Z2	5.236	4.189	1.250	37.554	37.554	1
Droplet 9 Z3	5.019	4.478	1.121	38.638	36.471	1.059
Average Ratio (SD)	1.189 (0.082)			1.001 (0.043)		
p-value	5.2×10^{-13}					

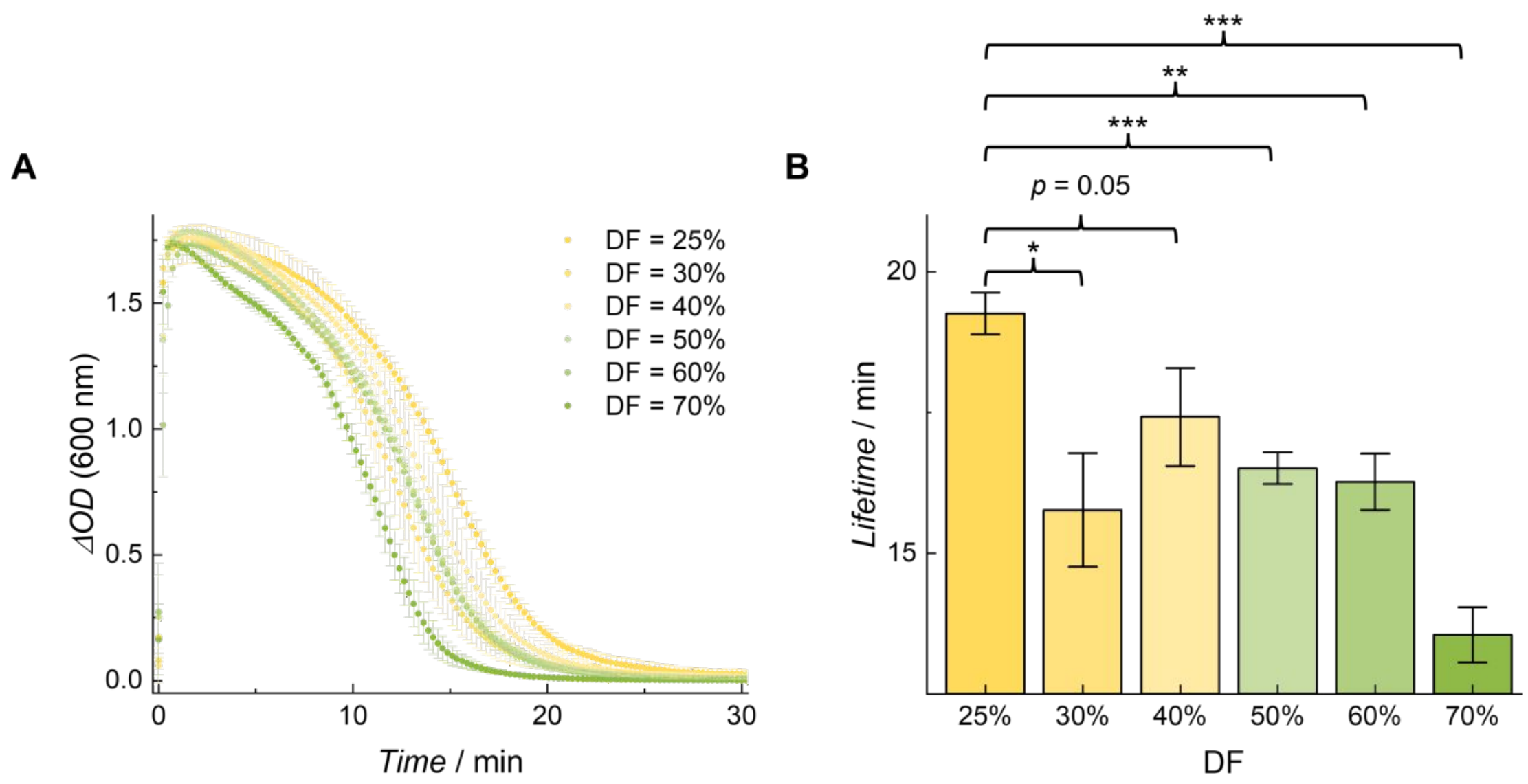

Fig. S9: a) Differences in optical density at λ = 600 nm plotted against t after addition of EDC, depending on DF at intermediate ranges (25% to 70%), where the concentration of all CuAAC components is kept constant (c (Ascorbic Acid) = 4.0 mM; c (N_3-Arginine) = 1.0 mM; c ($CuSO_4$) = 1.0 mM). b) The lifetime of the droplets (defined as ΔOD < 0.25) for the different experimental conditions shown in (a). The statistical analysis was performed using a two-tailed t-test assuming unequal variance (n = 3 for each condition) with ns, not significant (p > 0.05), *p < 0.05, **p < 0.01 and ***p < 0.001.

Table S12: Droplet lifetime data (given in minutes) for functionalization-dependent data shown in Figure S9. The statistical analysis was performed using a two-tailed t-test assuming unequal variance. Rep. = Replicate

Experimental condition	Rep. 1	Rep. 2	Rep. 3	Average	Standard deviation	*p*-value
25% **BTAClick**	18.85	19.35	19.58	19.26	0.37	–
30% **BTAClick**	14.60	16.35	16.35	15.77	1.01	0.017
40% **BTAClick**	16.60	17.32	18.33	17.42	0.87	0.050
50% **BTAClick**	16.35	16.35	16.83	16.51	0.28	7.6×10^{-4}
60% **BTAClick**	15.87	16.10	16.83	16.27	0.50	0.016
70% **BTAClick**	13.15	13.40	14.10	13.55	0.49	1.4×10^{-4}

At intermediate DF (25% to 70%), droplet lifetime decreases (Fig. S9). Moreover, the initial plateau region observed at low DF (<25%) vanishes and instead the characteristic immediate decrease observed at high DF (100%) becomes apparent. We attribute this behavior to the transition between the two main mechanisms described in the manuscript, in which the increased partitioning of BTA, driven by copolymerization with BTAClick, becomes detrimental to droplet stability. This is also evident by the fact that at these intermediate DF the influence of the supramolecular polymer cannot be differentiated from the effect of the individual components of the CuAAC reaction (Fig. S10A).

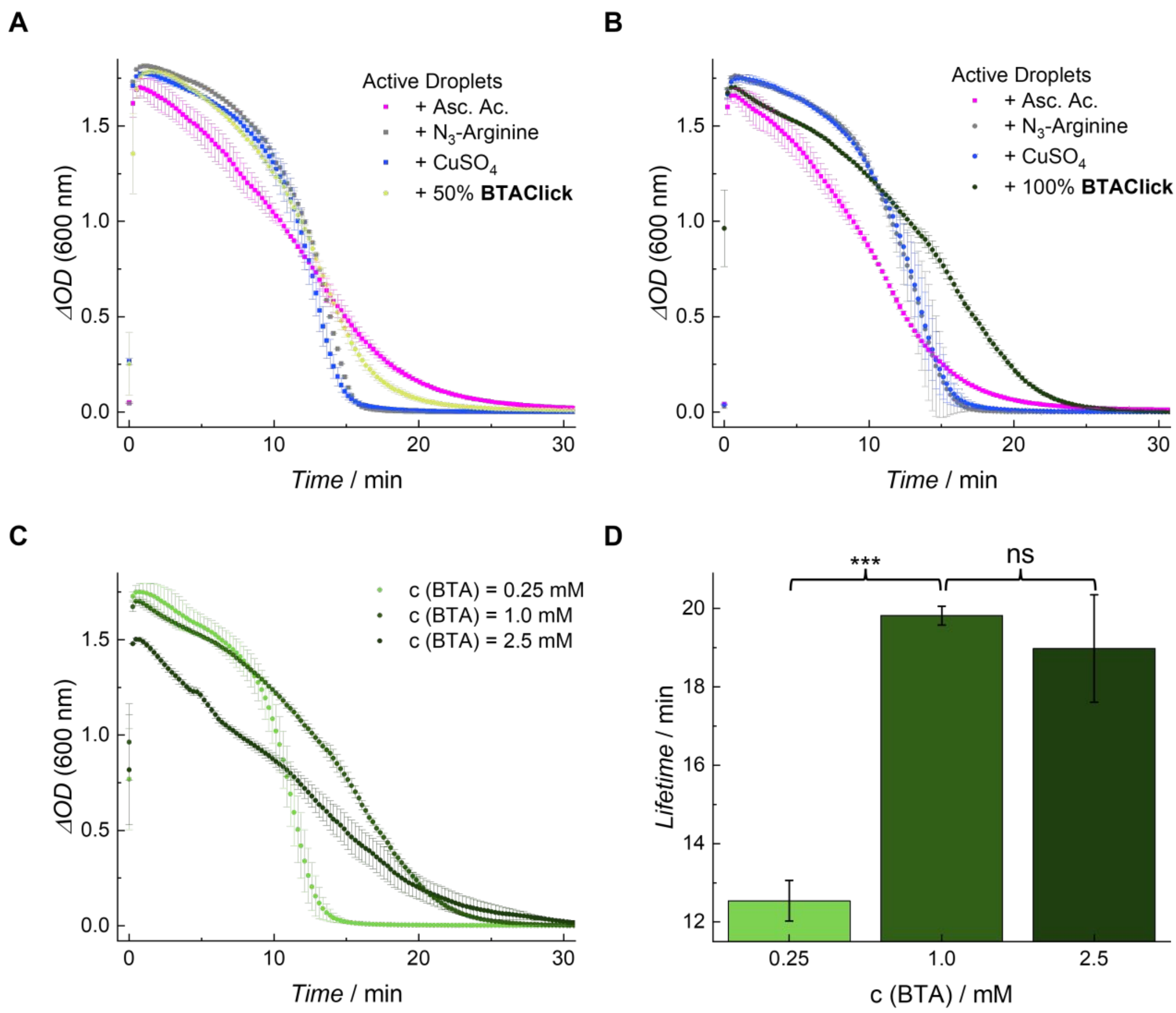

Fig. S10: a) Molecular control experiments: Differences in optical density at λ = 600 nm plotted against *t* after addition of EDC. *c* (**BTA**) = 0.5 mM; *c* (**BTAAlkyne**) = 0.5 mM with *c* (Ascorbic Acid) = 4.0 mM (pink); *c* (N_3-Arginine) = 1.0 mM (grey); *c* ($CuSO_4$) = 1.0 mM (blue); or *c* (Ascorbic Acid) = 4.0 mM, *c* (N_3-Arginine) = 1.0 mM and *c* ($CuSO_4$) = 1.0 mM (light green) at *T* = 25 °C in 5% EtOH / 200 mM MES buffer (pH = 5.3). b) Molecular control experiments: Differences in optical density at λ = 600 nm plotted against *t* after addition of EDC. *c* (**BTAAlkyne**) = 1.0 mM with *c* (Ascorbic Acid) = 4.0 mM (pink); *c* (N_3-Arginine) = 1.0 mM (grey); *c* ($CuSO_4$) = 1.0 mM (blue); or *c* (Ascorbic Acid) = 4.0 mM, *c* (N_3-Arginine) = 1.0 mM and *c* ($CuSO_4$) = 1.0 mM (dark green) at *T* = 25 °C in 5% EtOH / 200 mM MES buffer (pH = 5.3). c) Differences in optical density at λ = 600 nm plotted against *t* after addition of EDC. *c* (**BTAAlkyne**) = 0.25 mM (light green), 1.0 mM (green) and 2.5 mM (dark green) at *T* = 25 °C in 5% EtOH / 200 mM MES buffer (pH = 5.3). d) The lifetime of the droplets (defined as ΔOD < 0.25) for the different experimental conditions shown in (c). The statistical analysis was performed using a two-tailed t-test assuming unequal variance (*n* = 3 for each condition) with ns, not significant ($p > 0.05$), *$p < 0.05$, **$p < 0.01$ and ***$p < 0.001$.

The molecular control experiments show that at intermediate DF (50% as a representative example, Fig. S10A) the influence of the functionalized polymer cannot be differentiated from the control of the individual components. The increase in lifetime exceeds the sum of its parts only at DF > 70% (100% as a representative example, Fig. S10B).

Table S13: Droplet lifetime data (given in minutes) for functionalization-dependent and concentration-dependent data shown in Figure 4 and Figure S10. The statistical analysis was performed using a two-tailed t-test assuming unequal variance. Rep. = Replicate

Experimental condition	Rep. 1	Rep. 2	Rep. 3	Average	Standard deviation	*p*-value
Control	11.38	11.38	12.13	11.63	0.43	–
70% **BTAClick**	13.15	13.4	14.11	13.55	0.50	0.008
80% **BTAClick**	14.88	15.61	15.36	15.28	0.37	0.01 (to 70%)
90% **BTAClick**	17.85	17.6	18.08	17.84	0.24	0.001 (to 70%)
100% **BTAClick**	20.08	19.83	19.58	19.83	0.25	3.3×10^{-4} (to 70%)
100% **BTAClick** (0.25 mM)	12.37	12.12	13.12	12.54	0.52	–
100% **BTAClick** (1.0 mM)	20.05	19.58	19.83	19.82	0.24	3.3×10^{-4} (to 0.25 mM)
100% **BTAClick** (2.5 mM)	20.55	18.32	18.07	18.98	1.37	0.40 (to 1.0 mM)

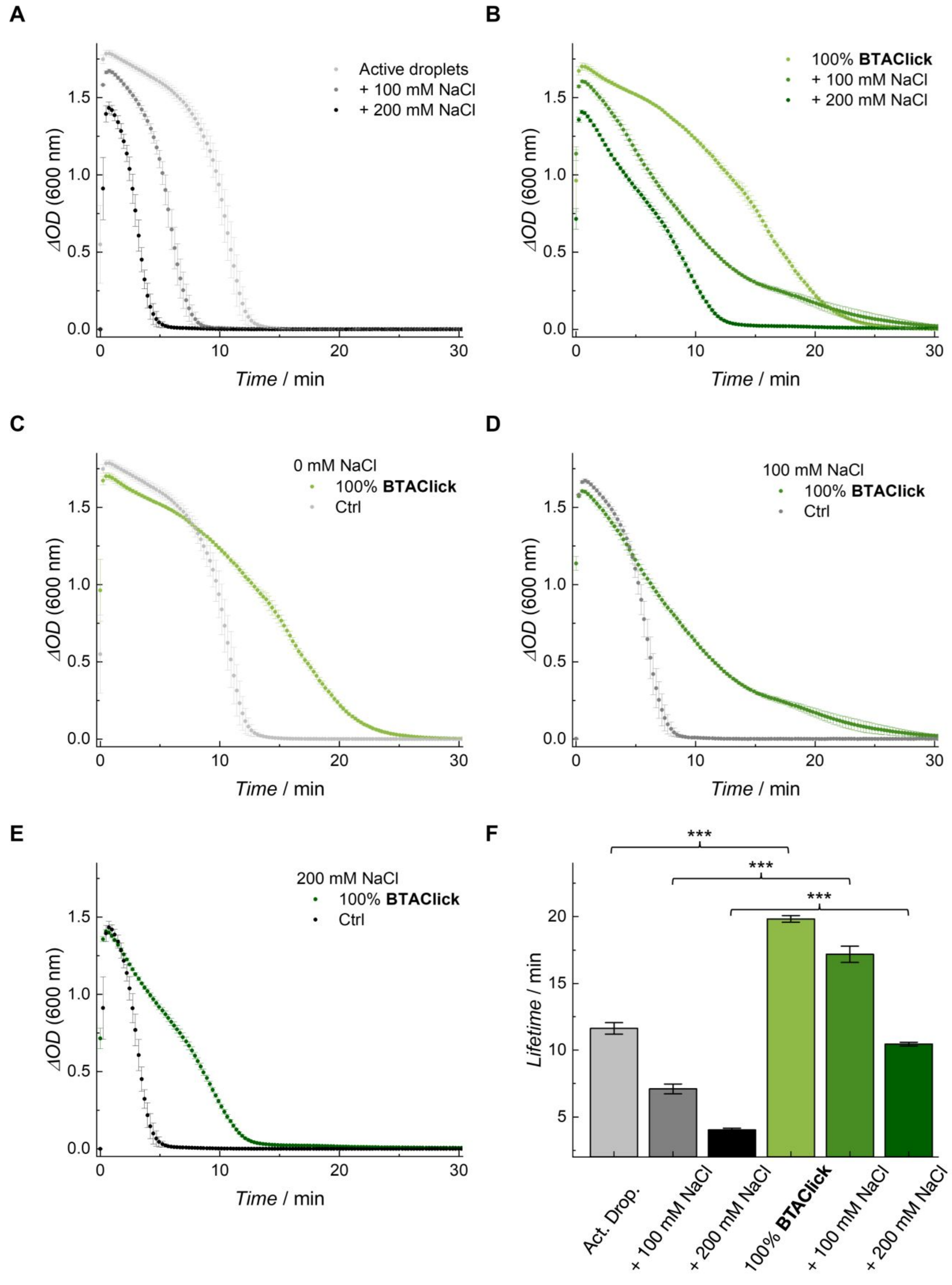

Fig. S11: a-e) Differences in optical density at λ = 600 nm plotted against t after addition of EDC. c (**BTAAlkyne**) = 1.0 mM with c (Ascorbic Acid) = 4.0 mM; c (N_3-Arginine) = 1.0 mM; c ($CuSO_4$) = 1.0 mM, with different salt concentrations. f) Changes in lifetime based on the addition of salt extracted from experiments shown in (a-e). The statistical analysis was performed using a two-tailed t-test assuming unequal variance (n = 3 for each condition) with ns, not significant ($p > 0.05$), *$p < 0.05$, **$p < 0.01$ and ***$p < 0.001$.

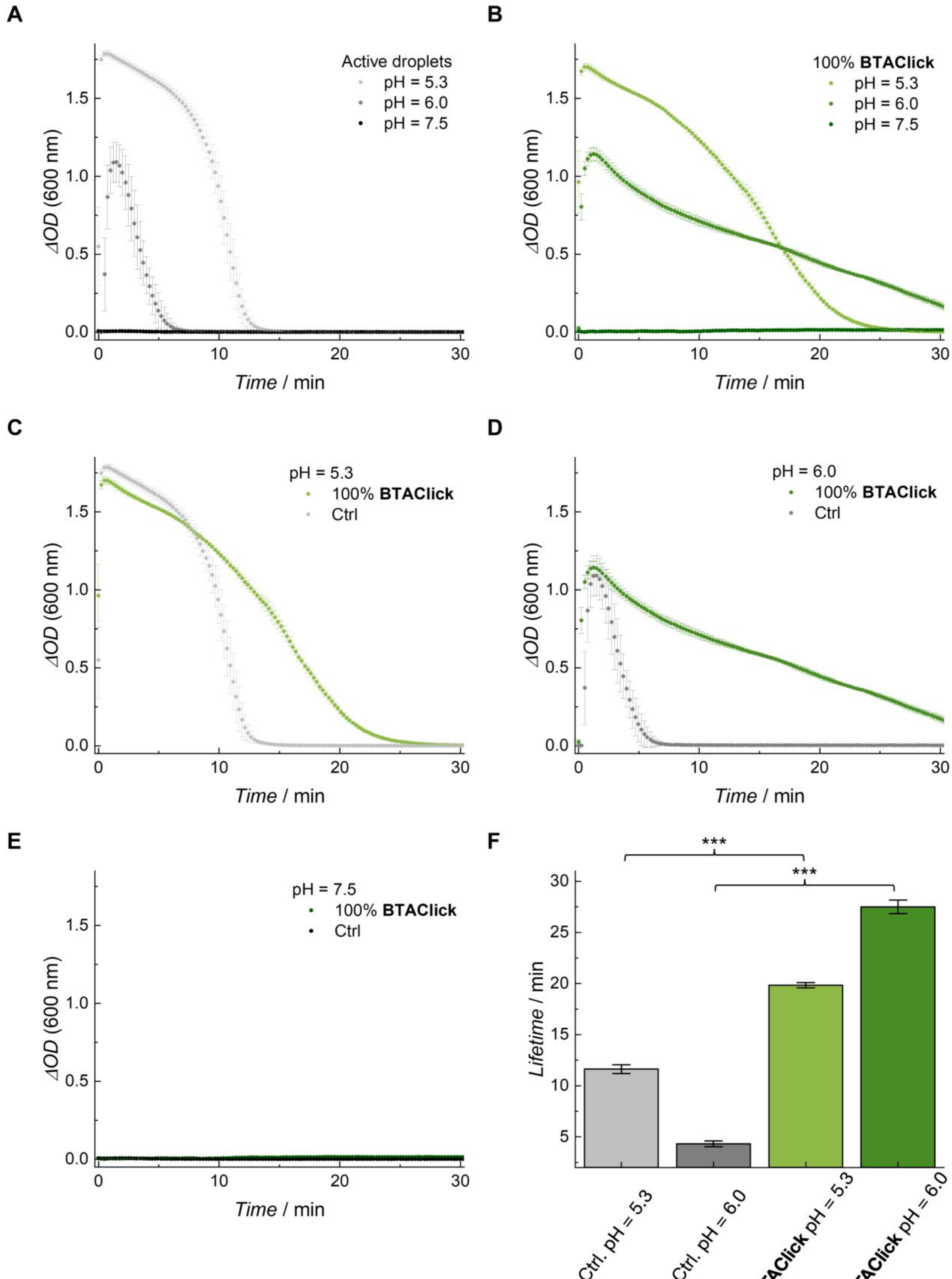

Fig. S12: a-e) Differences in optical density at λ = 600 nm plotted against t after addition of EDC. c (**BTAAlkyne**) = 1.0 mM with c (Ascorbic Acid) = 4.0 mM; c (N_3-Arginine) = 1.0 mM; c ($CuSO_4$) = 1.0 mM, with different pH. f) Changes in lifetime based on the addition of salt extracted from experiments shown in (a-d). The statistical analysis was performed using a two-tailed t-test assuming unequal variance (n = 3 for each condition) with ns, not significant ($p > 0.05$), *$p < 0.05$, **$p < 0.01$ and ***$p < 0.001$.

Table S14: Droplet lifetime data (given in minutes) for salt-dependent data shown in Figure S11. The statistical analysis was performed using a two-tailed t-test assuming unequal variance. Rep. = Replicate

Experimental condition	Rep. 1	Rep. 2	Rep. 3	Average	Standard deviation	*p*-value
Control 0 mM NaCl	11.38	11.38	12.13	11.63	0.43	–
Control 100 mM NaCl	6.70	7.18	7.42	7.10	0.37	–
Control 200 mM NaCl	3.95	4.18	3.95	4.03	0.13	–
100% **BTAClick** 0 mM NaCl	20.08	19.83	19.58	19.83	0.25	5.9×10^{-5} (to Ctrl)
100% **BTAClick** 100 mM NaCl	17.10	17.83	16.62	17.18	0.61	7.8×10^{-5} (to Ctrl)
100% **BTAClick** 200 mM NaCl	10.38	10.62	10.38	10.46	0.14	5.4×10^{-7} (to Ctrl)

Table S15: Droplet lifetime data (given in minutes) for pH-dependent data shown in Figure S12. The statistical analysis was performed using a two-tailed t-test assuming unequal variance. Rep. = Replicate

Experimental condition	Rep. 1	Rep. 2	Rep. 3	Average	Standard deviation	*p*-value
Control pH 5.3	11.38	11.38	12.13	11.63	0.43	–
Control pH 6.0	3.98	4.47	4.47	4.31	0.28	–
100% **BTAClick** pH 5.3	20.08	19.83	19.58	19.83	0.25	5.9×10^{-5} (to Ctrl)
100% **BTAClick** pH 6.0	27.25	28.25	27.0	27.5	0.66	3.2×10^{-5} (to Ctrl)

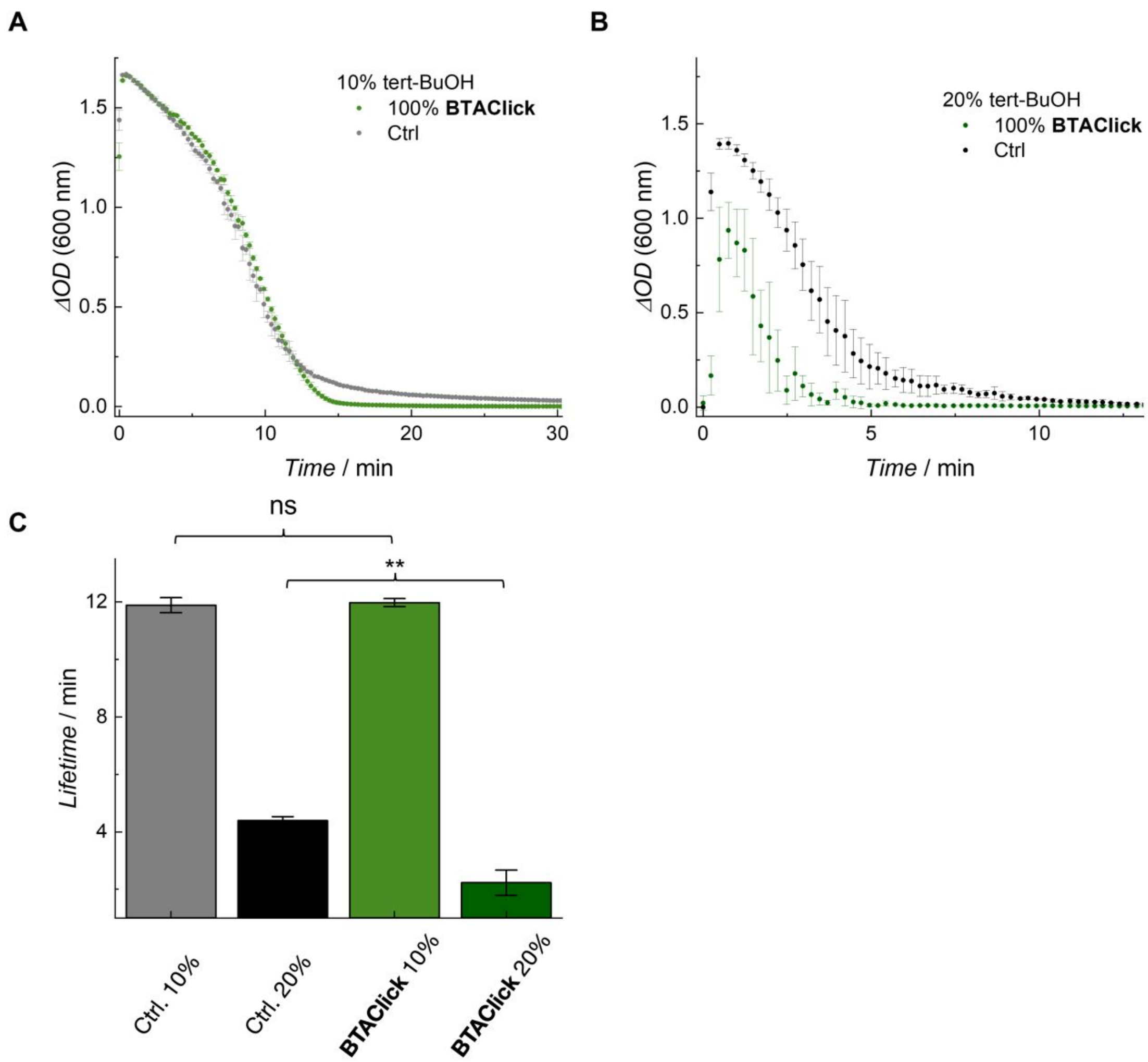

Fig. S13: a,b) Differences in optical density at λ = 600 nm plotted against t after addition of EDC. c (**BTAAlkyne**) = 1.0 mM with c (Ascorbic Acid) = 4.0 mM; c (N_3-Arginine) = 1.0 mM; c ($CuSO_4$) = 1.0 mM, with different amounts of added *tert*-butanol. c) Changes in lifetime based on the addition of salt extracted from experiments shown in (a and b). The statistical analysis was performed using a two-tailed t-test assuming unequal variance (n = 3 for each condition) with ns, not significant ($p > 0.05$), *$p < 0.05$, **$p < 0.01$ and ***$p < 0.001$.

Table S16: Droplet lifetime data (given in minutes) for solvent-dependent data shown in Figure S13. The statistical analysis was performed using a two-tailed t-test assuming unequal variance. Rep. = Replicate

Experimental condition	Rep. 1	Rep. 2	Rep. 3	Average	Standard deviation	*p*-value
Control 10%	12.15	11.63	11.90	11.89	0.26	–
Control 20%	4.22	4.47	4.47	4.39	0.14	–
100% **BTAClick** 10%	12.15	11.90	11.90	11.98	0.14	0.64 (to Ctrl)
100% **BTAClick** 20%	2.48	1.72	2.48	2.23	0.44	8.2×10^{-3} (to Ctrl)

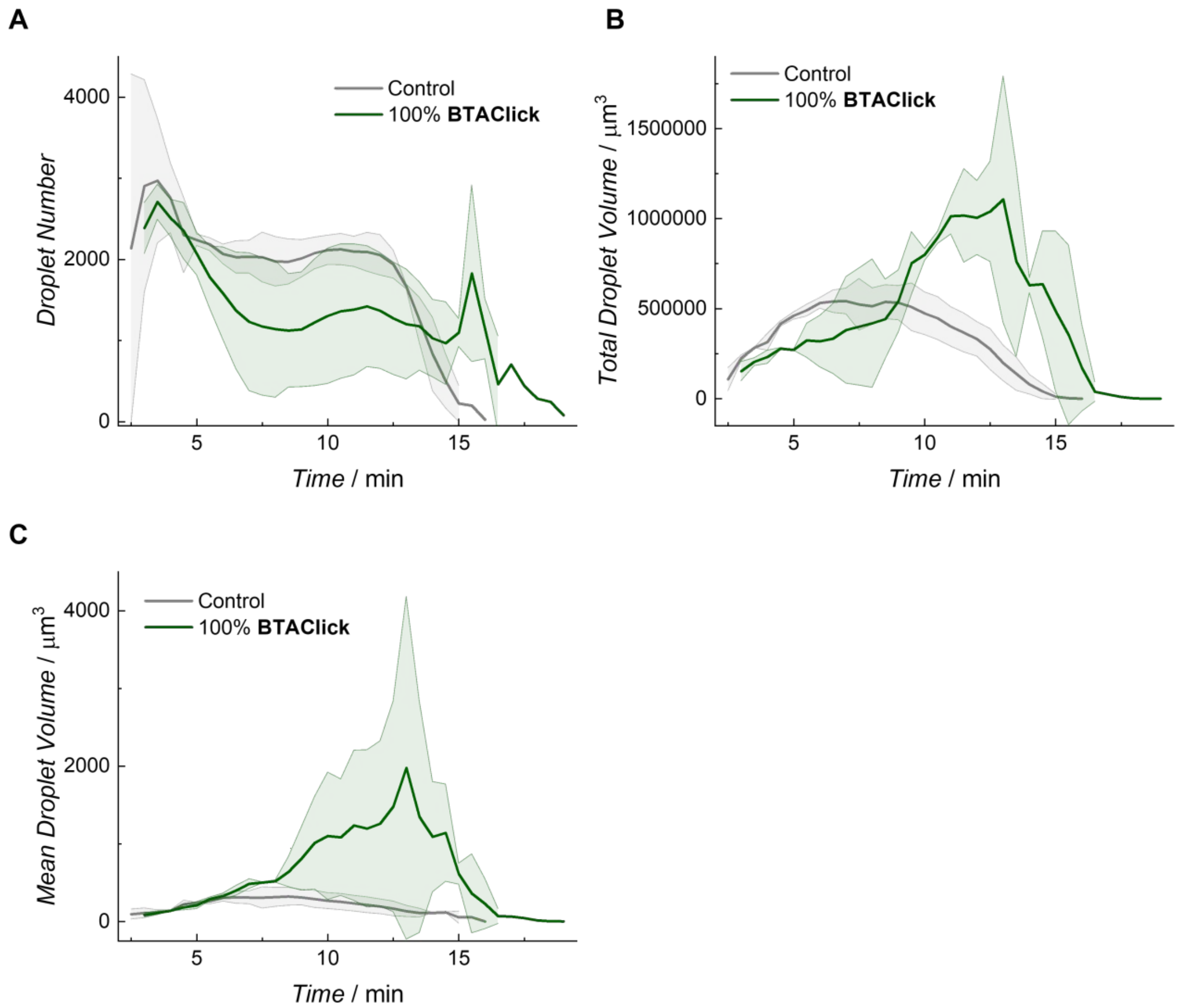

Fig. S14: a-c) Quantification of droplet number (a), total droplet Volume (b), and mean droplet volume (c) based on fluorescence microscopy under identical experimental conditions as those used to obtain representative images in Figs. 2D and 4C. The spike at around 15 minutes in panel (a) is due to vacuolization artifacts.

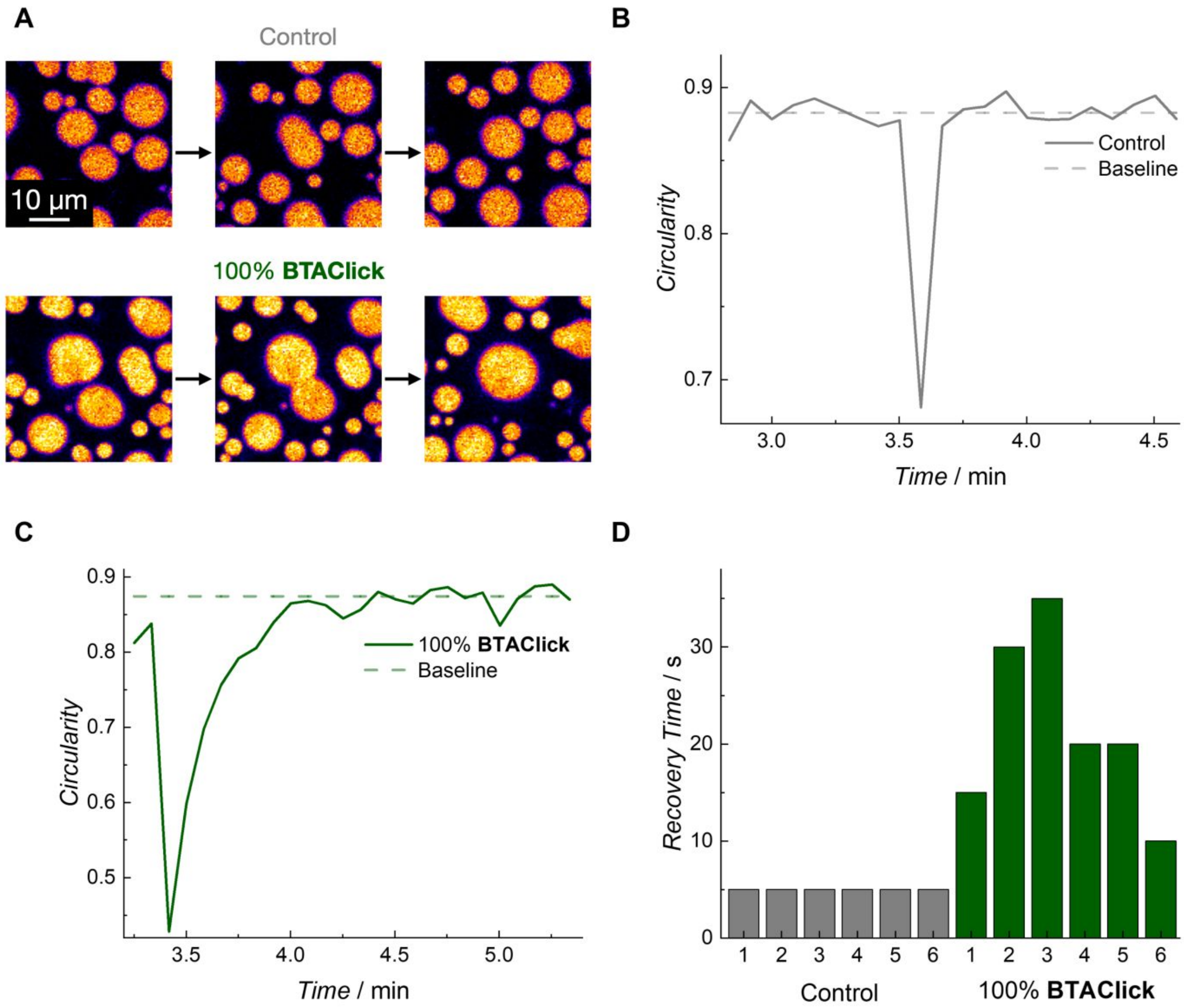

Fig. S15: a) Representative confocal microscopy images showing fusion events in the absence (top) and presence (bottom) of supramolecular polymers at DF = 100%. b,c) Representative droplet-shape recovery profiles upon fusion in the absence (b) and presence (c) of supramolecular polymers at DF = 100%. Extracted shape recovery times from 6 different fusion events recorded from two different chemical replicates (3 fusions from each sample), showing the delayed recovery in the presence of supramolecular polymers at DF = 100%. Note that the minimum possible recovery time under the imaging conditions employed is t = 5 s.

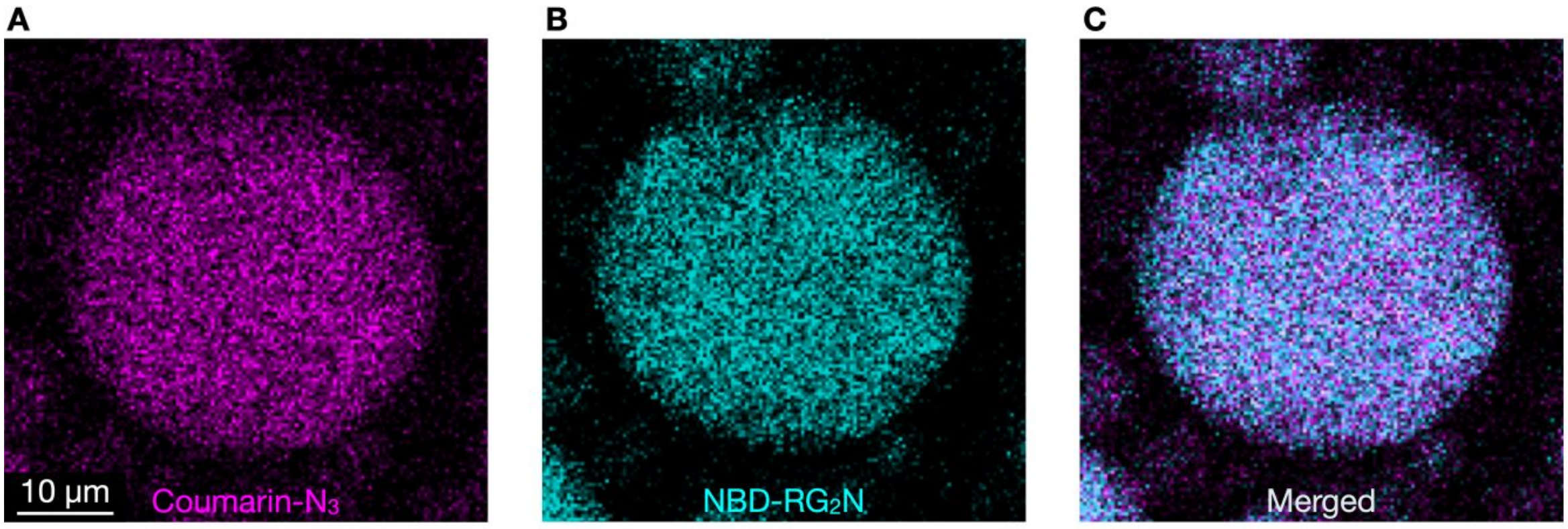

Fig. S16: a-c) Fluorescence microscopy images of droplets formed in the presence of supramolecular copolymers at DF = 100%. The supramolecular polymers are stained using coumarin azide (magenta, a), while the droplet material is stained using NBD-functionalized RG_2N (cyan, b). The merged image (c) has been reproduced from Fig. 5A for visual comparison.

Table S17: Summary of the spin-down quantification at DF = 70 and 100%. The area under the peak is given in arbitrary units. The partitioning coefficient was calculated using equation (1). The statistical analysis was performed using a two-tailed t-test assuming unequal variance. P = Pellet; S = Supernatant; Rep. = Replicate; SD = standard deviation

**BTA:BTAClick** (Rep.)	Area **BTA** (P)	Area **BTA** (S)	K_p (SD)	Area **BTA-Click** (P)	Area **BTA-Click** (S)	K_p (SD)
30:70 (1)	2.1401	5.8994	72.5531	15.0121	5.8923	509.550
30:70 (2)	2.1435	5.7802	74.1670	15.1514	5.9039	513.268
30:70 (3)	2.1038	5.9873	70.2754	15.1867	5.7854	525.001
Average			72.3318 (1.9552)			515.939 (8.065)
0:100 (1)	–	–		33.6761	0.0091	740134.1
0:100 (2)	–	–		35.3083	0.0088	802461.4
0:100 (3)	–	–		36.0247	0.0095	758414.7
Average						767003.4 (32038.987)
p-value			4.7×10^{-5} (**BTA** to **BTAClick** 30:70)			5.8×10^{-4} (**BTAClick** to **BTAClick** 30:70)

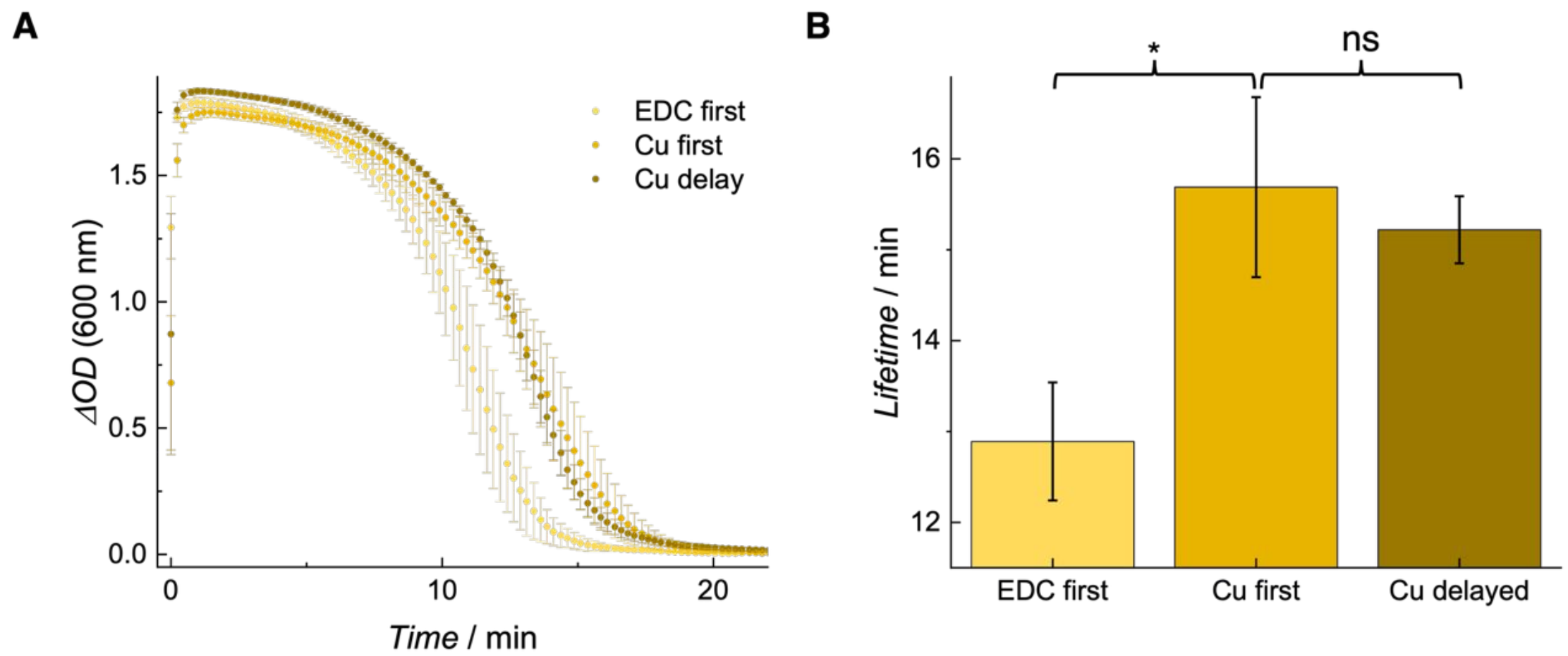

Fig. S17: a) Differences in optical density at λ = 600 nm plotted against t after addition of $CuSO_4$ (light yellow) or EDC (yellow and dark yellow). c (**BTA**) = 0.75 mM; c (**BTAAlkyne**) = 0.25 mM at T = 25 °C in 5% EtOH / 200 mM MES buffer (pH = 5.3). All parts were added sequentially with $CuSO_4$ last following 1 min of incubation after EDC addition (EDC first, light yellow); EDC last following 1 min of incubation after $CuSO_4$ addition (Cu first, yellow) and EDC last following 10 min of incubation after $CuSO_4$ addition (Cu delay, dark yellow) b) The lifetime of the droplets (defined as $\Delta OD < 0.25$) for the different experimental conditions shown in (a). The statistical analysis was performed using a two-tailed t-test assuming unequal variance (n = 3 for each condition) with ns, not significant ($p > 0.05$), *$p < 0.05$, **$p < 0.01$ and ***$p < 0.001$.

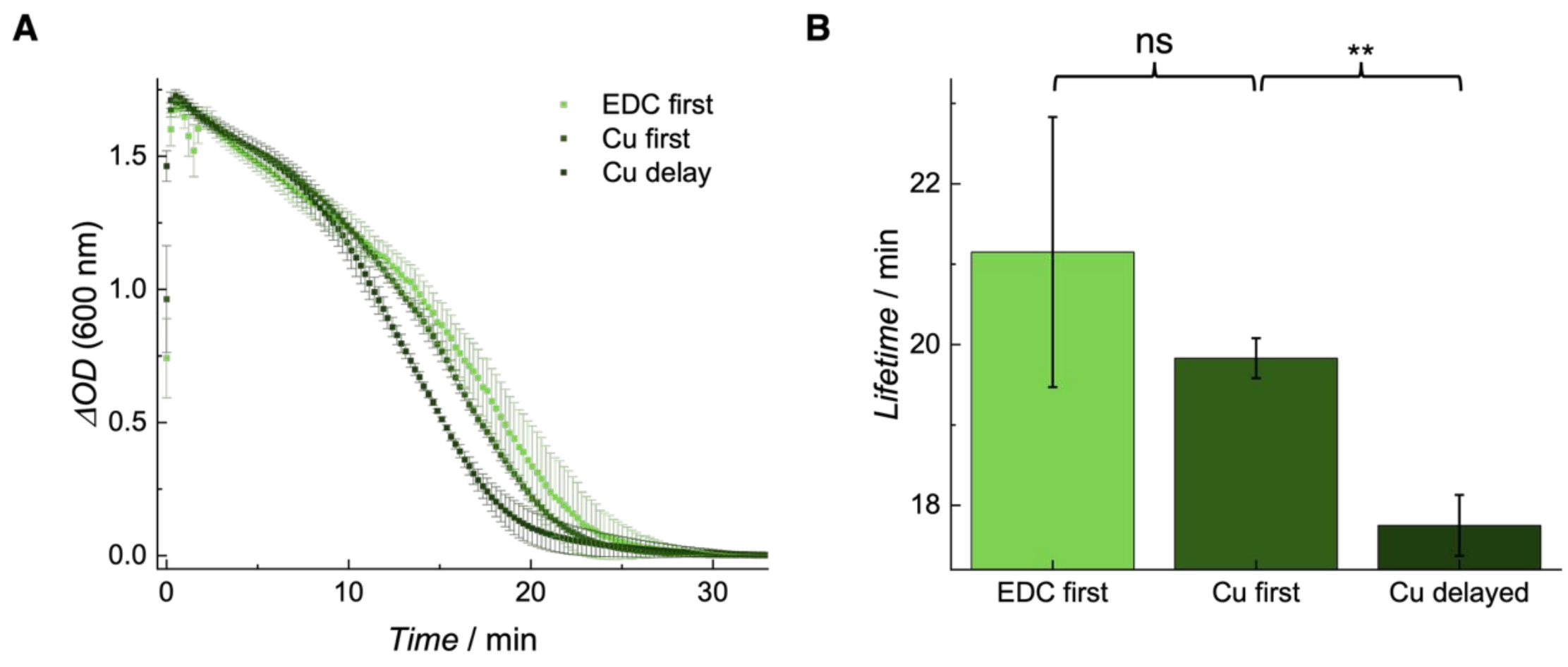

Fig. S18: a) Differences in optical density at λ = 600 nm plotted against t after addition of $CuSO_4$ (light green) or EDC (green and dark green). c (**BTAAlkyne**) = 1.0 mM at T = 25 °C in 5% EtOH / 200 mM MES buffer (pH = 5.3). All parts were added sequentially with $CuSO_4$ last following 1 min of incubation after EDC addition (EDC first, light green); EDC last following 1 min of incubation after $CuSO_4$ addition (Cu first, green) and EDC last following 10 min of incubation after $CuSO_4$ addition (Cu delay, dark green) b) The lifetime of the droplets (defined as ΔOD < 0.25) for the different experimental conditions shown in (a). The statistical analysis was performed using a two-tailed t-test assuming unequal variance (n = 3 for each condition) with ns, not significant (p > 0.05), *p < 0.05, **p < 0.01 and ***p < 0.001.

Table S18: Droplet lifetime data (given in minutes) for sequence-dependent data shown in Figure S17. The statistical analysis was performed using a two-tailed t-test assuming unequal variance. Rep. = Replicate

Experimental condition	Rep. 1	Rep. 2	Rep. 3	Average	Standard deviation	*p*-value
EDC first	12.40	12.63	13.63	12.89	0.65	–
Cu first	14.63	15.85	16.6	15.69	0.99	0.02 (to EDC first)
Cu delay	15.13	14.90	15.62	15.22	0.37	0.50 (to Cu first)

Table S19: Droplet lifetime data (given in minutes) for sequence-dependent data shown in Figure S18. The statistical analysis was performed using a two-tailed t-test assuming unequal variance. Rep. = Replicate

Experimental condition	Rep. 1	Rep. 2	Rep. 3	Average	Standard deviation	*p*-value
EDC first	23.05	19.83	20.58	21.15	1.68	–
Cu first	20.08	19.83	19.58	19.83	0.25	0.31 (to EDC first)
Cu delay	18.08	17.85	17.33	17.75	0.38	0.003 (to Cu first)

Synthetic Procedures

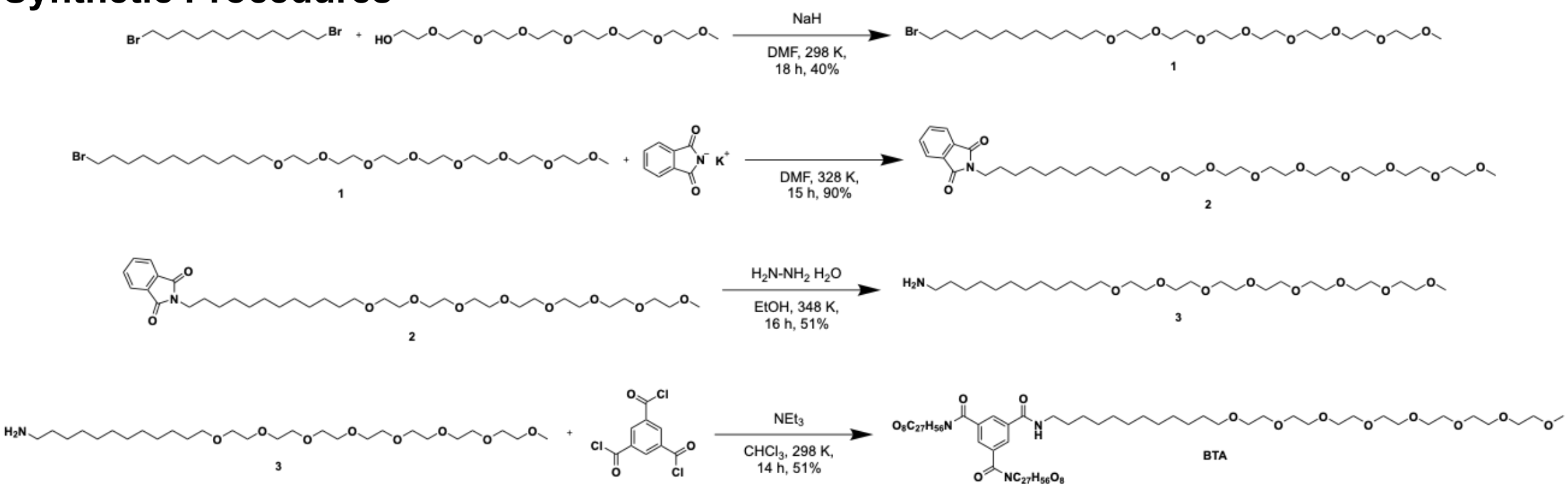

Scheme S2: Synthetic scheme for the synthesis of **BTA**.

The synthesis of **BTA** was conducted following established synthetic strategies.[8]

35-bromo-2,5,8,11,14,17,20,23-octaoxapentatriacontane (**1**)

NaH (198 mg, 4.95 mmol, 0.99 eq.) was suspended in dry DMF (10 mL) and stirred at 273 K. 2,5,8,11,14,17,20-heptaoxadocosan-22-ol (1.70 g, 5.00 mmol, 1.00 eq.) was dried using a Schlenk line for one hour, subsequently dissolved in dry DMF (5 mL), and slowly added dropwise to the stirring NaH suspension. Afterwards, the cooling bath was removed, and the mixture was stirred at room temperature for one hour. In a separate flask, 1,12-dibromododecane (4.90 g, 15.0 mmol, 3.00 eq.) was dissolved in dry DMF (25 mL) and stirred at 273 K. The mixture of 2,5,8,11,14,17,20-heptaoxadocosan-22-ol and NaH was added dropwise, the cooling bath was removed, and the reaction mixture was stirred at 298 K for 18 h. To terminate the reaction, water (50 mL) was added. The aqueous solution was extracted with cyclohexane (3 × 50 mL). The organic solvent was evaporated, and the crude product purified by column chromatography (SiO_2, MeOH/DCM 1:99 V:V to MeOH/DCM 3:97 V:V). The product was obtained as a colorless, highly viscous oil that solidified upon standing.

Note: When using higher equivalents of NaH, as has been used in previous reports,[8] we found the Br elimination-based alkene side product (asterisk in ^{1}H NMR spectrum) to become the main, or even only product of the reaction depending on the equivalents.

Yield: 1.19 g, 2.02 mmol, 40%.

^{1}H NMR (400 MHz, 298 K, $CDCl_3$): δ = 3.63 – 3.57 (m, 22H); 3.54 – 3.47 (m, 4H); 3.42 – 3.36 (m, 2H); 3.36 – 3.30 (m, 5H); 1.80 (q, J = 7.2 Hz, 2H); 1.56 – 1.47 (m, 2H); 1.41 – 1.33 (m, 2H); 1.30 – 1.18 (m, 16H) ppm.

^{13}C NMR (101 MHz, 298 K, $CDCl_3$): δ = 72.0, 71.5, 70.6, 70.5, 70.1, 59.0, 34.0, 32.8, 29.7, 29.6, 29.53, 29.50, 29.47, 29.4, 28.8, 28.2, 26.1 ppm. (Note: There is considerable overlap between peaks, particularly for the glycol range of the spectrum, making it impossible the identify all individual signals)

HR-MS (ESI, positive): m/z calculated for $C_{27}H_{55}BrO_8Na$: 609.2979; 611.2958 ([M+Na]$^+$), found: 609.2961; 611.2942.

2-(2,5,8,11,14,17,20,23-octaoxapentatriacontan-35-yl)isoindoline-1,3-dione (**2**)

Compound **1** (1.07 g, 1.82 mmol, 1.00 eq.) and Potassium phthalimide (680 mg, 3.67 mmol, 2.02 eq.) were dissolved in dry DMF (60 mL) at 358 K and stirred for 30 minutes. Without removing the oil bath, the set temperature was lowered to 328 K, and the mixture was continuously stirred for 15 h. Afterwards, the solvent was removed under reduced pressure, and the residue was dissolved in water (50 mL). The aqueous solution was extracted with MTBE (4 × 30 mL). The organic solvent was evaporated and the crude product purified by column chromatography (SiO_2, DCM to MeOH/DCM 3:97 V:V). The product was obtained as a colorless solid.

Yield: 1.07 g, 1.64 mmol, 90%.

^{1}H NMR (400 MHz, 298 K, $CDCl_3$): δ = 7.81 – 7.73 (m, 2H); 7.70 – 7.60 (m, 2H); 3.64 – 3.54 (m, 26H); 3.54 – 3.44 (m, 4H); 3.41 – 3.33 (m, 2H); 3.32 – 3.27 (m, 3H); 1.66 – 1.55 (m, 2H); 1.54 – 1.44 (m, 2H); 1.28 – 1.14 (m, 16H) ppm.

^{13}C NMR (101 MHz, 298 K, $CDCl_3$): δ = 168.3, 133.8, 132.1, 123.1, 71.9, 71.5, 70.53, 70.46, 70.0, 58.93, 38.0, 29.6, 29.5, 29.4, 29.1, 28.5, 26.8, 26.0 ppm. (Note: There is considerable overlap between peaks, particularly for the glycol range of the spectrum, making it impossible the identify all individual signals)

HR-MS (ESI, positive): *m*/*z* calculated for $C_{35}H_{59}NO_{10}Na$: 676.4037 ([*M*+Na]$^+$), found: 676.4015.

2,5,8,11,14,17,20,23-octaoxapentatriacontan-35-amine (**3**)

Compound **2** (1.07 g, 1.64 mmol, 1.00 eq.) and Hydrazine monohydrate (3 mL) were dissolved in EtOH (60 mL) and stirred at 348 K for 16 h. Afterwards, the solvent was removed under reduced pressure. The residue was dissolved in $CHCl_3$ (50 mL) and washed with an aqueous NaOH solution (1 M, 2 × 40 mL). The organic solvent was evaporated, and the crude product was purified by column chromatography (SiO_2, MeOH/DCM 5:95 V:V to MeOH/DCM 20:80 V:V). The product was obtained as a colorless oil that solidified upon standing.

Yield: 440 mg, 0.84 mmol, 51%.

^{1}H NMR (400 MHz, 298 K, $CDCl_3$): δ = 3.66 – 3.61 (m, 24H); 3.58 – 3.52 (m, 4H); 3.45 – 3.40 (m, 2H); 3.36 (s, 3H); 2.66 (t, *J* = 7.1 Hz, 2H); 1.59 – 1.47 (m, 4H); 1.45 – 1.39 (m, 2H); 1.33 – 1.23 (m, 16H) ppm.

^{13}C NMR (101 MHz, 298 K, $CDCl_3$): δ = 71.8, 71.3, 70.4, 70.3, 69.9, 58.8, 53.4, 42.0, 33.6, 29.5, 29.41, 29.38, 29.3, 26.7, 25.9 ppm. (Note: There is considerable overlap between peaks, particularly for the glycol range of the spectrum, making it impossible the identify all individual signals)

HR-MS (ESI, positive): *m*/*z* calculated for $C_{27}H_{58}NO_8$: 524.4163 ([*M*+H]$^+$), found: 524.4148. *m*/*z* calculated for $C_{27}H_{57}NO_8Na$: 546.3982 ([*M*+Na]$^+$), found: 546.3964.

BTA

Compound **3** (162 mg, 0.31 mmol, 3.1 eq) was dissolved in dry $CHCl_3$ (5 mL). NEt_3 (0.09 mL, 66.7 mg, 0.66 mmol, 6.6 eq.) was added and the mixture was stirred at 273 K. Trimesoylchloride (26.5 mg, 0.10 mmol, 1.0 eq.), dissolved in dry $CHCl_3$ (1 mL), was added dropwise. The cooling bath was removed, and the reaction mixture was stirred at 298 K for 14 h. Afterwards, the solvent was removed under reduced pressure, and the crude product was purified by column chromatography (SiO_2, MeOH/DCM 5:95 V:V). The product was obtained as a colorless solid.

Yield: 87.8 mg, 0.051 mmol, 51%.

^{1}H NMR (500 MHz, 298 K, $CDCl_3$): δ = 8.39 (s, 3H); 6.93 (t, J = 5.7 Hz, 3H); 3.63 – 3.56 (m, 72H); 3.55 – 3.51 (m, 6H); 3.51 – 3.48 (m, 6H); 3.42 – 3.37 (m, 12H); 3.33 (s, 9H); 1.60 – 1.48 (m, 12H); 1.37 – 1.21 (m, 48H) ppm.

^{13}C NMR (101 MHz, 298 K, $CDCl_3$): δ = 165.9, 135.4, 128.3, 72.0, 71.6, 70.71, 70.66, 70.64, 70.58, 70.1, 59.1, 40.4, 29.7, 29.59, 29.56, 29.5, 29.4, 27.1, 26.1 ppm. (Note: There is considerable overlap between peaks, particularly for the glycol range of the spectrum, making it impossible the identify all individual signals)

HR-MS (ESI, positive): m/z calculated for $C_{90}H_{171}N_3O_{27}Na$: 1749.1999; 1750.2033; 1751.2066 ([M+Na]$^+$), found: 1749.1975; 1750.2007; 1751.2028. m/z calculated for $C_{90}H_{171}N_3O_{27}Na_2$: 886.0948; 886.5965 ([$M$+2Na]$^{2+}$), found: 886.0923; 886.5935. m/z calculated for $C_{90}H_{171}N_3O_{27}Na_3$: 598.3931; 598.7276 ([$M$+3Na]$^{3+}$), found: 598.3920; 598.7258.

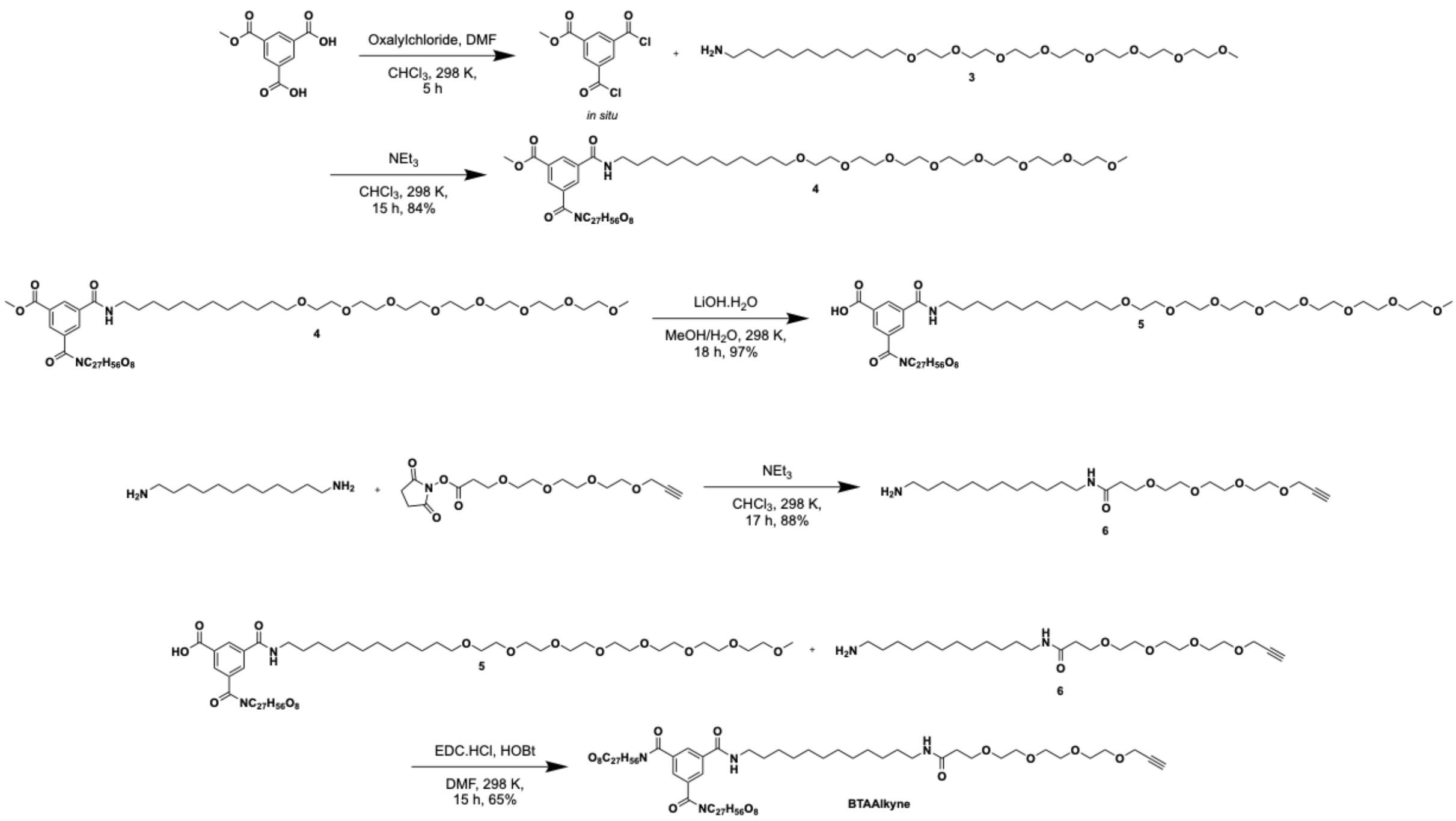

Scheme S3: Synthetic scheme for the synthesis of **BTAAlkyne**.

The synthesis of **BTAAlkyne** was conducted based on established synthetic strategies.[8]

Methyl 3,5-bis((2,5,8,11,14,17,20,23-octaoxapentatriacontan-35-yl)carbamoyl)benzoate (**4**)

Oxalylchloride (0.25 mL, 3.0 mmol, 3.2 eq.) and a catalytic amount of DMF (1 drop) were dissolved in dry $CHCl_3$ (25 mL) and stirred at 273 K. 5-(methoxycarbonyl)isophthalic acid (210 mg, 0.93 mmol, 1.00 eq.) was added as a solid and allowed to slowly dissolve at 273 K while stirring for 40 minutes. Afterwards, the cooling bath was removed, and the mixture was continuously stirred at 298 K for 5 h. The solvent was removed under reduced pressure, and the excess Oxalylchloride was removed by coevaporation with toluene (3, × 10 mL). The residue was dissolved in dry $CHCl_3$ (25 mL) and stirred at 273 K. NEt_3 (0.6 mL) was added dropwise. Compound **3**, dissolved in dry $CHCl_3$ (10 mL), was added slowly. The cooling bath was removed, and the mixture was stirred at 298 K for 15 h. Afterwards, the solvent was removed under reduced pressure. The residue was dissolved in $CHCl_3$ (40 mL). The organic phase was washed with an aqueous HCl solution (1M, 3 × 40 mL) and brine (40 mL). The solvent was evaporated and the crude product purified by column chromatography (SiO_2, MeOH/DCM 3:97 V:V). The product was obtained as a pale-yellow oil.

Yield: 960 mg, 0.78 mmol, 84%.

^{1}H NMR (400 MHz, 298 K, $CDCl_3$): δ = 8.55 (d, J = 1.8 Hz, 2H); 8.42 (t, J = 1.8 Hz, 1H); 6.78 (t, J = 5.7 Hz, 2H); 3.92 (s, 3H); 3.64 – 3.57 (m, 48H); 3.56 – 3.49 (m, 8H); 3.45 – 3.38 (m, 8H); 3.33 (s, 6H); 1.62 – 1.49 (m, 8H); 1.37 – 1.21 (m, 32H) ppm.

^{13}C NMR (101 MHz, 298 K, $CDCl_3$): δ = 165.9, 165.7, 135.6, 131.1, 130.8, 129.6, 72.0, 71.6, 70.72, 70.68, 70.66, 70.6, 70.2, 59.1, 52.6, 40.5, 29.7, 29.62, 29.59, 29.5, 29.4, 27.1, 26.2 ppm. (Note: There is considerable overlap between peaks, particularly for the glycol range of the spectrum, making it impossible the identify all individual signals)

HR-MS (ESI, positive): *m*/*z* calculated for $C_{64}H_{118}N_2O_{20}Na$: 1257.8177, 1258.8210 ([*M*+Na]$^{+}$), found: 1257.8141; 1258.8176. *m*/*z* calculated for $C_{64}H_{118}N_2O_{20}Na_2$: 845.5610; 846.0626 ([*M*+2Na]$^{2+}$), found: 640.4022; 640.9027.

3,5-bis((2,5,8,11,14,17,20,23-octaoxapentatriacontan-35-yl)carbamoyl)benzoic acid (**5**)

Compound **4** (175 mg, 0.140 mmol, 1.0 eq.) $LiOH{\cdot}H_2O$ (36 mg, 0.75 mmol, 5.4 eq.) and water (72 μL) were dissolved in MeOH (7 mL) and stirred at room temperature for 18 h. Afterwards, the solvent was removed under reduced pressure, and the residue was dissolved in water (10 mL). The aqueous solution was acidified using concentrated HCl (2 mL) and extracted with $CHCl_3$ (4 × 15 mL). The solvent was removed, yielding the product as a yellow oil.

Yield: 166.4 mg, 0.136 mmol, 97%.

^{1}H NMR (400 MHz, 298 K, $CDCl_3$): δ = 8.60 – 8.58 (m, 2H); 8.53 – 8.50 (m, 1H); 7.09 (t, *J* = 5.6 Hz, 2H); 3.65 – 3.58 (m, 48H); 3.57 – 3.50 (m, 8H); 3.47 – 3.38 (m, 8H); 3.34 (s, 6H); 1.66 – 1.57 (m, 4H); 1.55 – 1.48 (m, 4H); 1.39 – 1.20 (m, 32H) ppm.

^{13}C NMR (101 MHz, 298 K, $CDCl_3$): δ = 167.3, 166.1, 135.5, 131.2, 130.1, 72.0, 71.6, 70.71, 70.65, 70.6, 70.1, 59.1, 40.5, 29.7, 29.62, 29.58, 29.55, 29.5, 29.4, 27.1, 26.1 ppm. (Note: There is considerable overlap between peaks, particularly for the glycol range of the spectrum, making it impossible the identify all individual signals)

HR-MS (ESI, positive): *m*/*z* calculated for $C_{63}H_{116}N_2O_{20}Na$: 1243.8020, 1244.8054 ([*M*+Na]$^+$), found: 1243.8009; 1244.8051. *m*/*z* calculated for $C_{63}H_{116}N_2O_{20}Na_2$: 633.3959; 633.8976 ([*M*+2Na]$^{2+}$), found: 633.3950; 633.8964.

N-(12-aminododecyl)-4,7,10,13-tetraoxahexadec-15-ynamide (**6**)

1,12-diaminododecane (3.10 g, 15.5 mmol, 10.0 eq.) and NEt_3 (3.16 g, 31.0 mmol, 20.0 eq.) were dissolved in $CHCl_3$ (70 mL). Propargyl-PEG4-NHS ester (553 mg, 1.55 mmol, 1.0 eq.) dissolved in $CHCl_3$ (5 mL) was added dropwise, and the mixture was stirred at 298 K for 17 h. The solvent was removed under reduced pressure, and the crude product purified by column chromatography (SiO_2, NEt_3/MeOH/DCM 0.1:5:94.9 V:V:V). The product was obtained as a white solid.

Yield: 389 mg, 0.88 mmol, 88%.

^{1}H NMR (400 MHz, 298 K, $CDCl_3$): δ = 6.63 (s, 1H); 4.22 (d, *J* = 2.4 Hz, 2H); 3.72 (t, *J* = 5.8 Hz, 2H); 3.71 – 3.65 (m, 4H); 3.64 (s, 4H); 3.62 (s, 4H); 3.24 – 3.17 (m, 2H); 2.72 – 2.67 (m, 2H); 2.48 (t, *J* = 5.8 Hz, 2H); 2.43 (t, *J* = 2.4 Hz, 1H); 2.38 – 2.31 (brs, 2H); 1.51 – 1.42 (m, 4H); 1.33 – 1.21 (m, 16H) ppm.

^{13}C NMR (101 MHz, 298 K, $CDCl_3$): δ = 171.5, 79.7, 74.8, 70.8, 70.61, 70.56, 70.5, 70.4, 69.1, 67.6, 58.6, 42.1, 39.6, 37.1, 33.1, 29.7, 29.64, 29.59, 29.51, 29.4, 27.1, 27.0 ppm. (Note: There is considerable overlap between peaks, particularly for the glycol range of the spectrum, making it impossible the identify all individual signals)

HR-MS (ESI, positive): *m*/*z* calculated for $C_{24}H_{47}N_2O_5$: 443.3485 ([*M*+H]$^+$), found:443.3472. *m*/*z* calculated for $C_{24}H_{46}N_2O_5Na$: 465.3305 ([*M*+Na]$^+$), found: 465.3291.

BTAAlkyne

Compound **5** (183 mg, 0.15 mmol, 1.0 eq.), EDC·HCl (44 mg, 0.22 mmol, 1.5 eq.) and HOBt (23 mg, 0.16 mmol, 1.1 eq.) were dissolved in dry $CHCl_3$ (6 mL) and stirred at 298 K for 1 h. Compound **6** (66.4 mg, 0.15 mmol, 1.0 eq.) dissolved in dry $CHCl_3$ (2 mL) was added and the mixture was continuously stirred at 298 K for 15 h. To terminate the reaction, water (20 mL) was added, as well as a small amount of brine to aid phase separation during extraction. The aqueous phase was extracted with $CHCl_3$ (3 × 20 mL). The organic solvent was evaporated, and the crude product was purified by column chromatography (SiO_2, DCM to MeOH/DCM 5:95 V:V). The product was obtained as a pale yellow oil which solidified upon standing.

Yield: 159.5 mg, 0.097 mmol, 65%.

^1H NMR (500 MHz, 298 K, $CDCl_3$): δ = 8.34 (s, 3H); 7.17 – 7.07 (m, 3H); 6.51 (t, J = 5.6 Hz, 1H); 4.11 – 4.08 (m, 2H); 3.63 – 3.50 (m, 62H); 3.49 – 3.42 (m, 8H); 3.36 – 3.29 (m, 8H); 3.26 (s, 6H); 3.15 – 3.07 (m, 2H); 2.61 – 2.51 (m, 2H); 2.38 (t, J = 2.4 Hz, 1H); 2.34 (t, J = 5.9 Hz, 2H); 1.54 – 1.35 (m, 12H); 1.30 – 1.14 (m, 48H) ppm.

^{13}C NMR (101 MHz, 298 K, $CDCl_3$): δ = 171.4, 166.0, 135.2, 128.3, 79.5, 74.7, 71.8, 71.4, 70.6, 70.5, 70.4, 70.34, 70.31, 70.2, 70.0, 69.0, 67.3, 58.9, 58.3, 40.3, 39.4, 36.9, 29.53, 29.46, 29.41, 29.37, 29.3, 29.23, 29.19, 26.99, 26.96, 26.8, 26.0 ppm. (Note: There is considerable overlap between peaks, particularly for the glycol range of the spectrum, making it impossible the identify all individual signals)

HR-MS (ESI, positive): m/z calculated for $C_{87}H_{160}N_4O_{24}Na$: 1668.1322; 1669.1355; 1670.1389 ($[M+Na]^+$), found: 1668.1284; 1669.1319; 1670.1350. m/z calculated for $C_{87}H_{160}N_4O_{24}Na_2$: 845.5610; 846.0626 ($[M+2Na]^{2+}$), found: 845.5585; 846.0598. m/z calculated for $C_{87}H_{160}N_4O_{24}Na_3$: 571.3706; 571.7050 ($[M+3Na]^{3+}$), found: 571.3693; 571.7036.

Literature

[1] Späth, F.; Donau, C.; Bergmann, A. M.; Kränzlein, M.; Synatschke, C. V.; Rieger, B.; Boekhoven, J. *J. Am. Chem. Soc.*, **2021**, *143*, 4782–4789.

[2] Wenisch, M.; Li, Y.; Braun, M. G.; Eylert, L.; Späth, F.; Poprawa, S. M.; Rieger, B.; Synatschke, C. V.; Niederholtmeyer, H.; Boekhoven, J. *Chem*, **2025**, *11*, 102578.

[3] Roosma, J.; Mes, T.; Leclere, P.; Palmans, A. R. A.; Meijer, E. W. *J. Am. Chem. Soc.* **2008**, *130*, 1120–1121.

[4] Kang, M.; Day, C. D.; Kenworthy, A. K.; DiBenedetto, E. *Traffic* **2012**, *13*, 1589.

[5] CurveFitter (ImageJ API) https://imagej.net/ij/developer/api/ij/ij/measure/CurveFitter.html

[6] Korevaar, P. M.; Schaefer, C.; de Greef, T. F. A.; Meijer, E. W. *J. Am. Chem. Soc.* **2012**, *134*, 13482–13491.

[7] a) Donau, C.; Späth, F.; Sosson, M.; Kriebisch, B. A. K.; Schnitter, F.; Tena-Solsona, M.; Kang, H. S.; Salibi, E.; Sattler, M.; Mutschler, H.; Boekhoven, J. *Nat. Commun.* **2020**, *11*, 5167; b) Tena-Solsona, M.; Riess, B.; Grotsch, R. K.; Lohrer, F. C.; Wanzke, C.; Kasdorf, B.; Bausch, A. R.; Müller-Buschbaum, P.; Lieleg, O.; Boekhoven, J. *Nat. Commun.* **2017**, *8*, 15895; c) Schnitter, F.; Boekhoven, J. *ChemSystemsChem*, **2021**, *3*, e2000037.

[8] Leenders, C. M. A.; Mes, T.; Baker, M. B.; Koenigs, M. M. E.; Besenius, P.; Palmans, A. R. A.; Meijer, E. W. *Mater. Horiz.* **2014**, *1*, 116–120.

Characterization Data

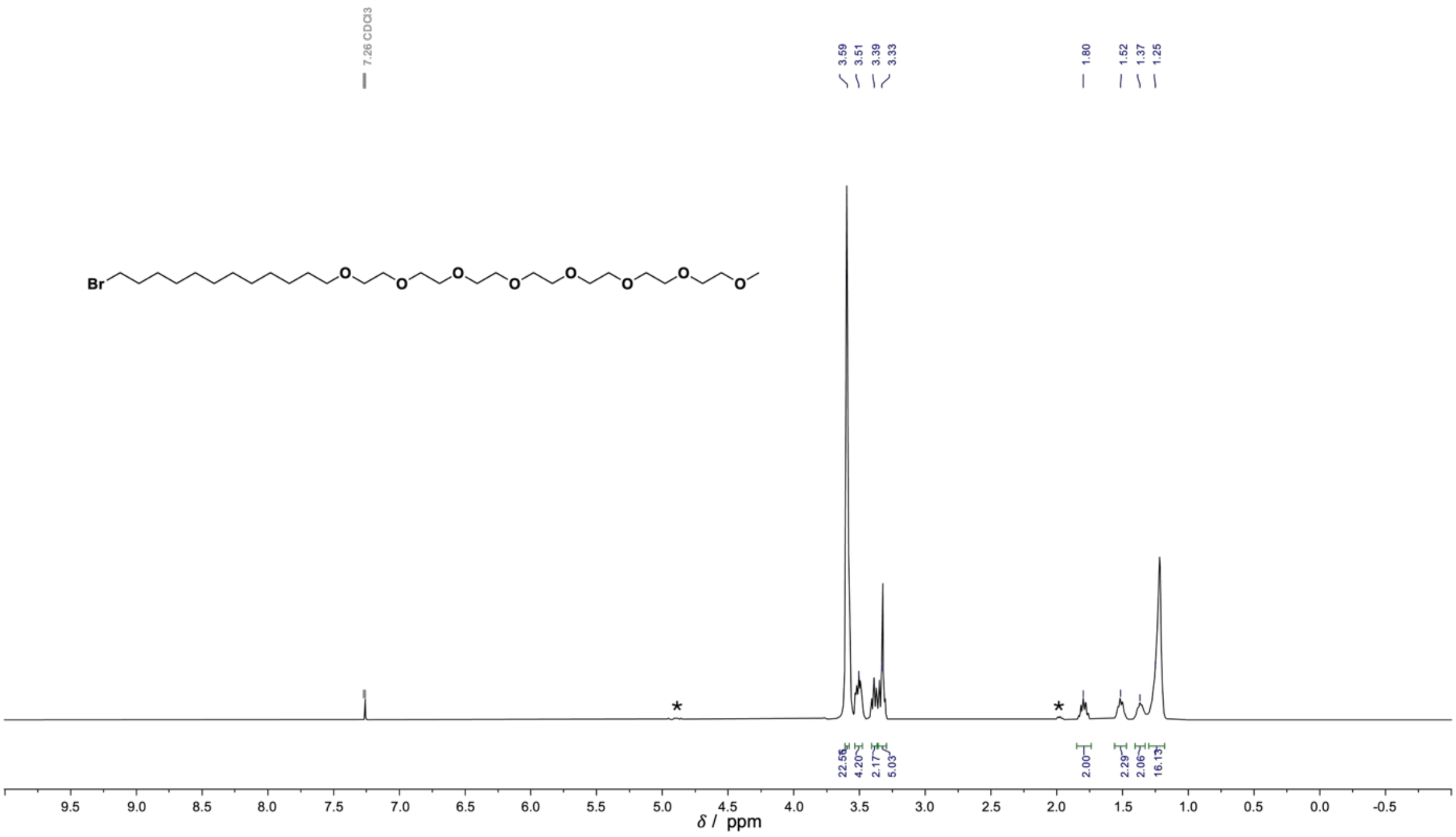

Fig. S19: ^{1}H NMR spectrum of compound **1** (400 MHz, 298 K, $CDCl_3$).

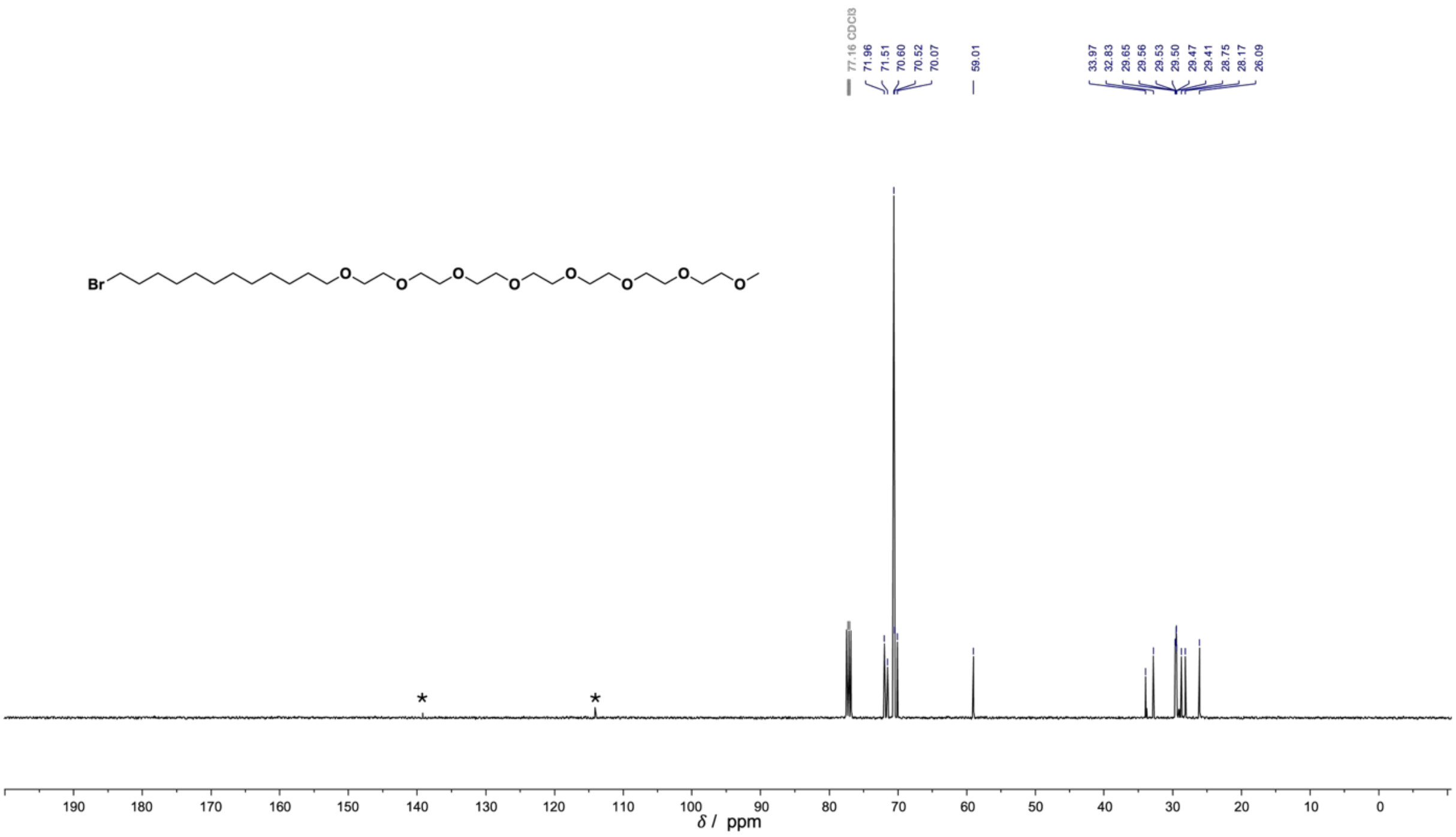

Fig. S20: ^{13}C NMR spectrum of compound **1** (101 MHz, 298 K, $CDCl_3$).

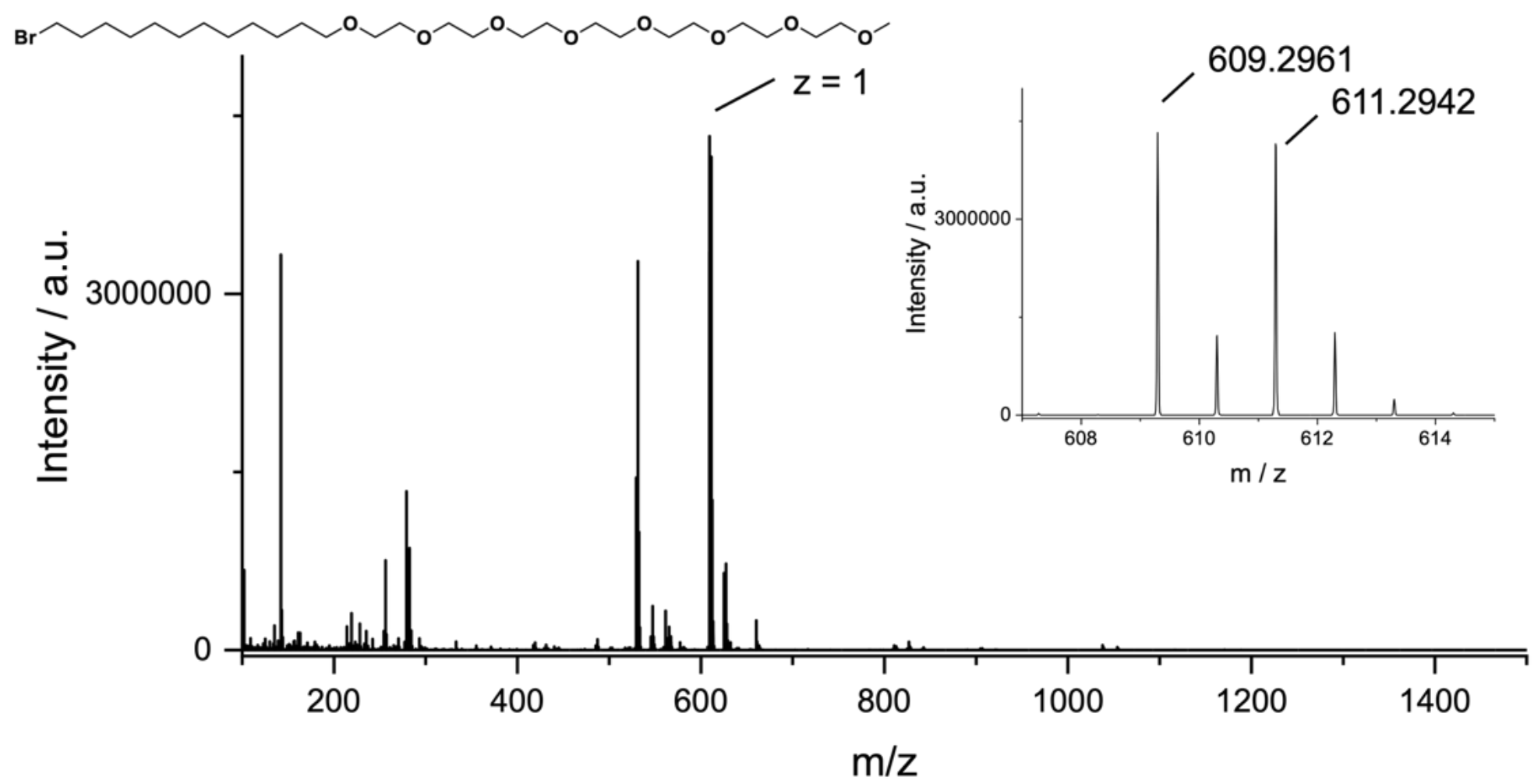

Fig. S21: HR-MS (ESI, positive) spectrum of compound **1**.

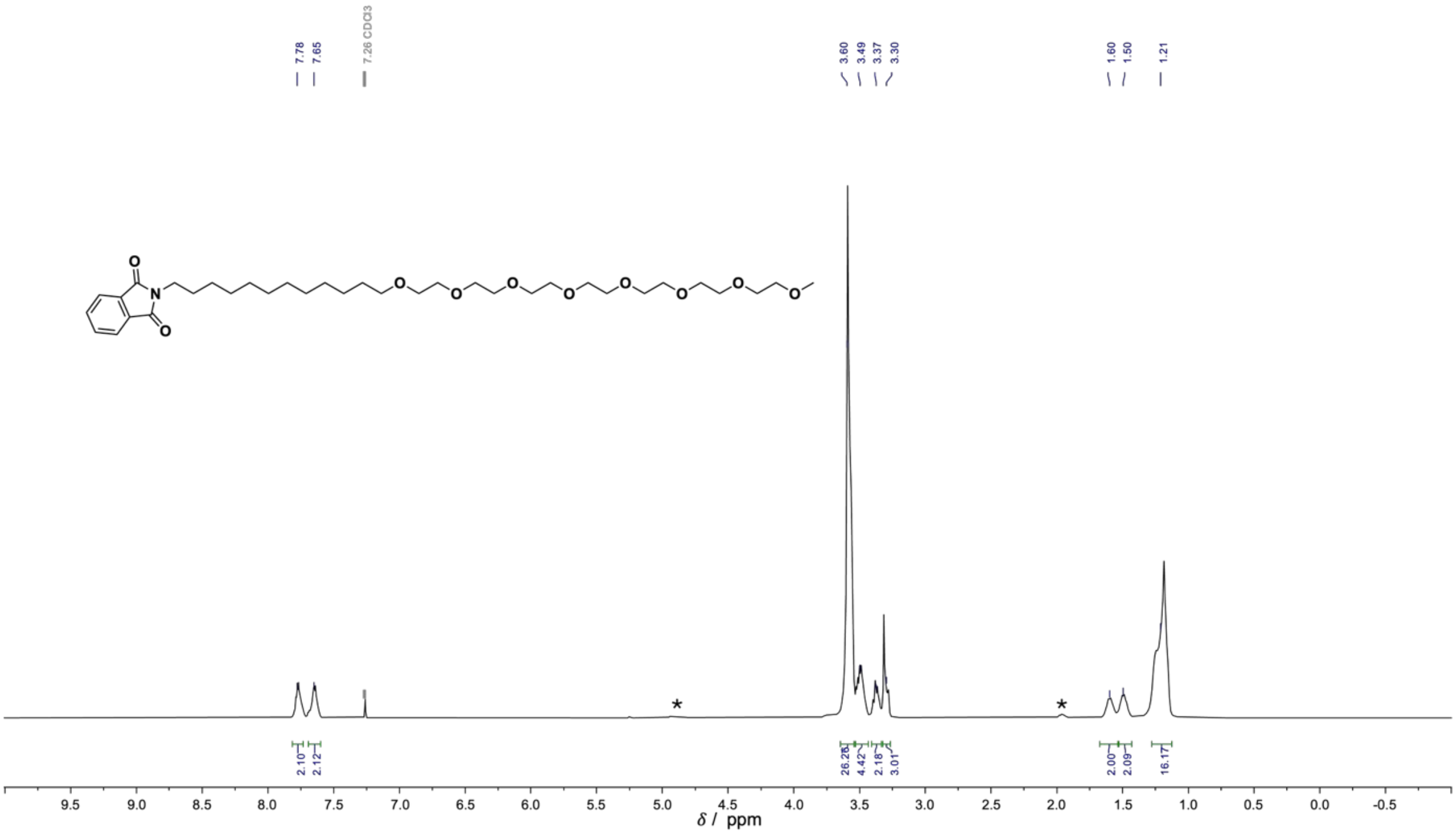

Fig. S22: ^{1}H NMR spectrum of compound **2** (400 MHz, 298 K, $CDCl_3$).

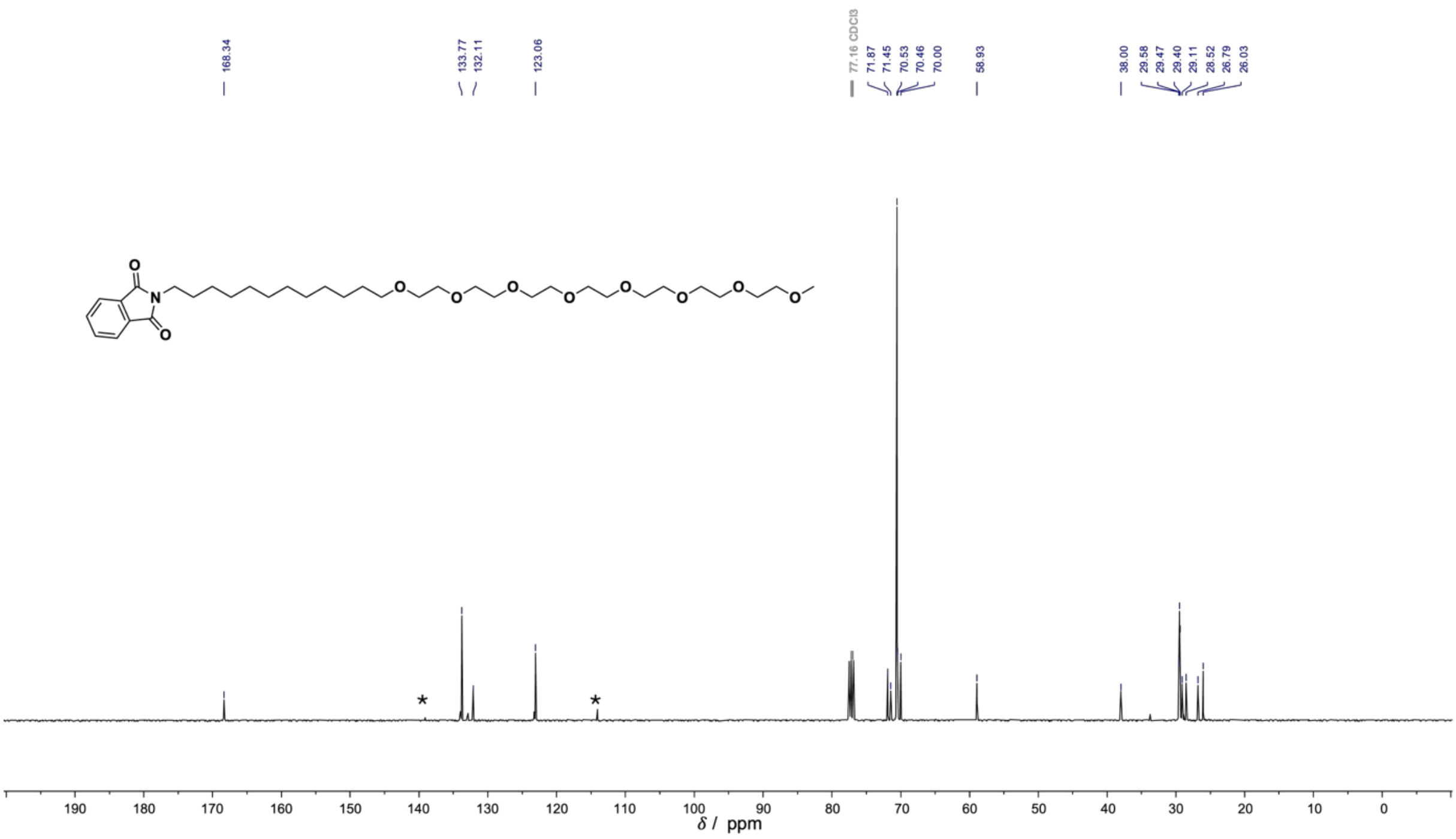

Fig. S23: ^{13}C NMR spectrum of compound **2** (101 MHz, 298 K, $CDCl_3$).

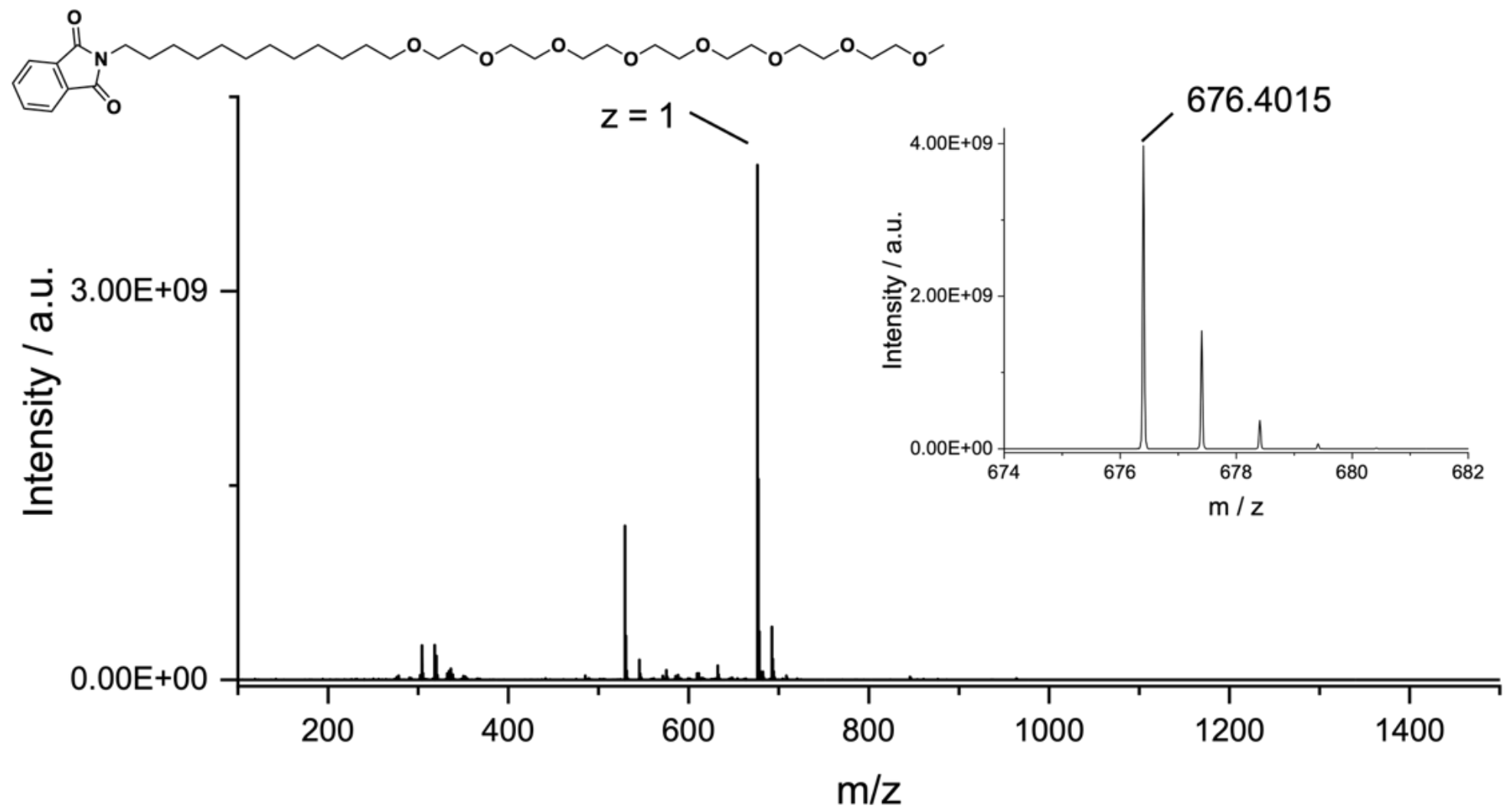

Fig. S24: HR-MS (ESI, positive) spectrum of compound **2**.

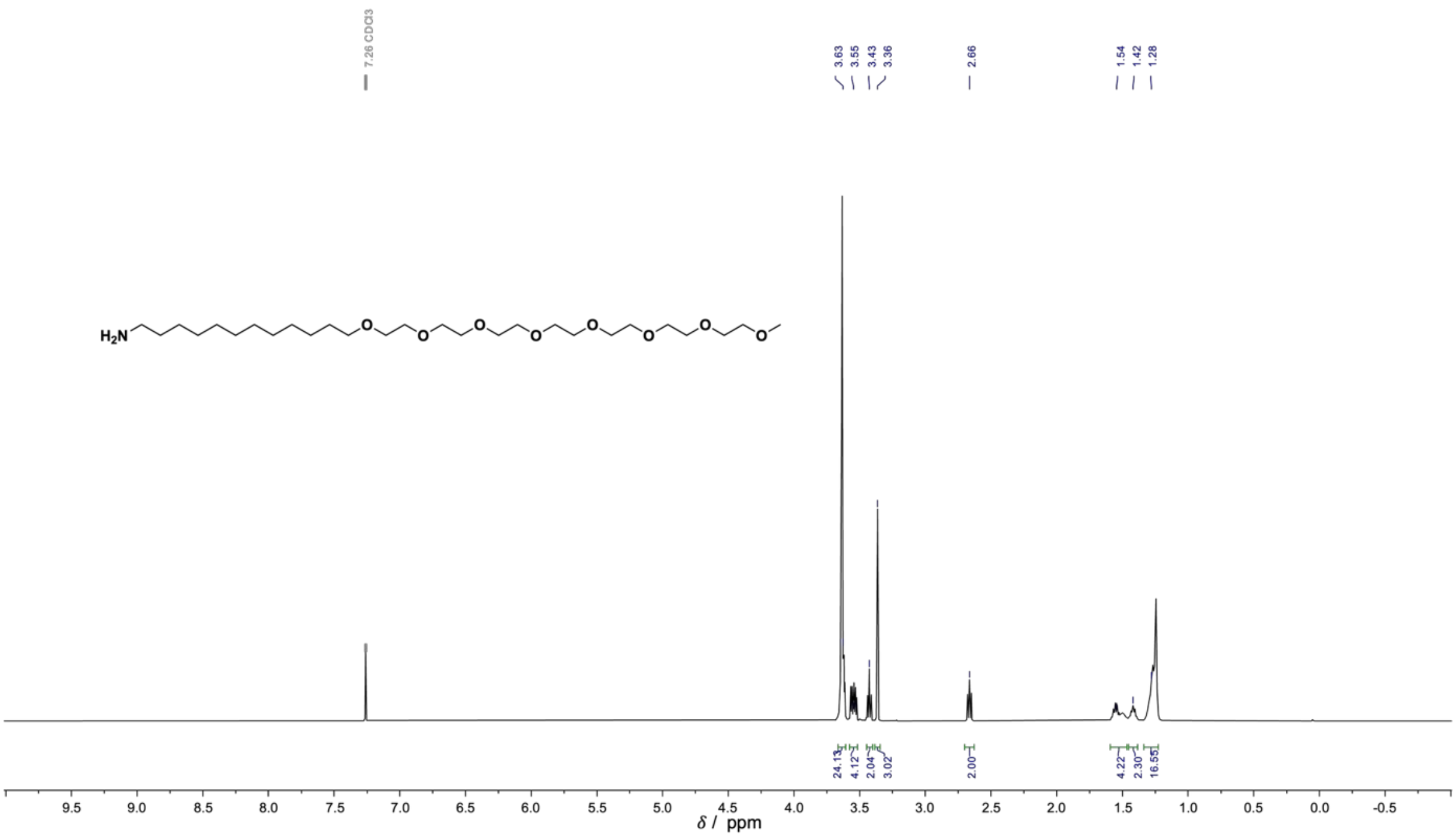

Fig. S25: ^{1}H NMR spectrum of compound **3** (400 MHz, 298 K, $CDCl_3$).

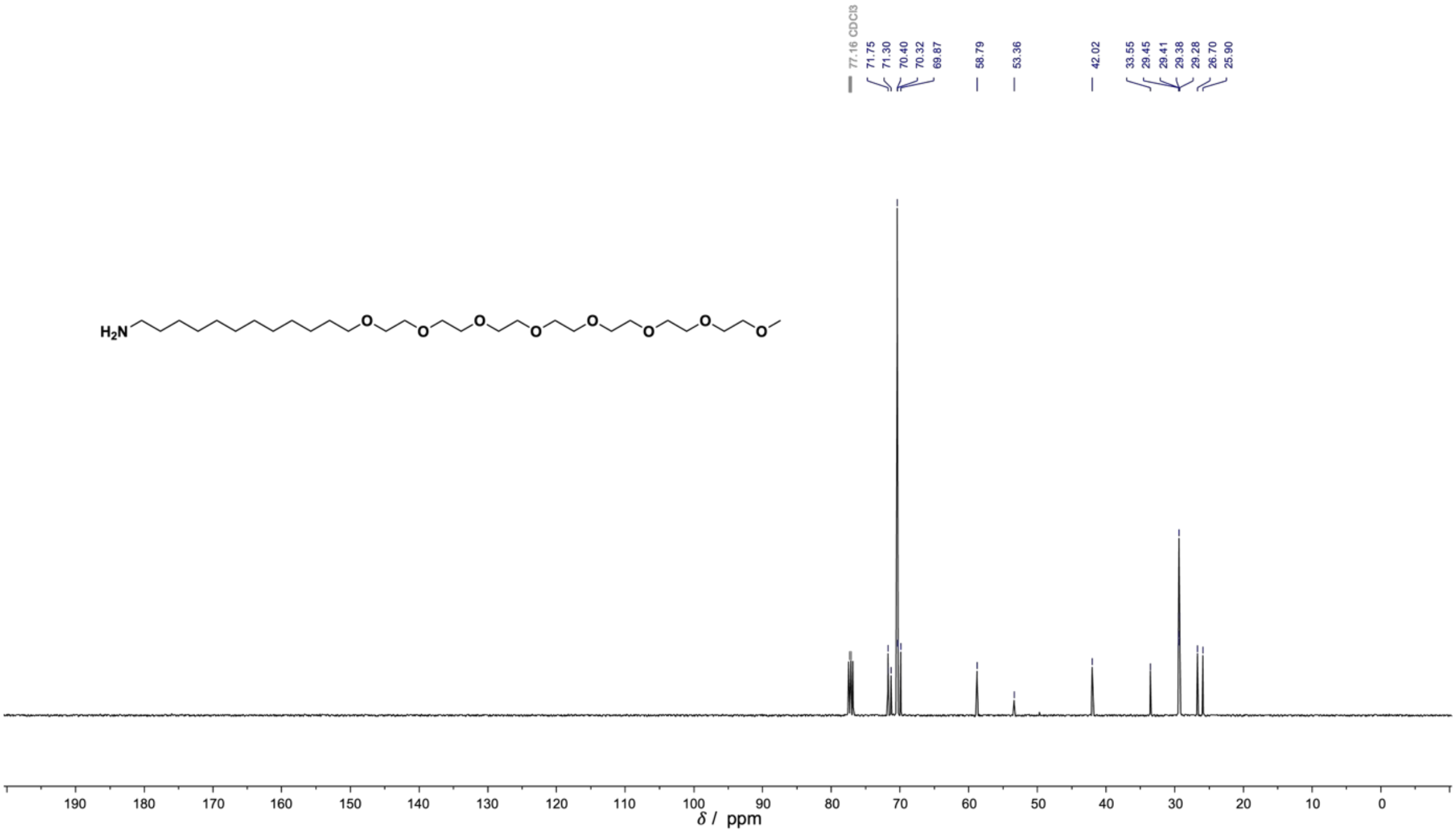

Fig. S26: ^{13}C NMR spectrum of compound **3** (101 MHz, 298 K, $CDCl_3$).

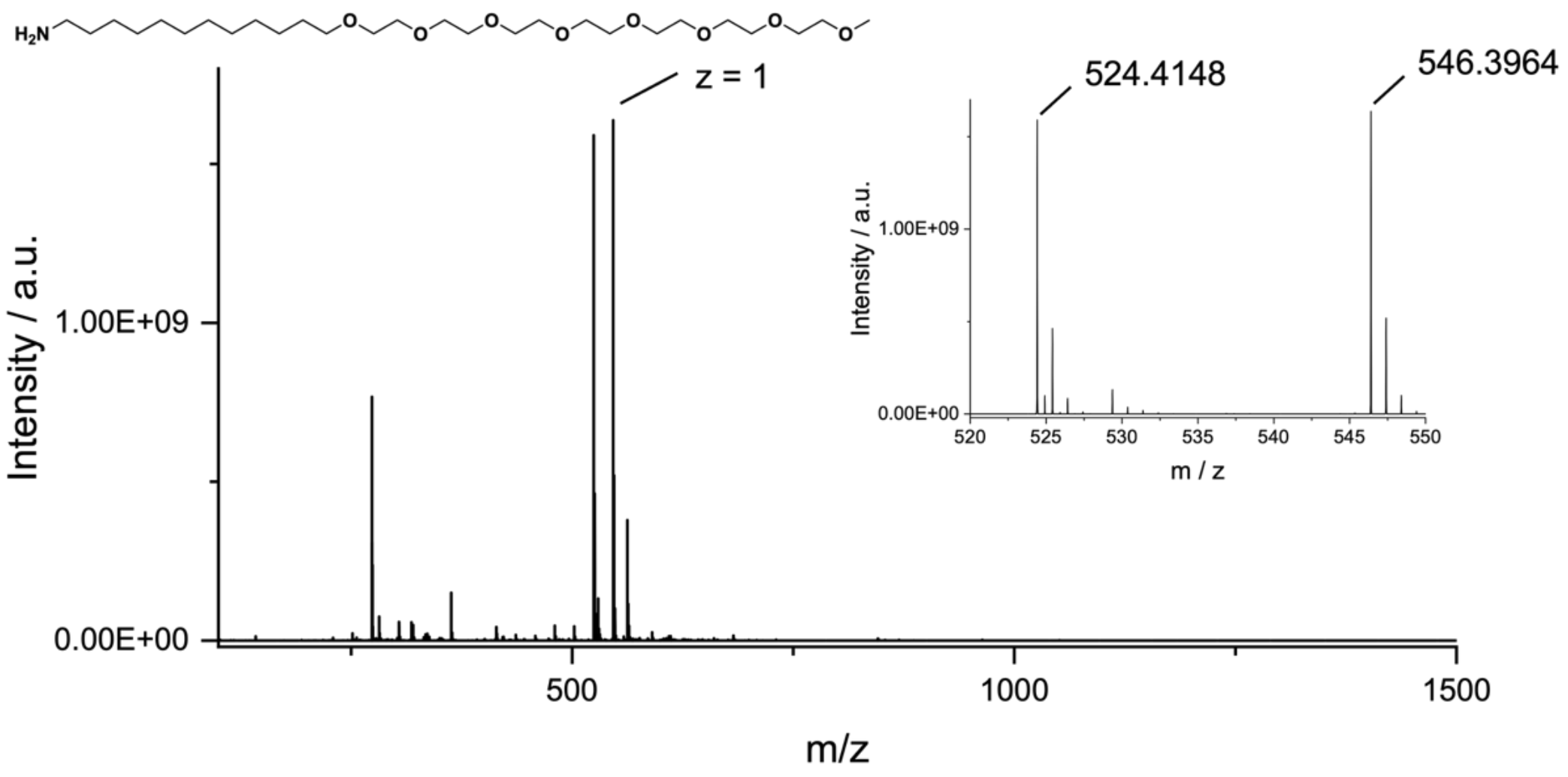

Fig. S27: HR-MS (ESI, positive) spectrum of compound **3**.

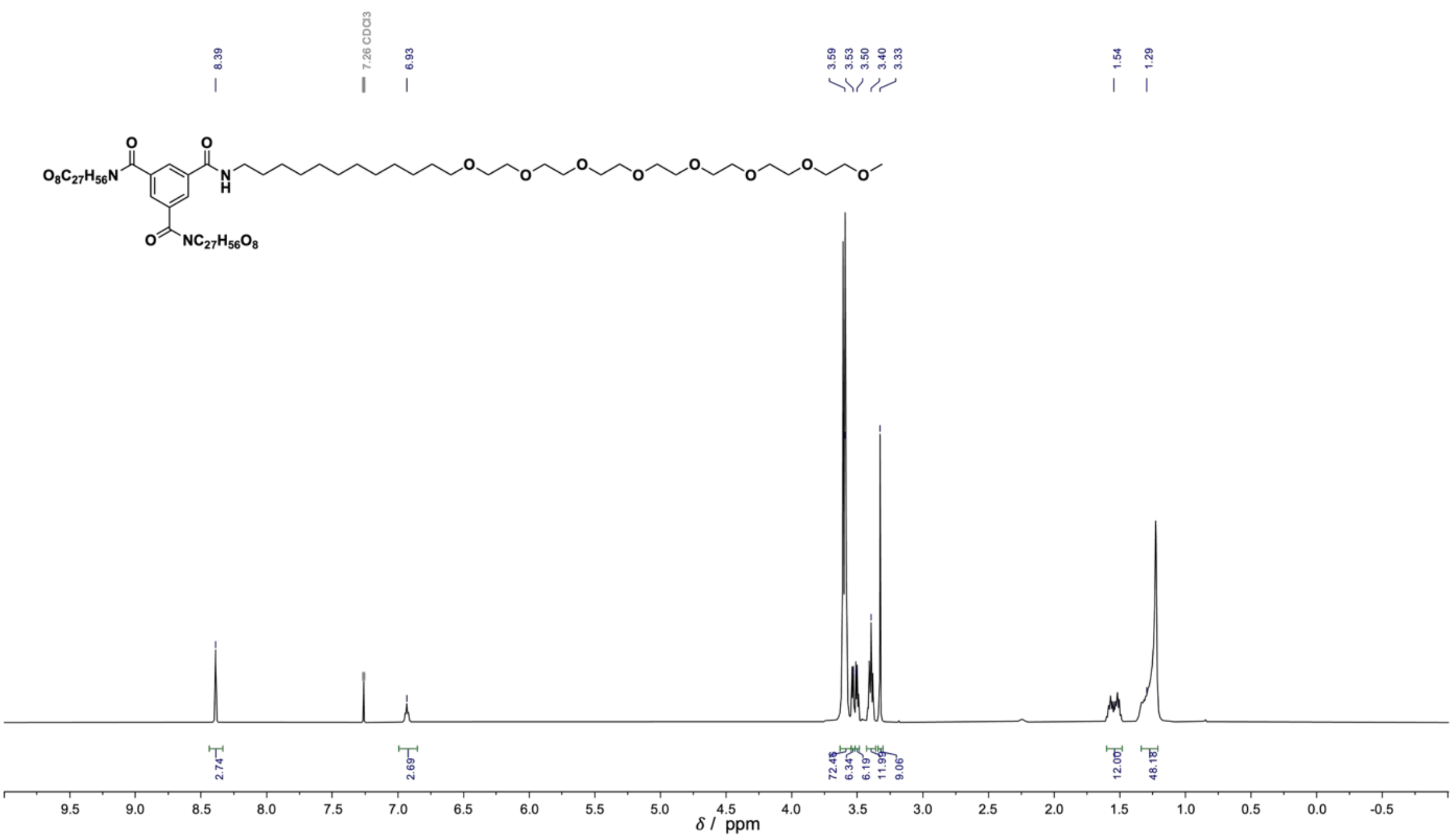

Fig. S28: ^{1}H NMR spectrum of compound **BTA** (500 MHz, 298 K, $CDCl_3$).

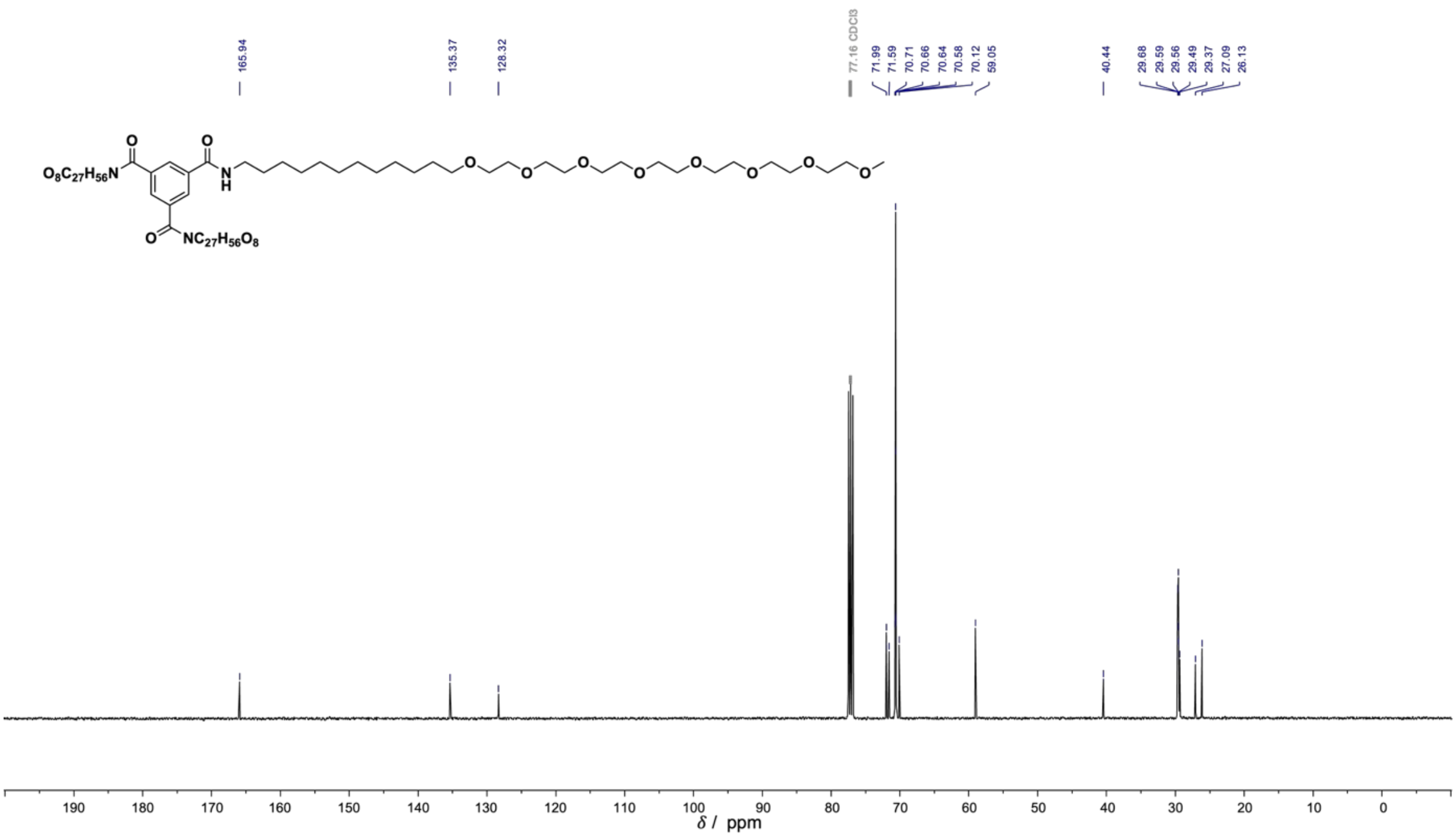

Fig. S29: ^{13}C NMR spectrum of compound **BTA** (101 MHz, 298 K, $CDCl_3$).

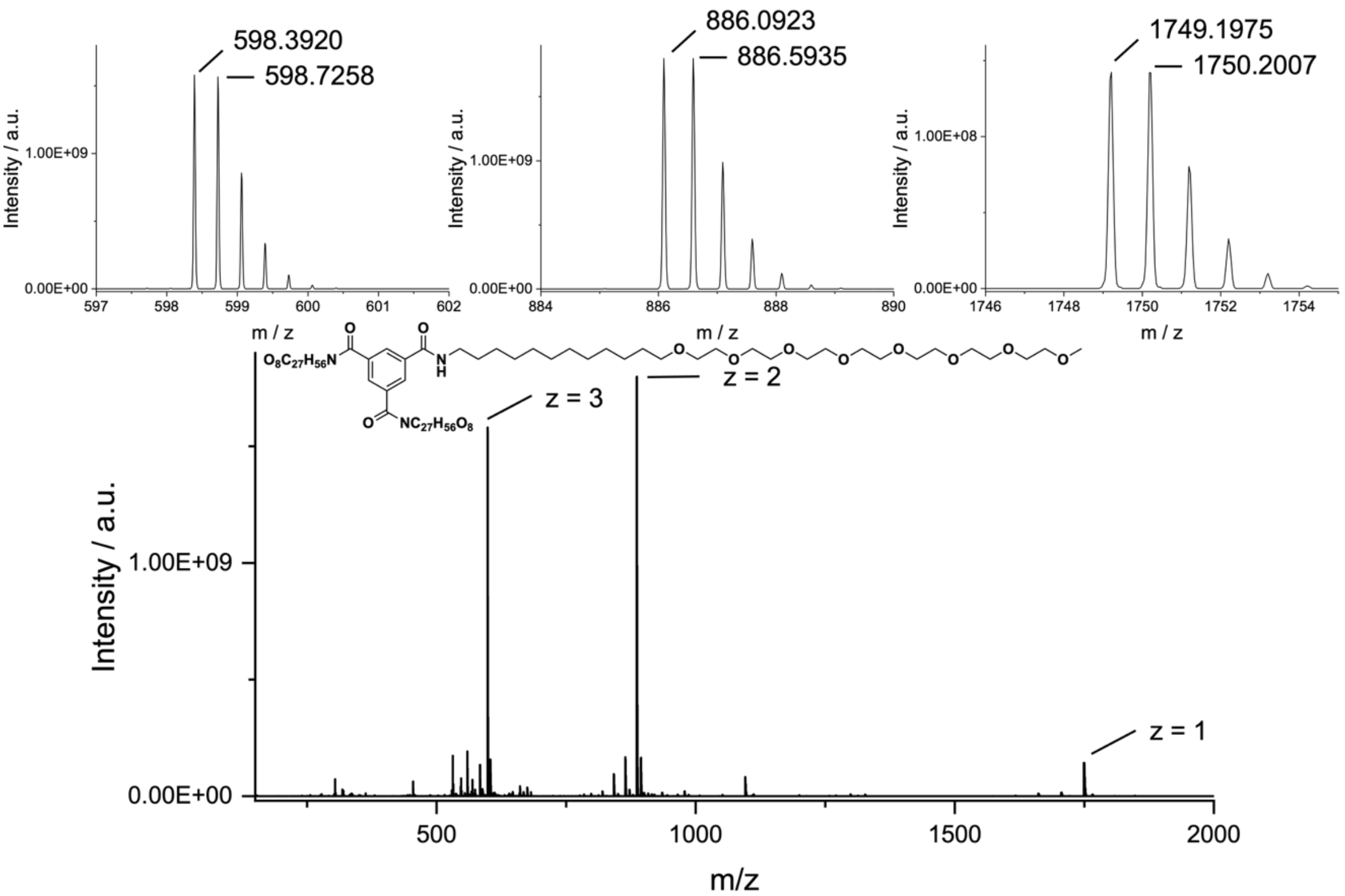

Fig. S30: HR-MS (ESI, positive) spectrum of compound **BTA**.

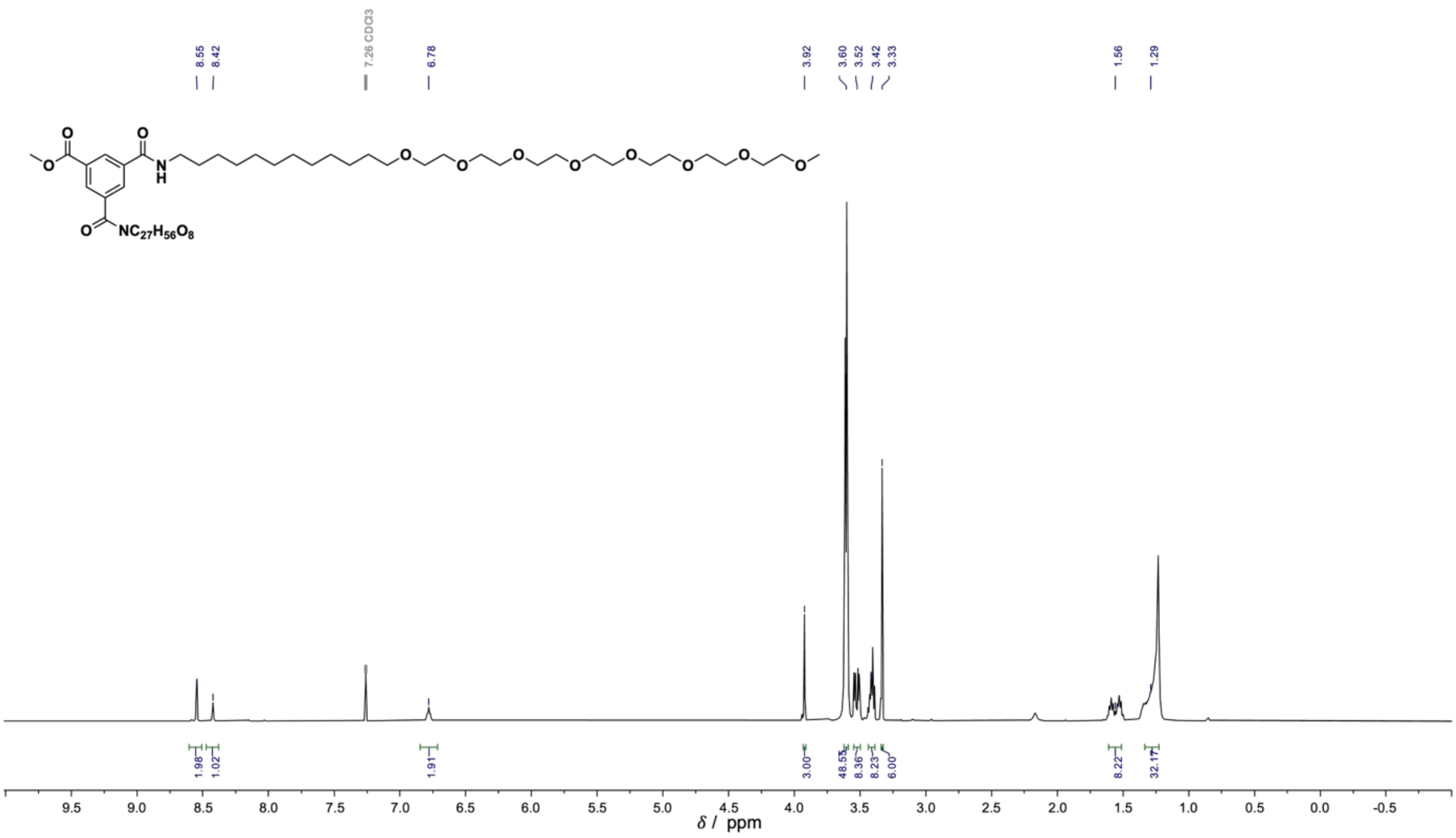

Fig. S31: ^{1}H NMR spectrum of compound **4** (400 MHz, 298 K, $CDCl_3$).

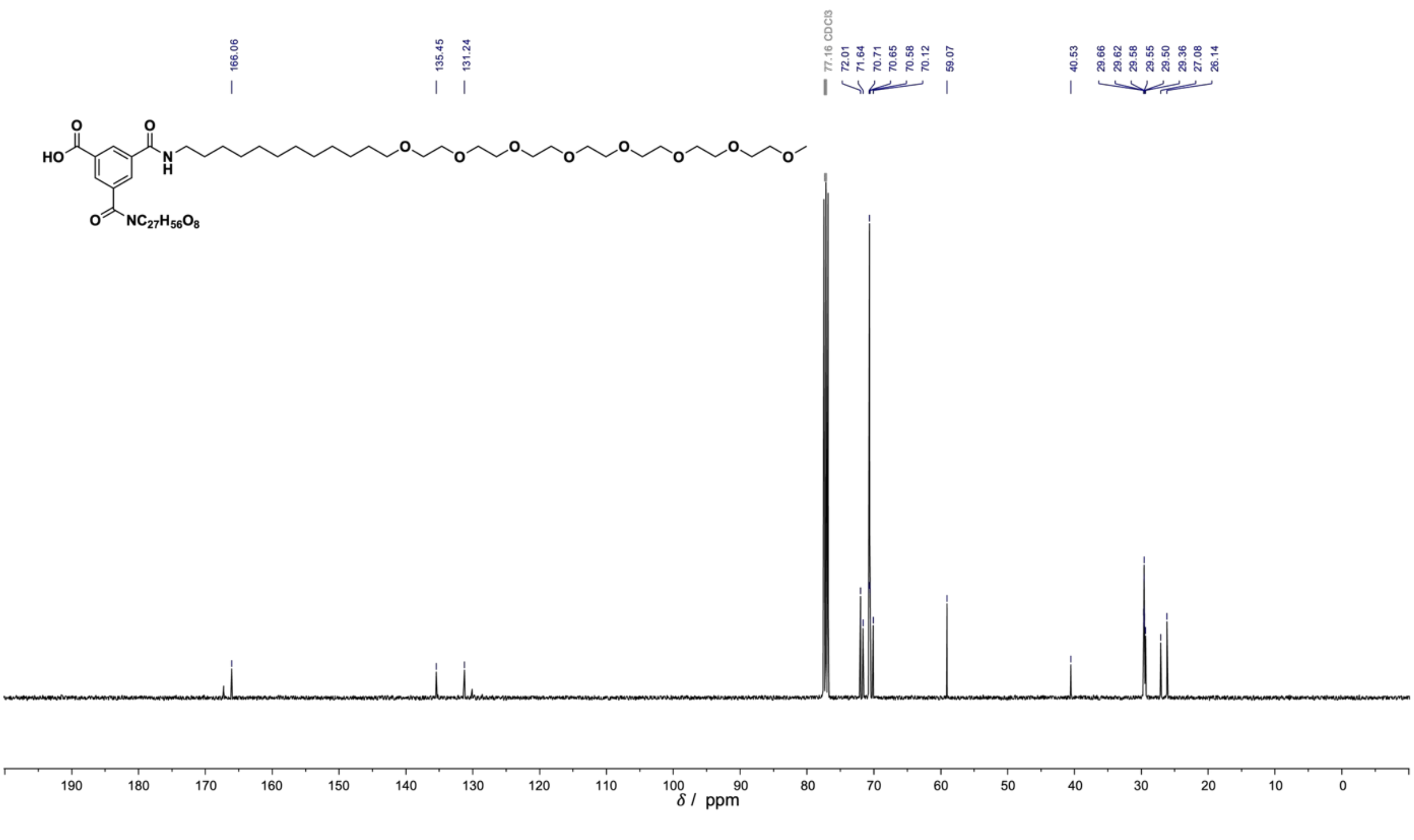

Fig. S32: ^{13}C NMR spectrum of compound **4** (101 MHz, 298 K, $CDCl_3$).

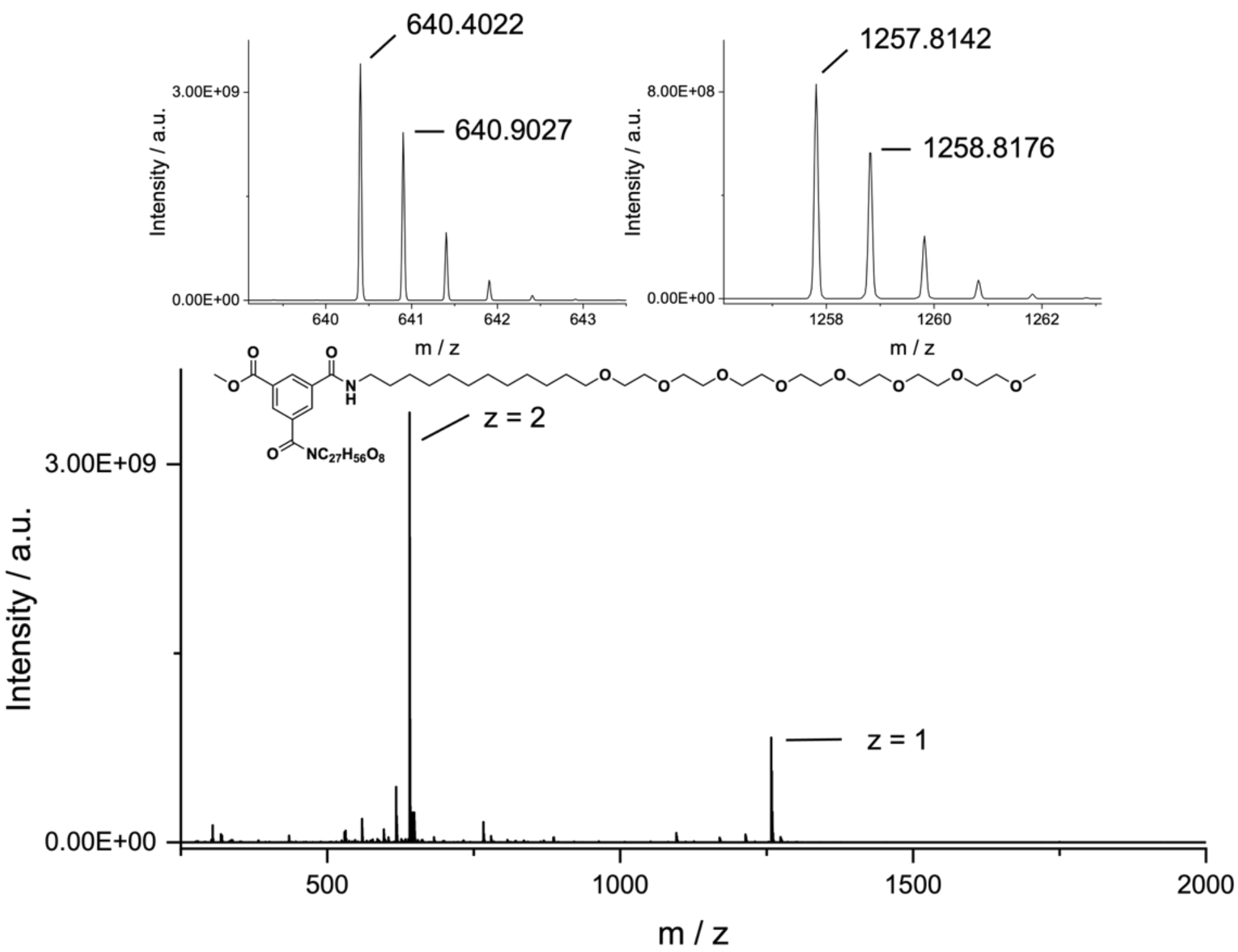

Fig. S33: HR-MS (ESI, positive) spectrum of compound **4**.

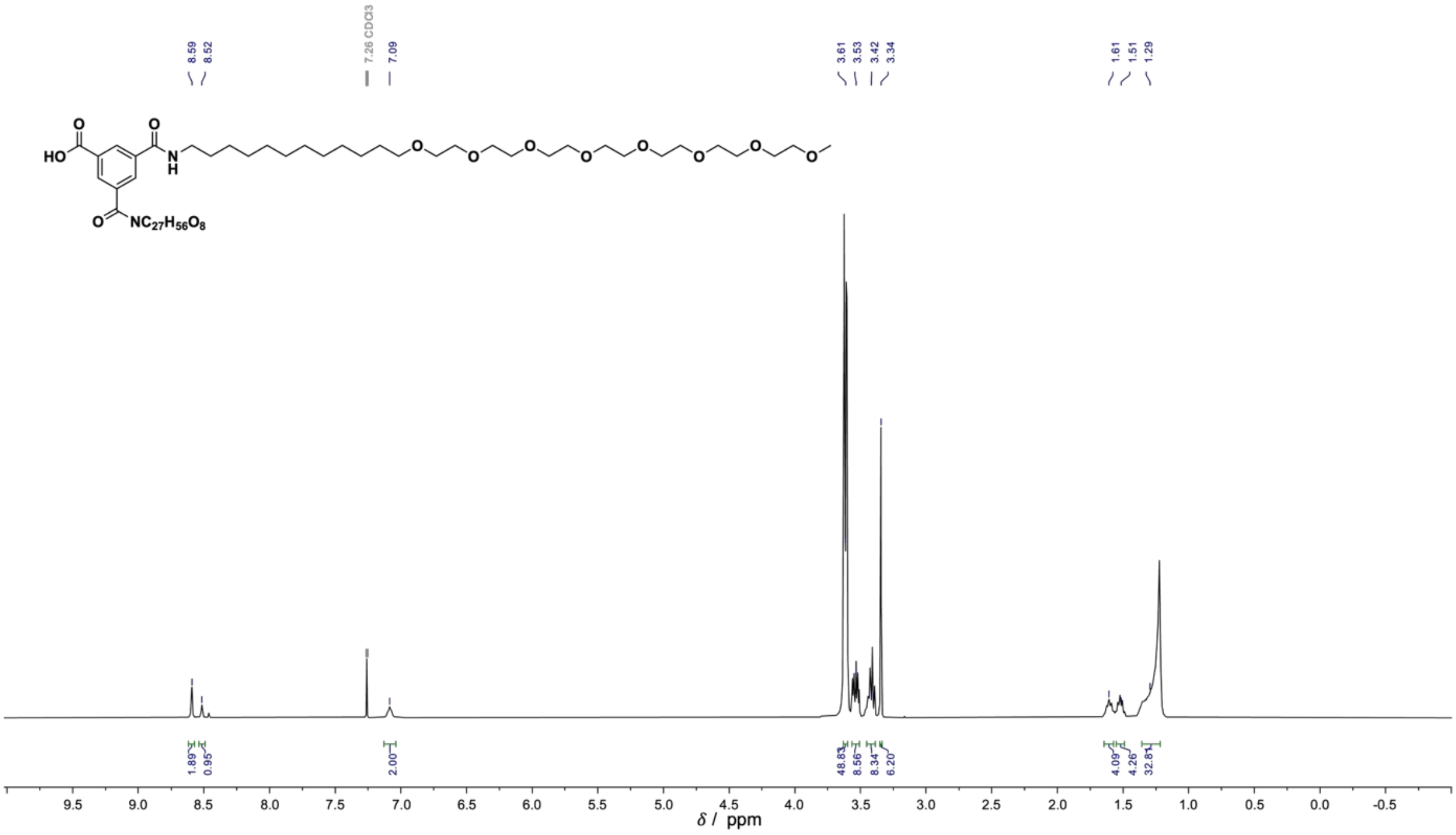

Fig. S34: ^{1}H NMR spectrum of compound **5** (400 MHz, 298 K, $CDCl_3$).

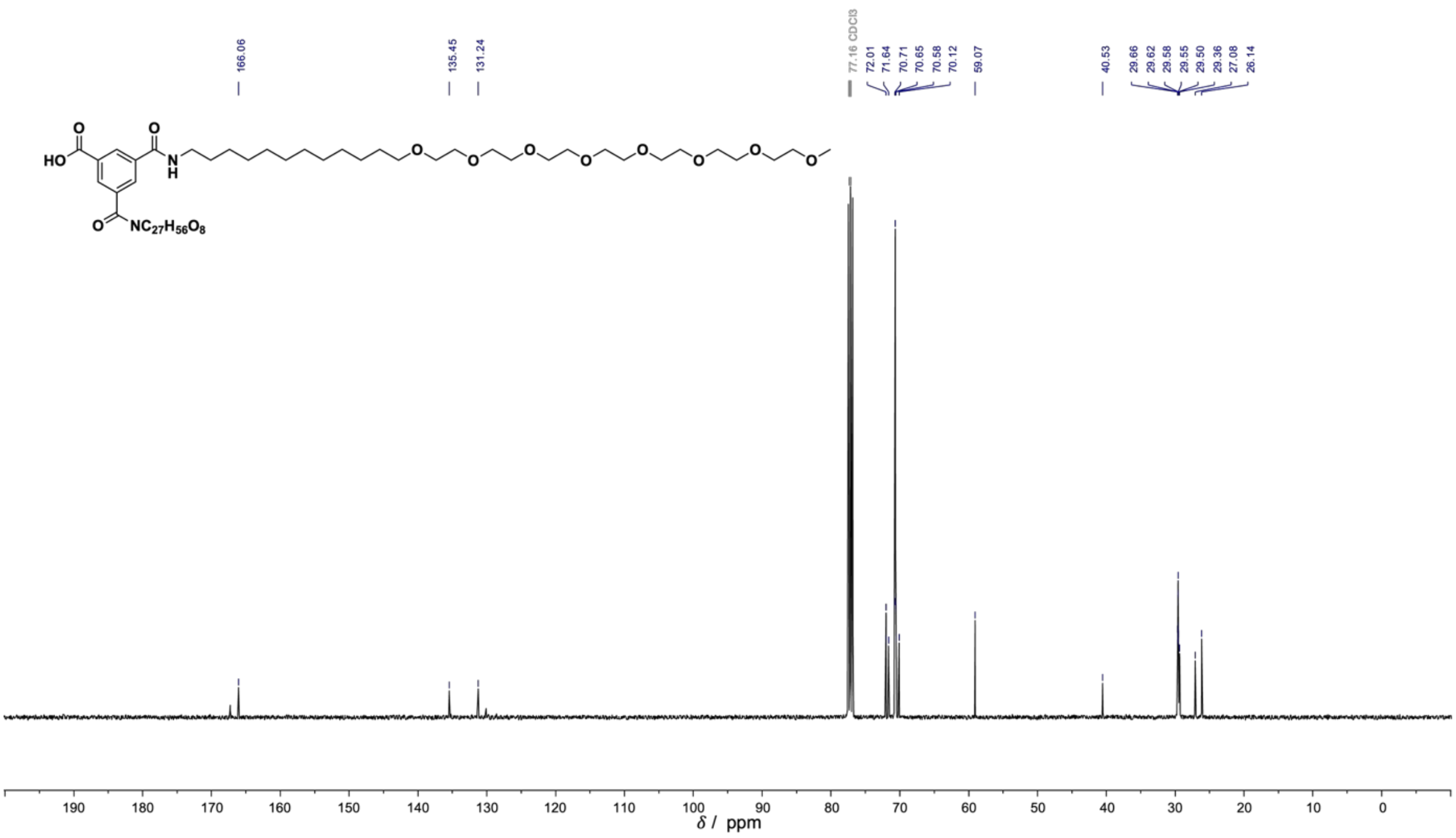

Fig. S35: ^{13}C NMR spectrum of compound **5** (101 MHz, 298 K, $CDCl_3$).

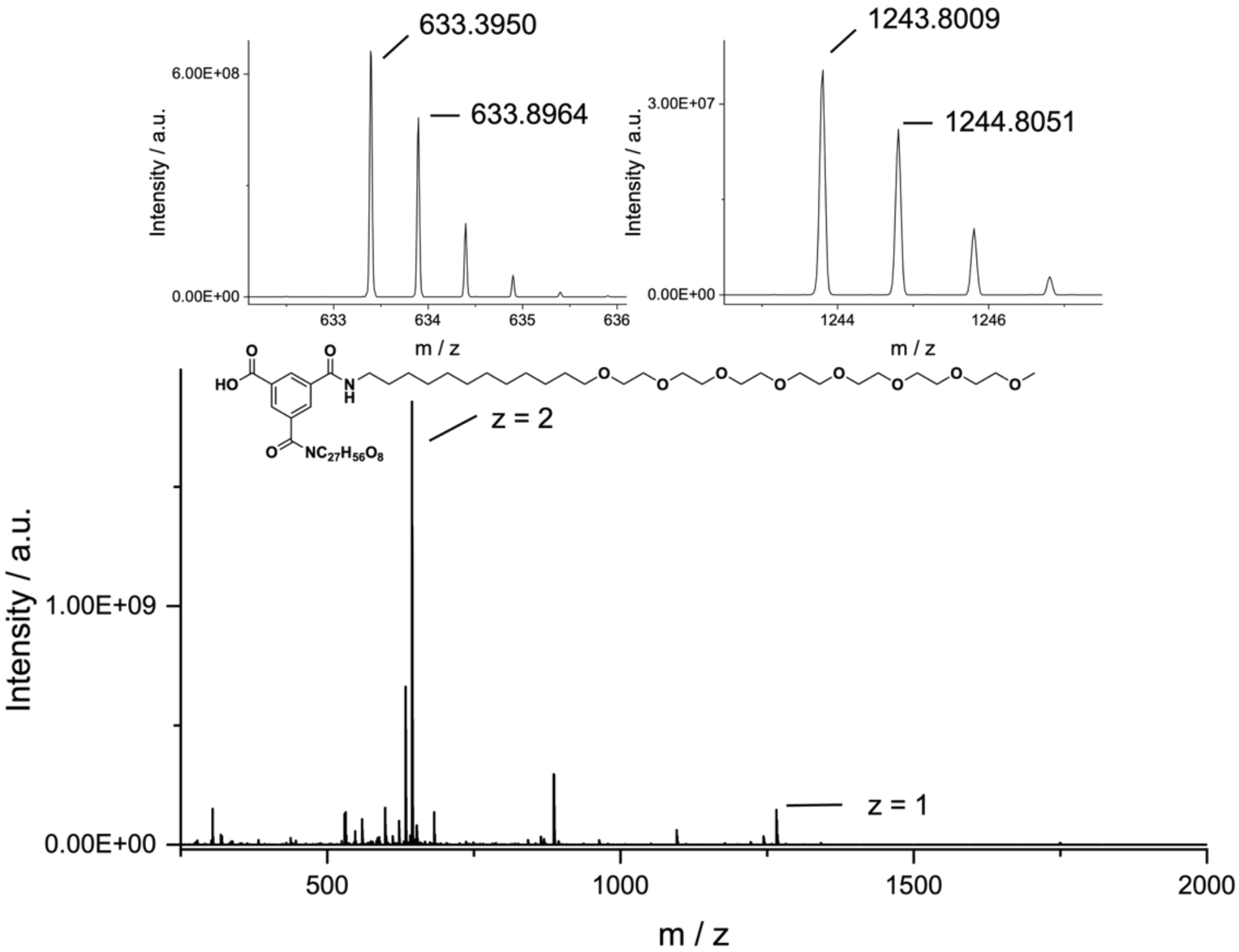

Fig. S36: HR-MS (ESI, positive) spectrum of compound **5**.

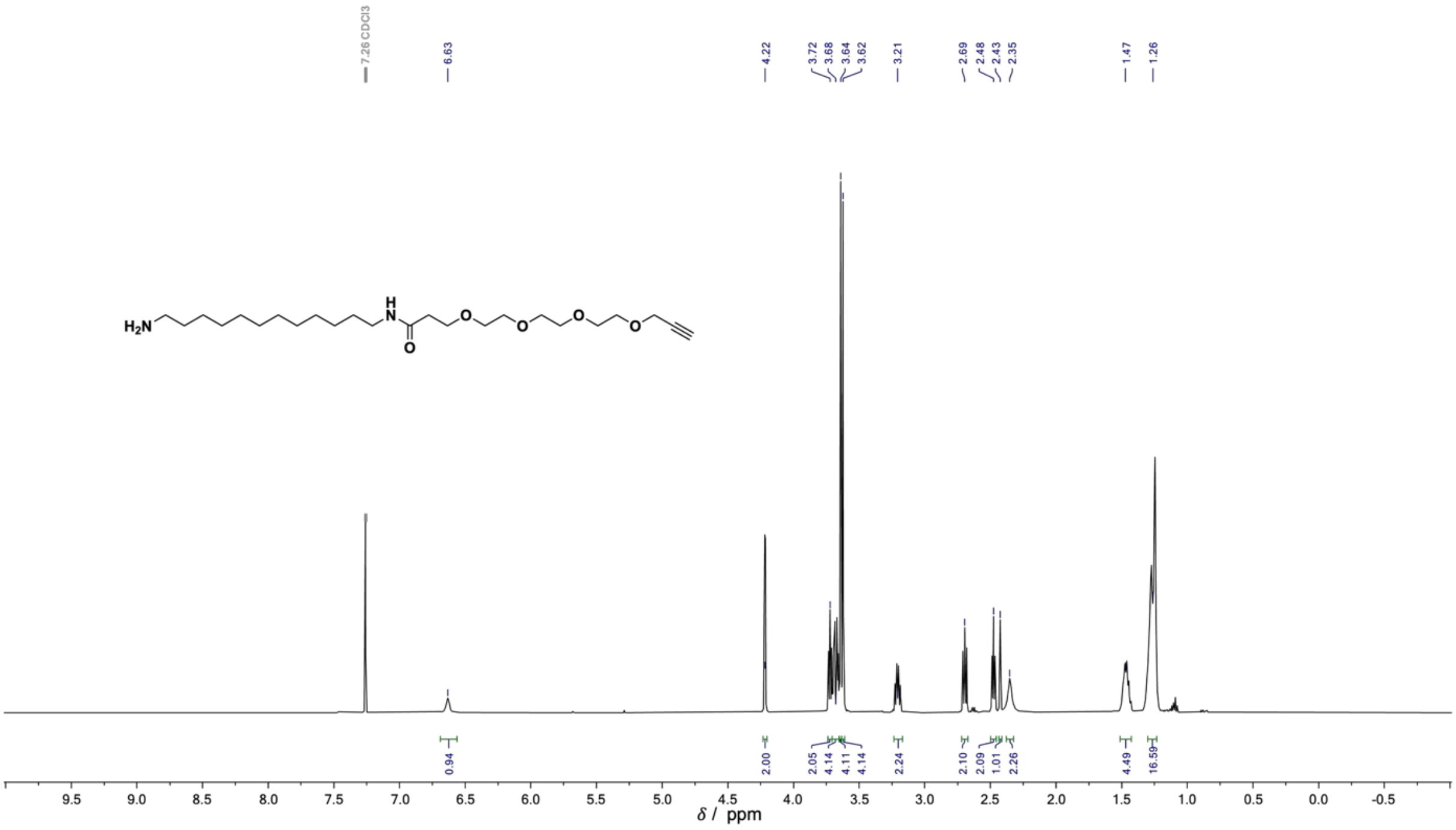

Fig. S37: ^{1}H NMR spectrum of compound **6** (400 MHz, 298 K, $CDCl_3$).

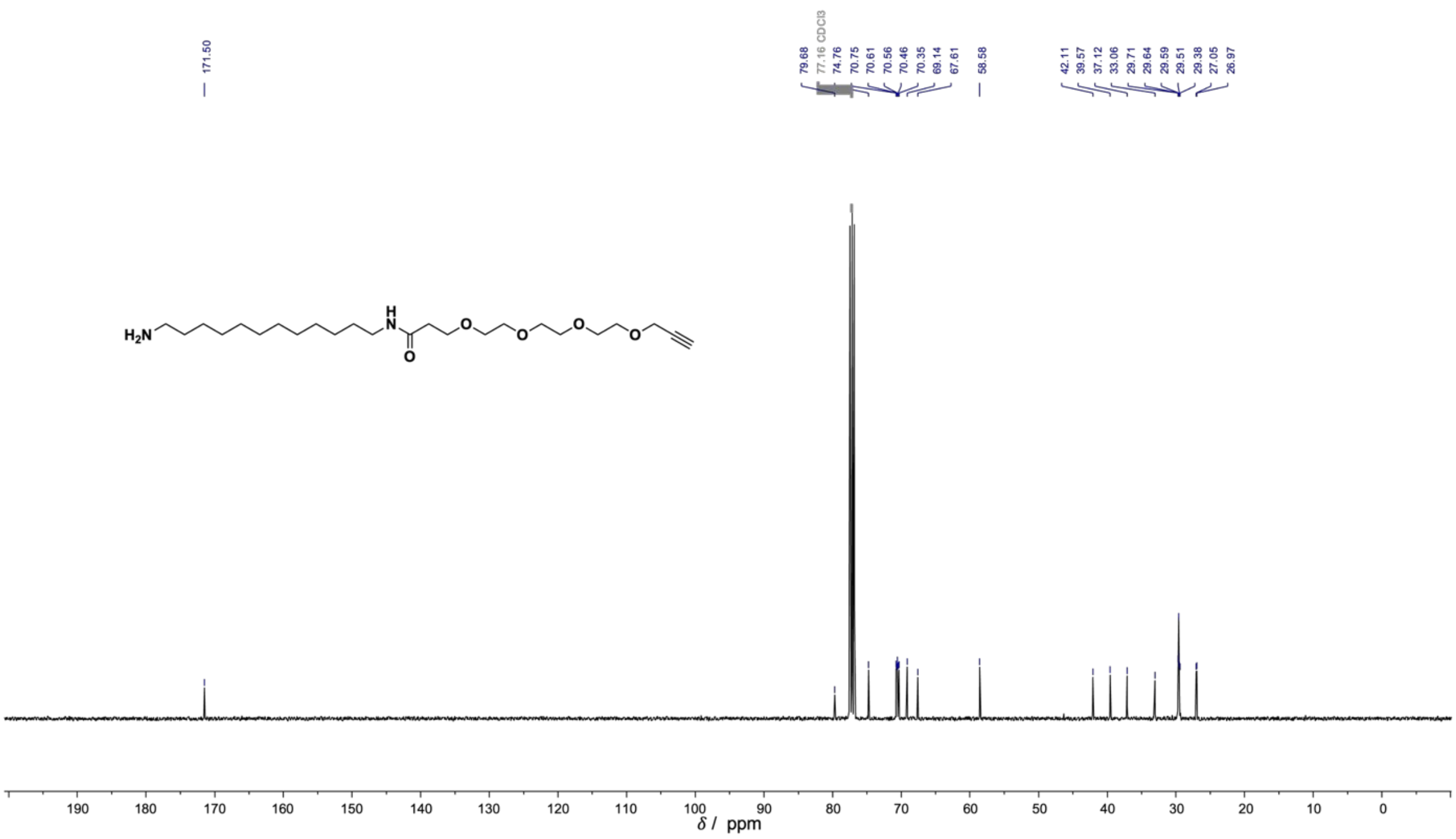

Fig. S38: ^{13}C NMR spectrum of compound **6** (101 MHz, 298 K, $CDCl_3$).

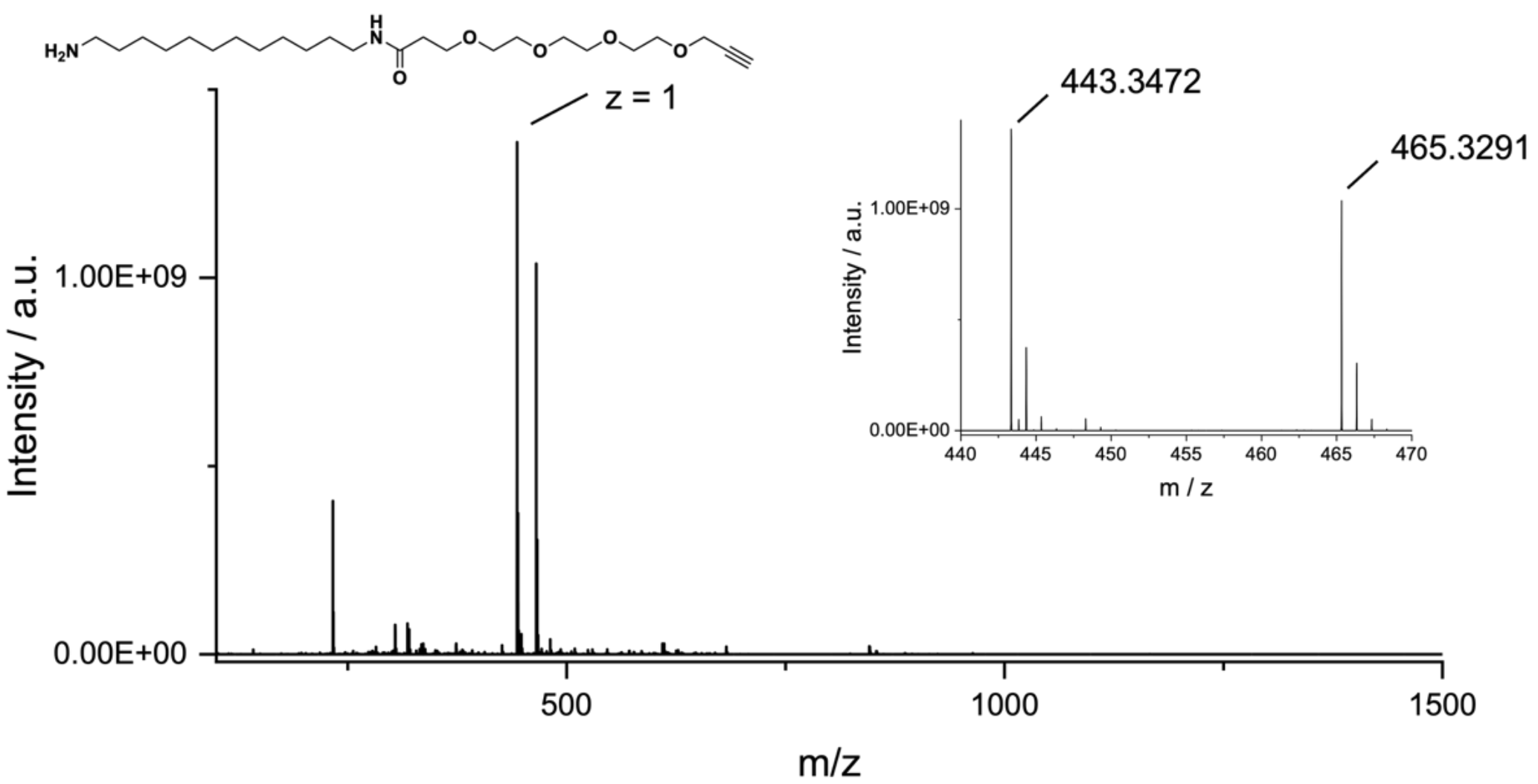

Fig. S39: HR-MS (ESI, positive) spectrum of compound **6**.

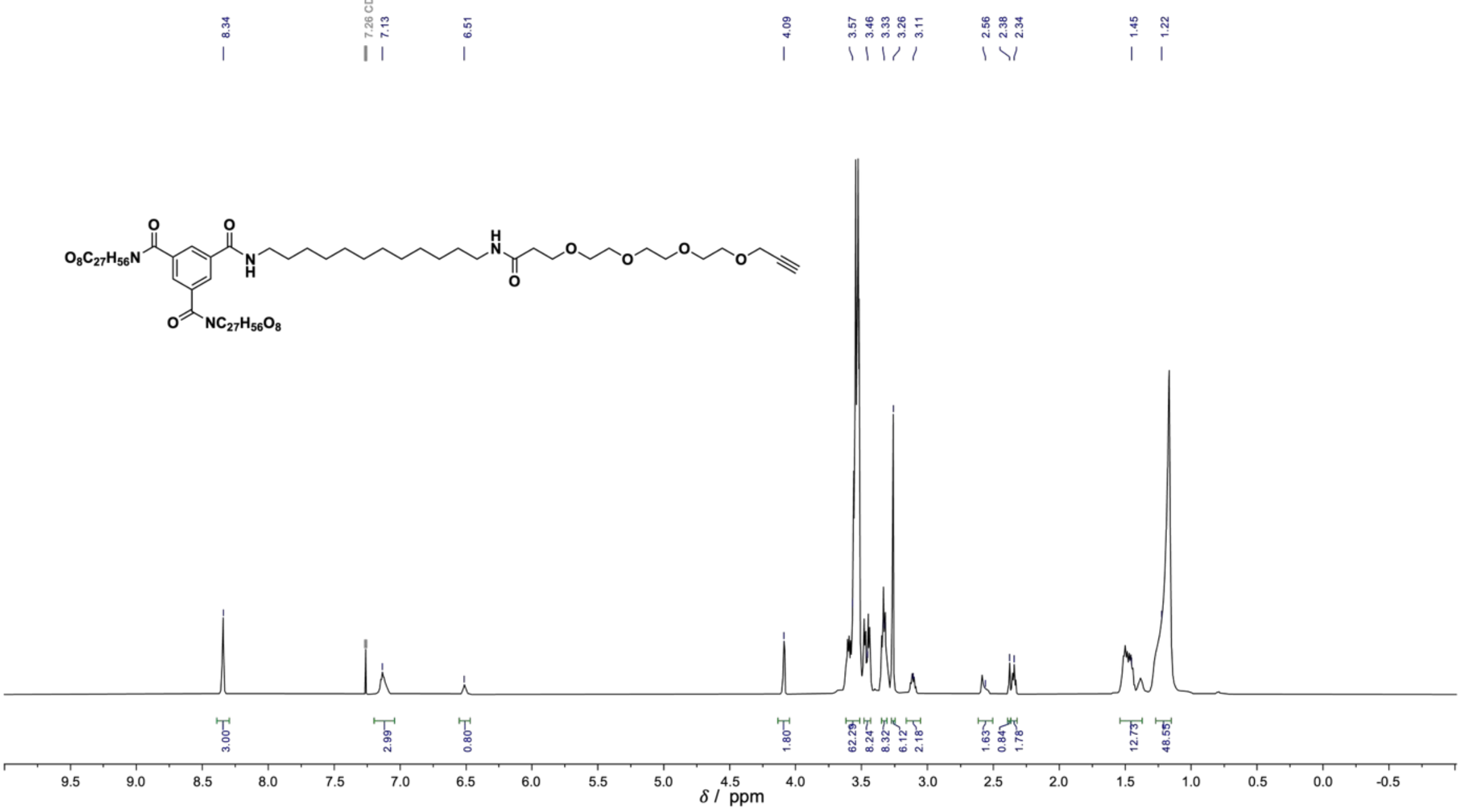

Fig. S40: ^{1}H NMR spectrum of compound **BTAAlkyne** (500 MHz, 298 K, $CDCl_3$).

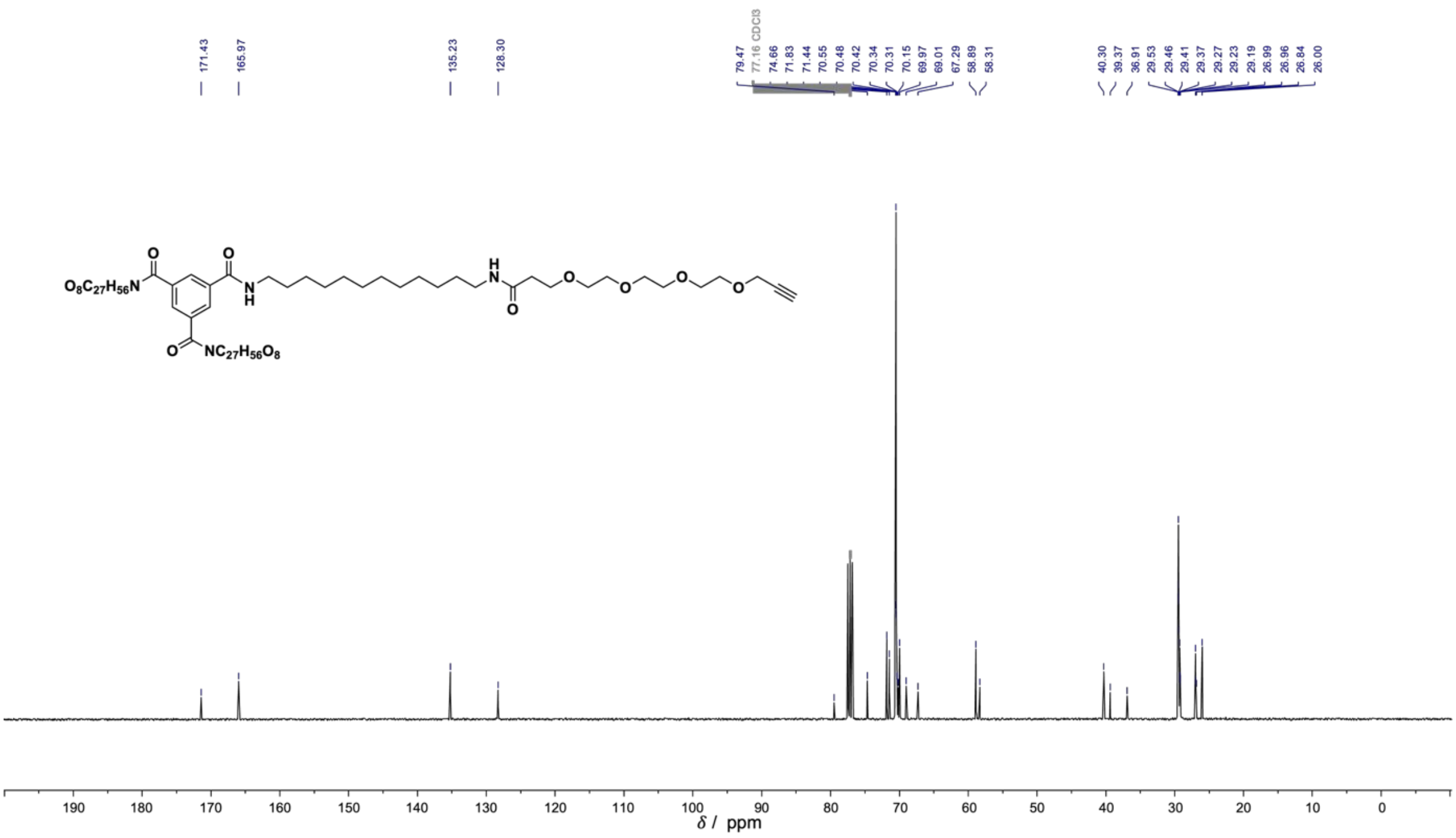

Fig. S41: ^{13}C NMR spectrum of compound **BTAAlkyne** (101 MHz, 298 K, $CDCl_3$).

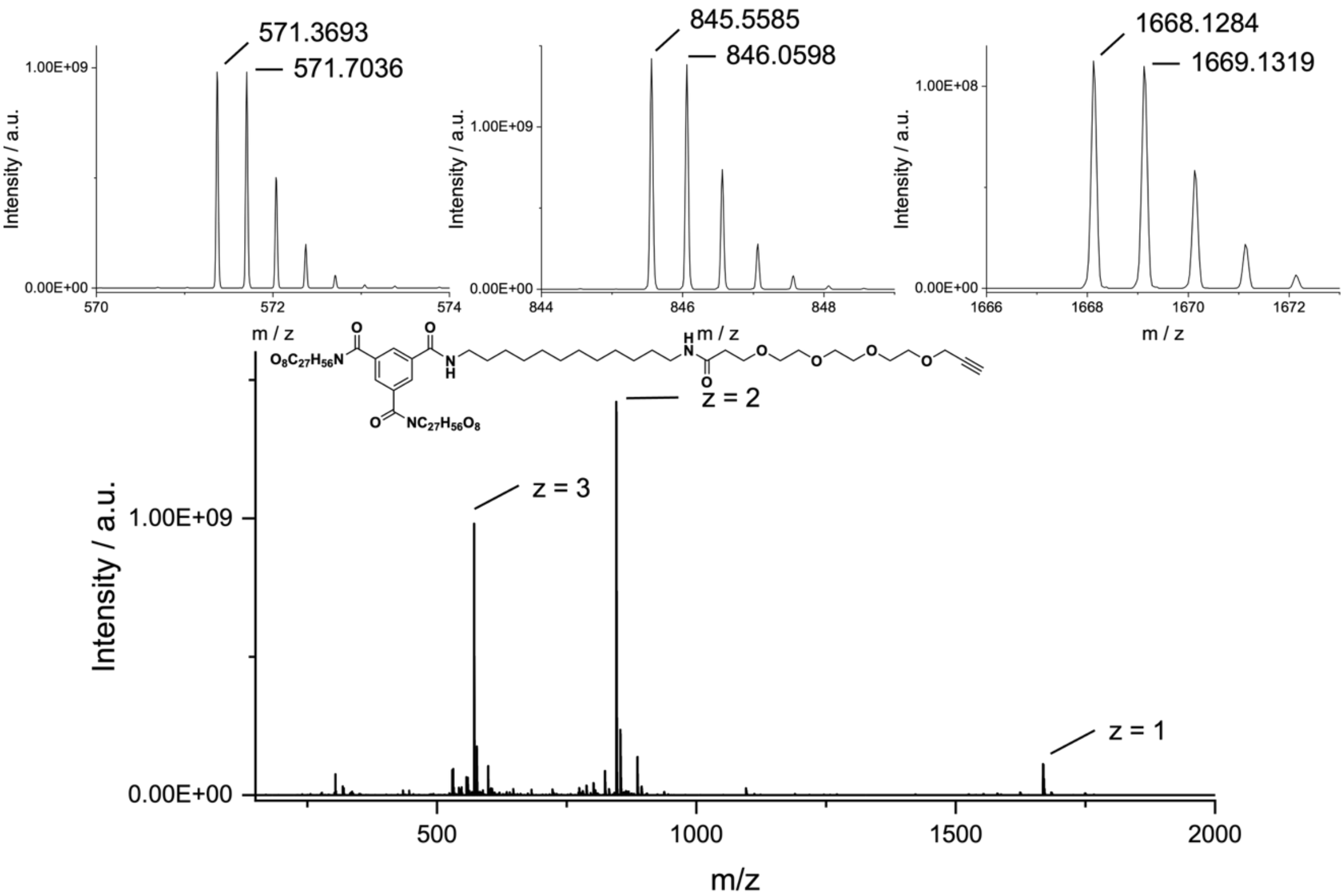

Fig. S42: HR-MS (ESI, positive) spectrum of compound **BTAAlkyne**.
